# Prospective Evaluation of a Rapid Clinical Metagenomics Test for Bacterial Pneumonia

**DOI:** 10.3389/fcimb.2021.684965

**Published:** 2021-10-19

**Authors:** Shengrui Mu, Long Hu, Ye Zhang, Yingmei Liu, Xiaojing Cui, Xiaohui Zou, Yeming Wang, Binghuai Lu, Shuilian Zhou, Xiaoxue Liang, Chen Liang, Nick Xiao, Justin O’Grady, Shela Lee, Bin Cao

**Affiliations:** ^1^ China-Japan Friendship Hospital, National Clinical Research Center for Respiratory Diseases, Clinical Center for Pulmonary Infections, Capital Medical University, Beijing, China; ^2^ Institute of Respiratory Medicine, Chinese Academy of Medical Sciences, Peking Union Medical College, Beijing, China; ^3^ Department of Pulmonary and Critical Care Medicine, Center for Respiratory Diseases, China-Japan Friendship Hospital, Beijing, China; ^4^ State Key Laboratory of Translational Medicine and Innovative Drug Development, Simcere Diagnostics Co., Ltd., Nanjing, China; ^5^ Tsinghua University-Peking University Joint Center for Life Sciences, Tsinghua University, Beijing, China; ^6^ Norwich Medical School, University of East Anglia, Norwich, United Kingdom; ^7^ Quadram Institute Bioscience, Norwich Research Park, Norwich, United Kingdom

**Keywords:** nanopore sequencing, lower respiratory infections, rapid diagnostics, bacterial pneumonia, pathogenic diagnosis

## Abstract

**Background:**

The diagnosis of bacterial pathogens in lower respiratory tract infections (LRI) using conventional culture methods remains challenging and time-consuming.

**Objectives:**

To evaluate the clinical performance of a rapid nanopore-sequencing based metagenomics test for diagnosis of bacterial pathogens in common LRIs through a large-scale prospective study.

**Methods:**

We enrolled 292 hospitalized patients suspected to have LRIs between November 2018 and June 2019 in a single-center, prospective cohort study. Rapid clinical metagenomics test was performed on-site, and the results were compared with those of routine microbiology tests.

**Results:**

171 bronchoalveolar lavage fluid (BAL) and 121 sputum samples were collected from patients with six kinds of LRIs. The turnaround time (from sample registration to result) for the rapid metagenomics test was 6.4 ± 1.4 hours, compared to 94.8 ± 34.9 hours for routine culture. Compared with culture and real-time PCR validation tests, rapid metagenomics achieved 96.6% sensitivity and 88.0% specificity and identified pathogens in 63 out of 161 (39.1%) culture-negative samples. Correlation between enriched anaerobes and lung abscess was observed by Gene Set Enrichment Analysis. Moreover, 38 anaerobic species failed in culture was identified by metagenomics sequencing. The hypothetical impact of metagenomics test proposed antibiotic de-escalation in 34 patients compared to 1 using routine culture.

**Conclusions:**

Rapid clinical metagenomics test improved pathogen detection yield in the diagnosis of LRI. Empirical antimicrobial therapy could be de-escalated if rapid metagenomics test results were hypothetically applied to clinical management.

## Introduction

Lower respiratory tract infection (LRI) is one of the top four causes of mortality worldwide (Naghavi et al., 2017). However, the identification of causative agents of LRI remains challenging due to the limitations of the current methodology. Conventional methods for diagnosing LRI, mainly using culture and serological tests, are insensitive and time-consuming (Holter et al., 2015). As a result, pathogens were only identified in 38% of adults who presented the radiographic evidence of pneumonia (Jain et al., 2015). The abuse of broad-spectrum antibiotics has made it even harder to identify pathogens, as patients may have already received antibiotics before the tests. A delayed diagnosis leads to inappropriate empiric, broad-spectrum antibiotic therapy, which causes poor therapy outcomes, longer hospital stays, and higher costs (Vaughn et al., 2019; Webb et al., 2019).

Unlike culture methods, molecular techniques identify pathogens by genetic molecules instead of microbe clones (Varadi et al., 2017). Although targeted techniques such as PCR are fast, they only allow the identification of carefully chosen pathogens (Hassibi et al., 2018; Poritz and Lingenfelter, 2018). Clinical metagenomics uses next generation sequencing of total nucleic acid from clinical samples to detected all the microbes simultaneously, allowing for unbiased pathogen identification that is less affected by clinical pre-judgement (Goldberg et al., 2015; Forbes et al., 2017; Blauwkamp et al., 2019; Wilson et al., 2019). Superior in terms of rapid library preparation and real-time data acquisition and analysis, the nanopore sequencing platform (Nanopore, Oxford, UK) has proven its ability to rapid LRI pathogen detection in recently studies (Charalampous et al., 2019). However, most previous studies were limited to individual patient or a series of small, retrospective cases (Pendleton et al., 2017; Moon et al., 2018; Yang et al., 2019). A question remains unclear: what is the performance and potential clinical value of applying rapid metagenomics in the diagnosis of common respiratory infections?

This was a prospective, single-center, on-site study involving hospitalized patients with community-acquired pneumonia (CAP), community-acquired pneumonia in immunocompromised host (CAP-ICH), hospital-acquired pneumonia (HAP), acute exacerbation of bronchiectasis (AEBX) (Woodhead et al., 2011), acute exacerbation of chronic obstructive pulmonary disease (AECOPD), and lung abscess, which were diagnosed based on guideline from American Thoracic Society, Chinese Thoracic Society, Infectious Diseases Society of America (**Supplementary File E1**). The study aimed to evaluate the clinical performance of a commercial rapid metagenomics test (Simcere Diagnostics, Nanjing, China), including turnaround time, pathogen identification rate, sensitivity, and specificity of on-site rapid metagenomic testing on bronchoalveolar lavage (BAL) or sputum samples collected from patients with LRIs.

## Materials and Methods

### Ethics Statement

The study was carried out in China-Japan Friendship Hospital, Beijing, China. Ethical approval was obtained from the China-Japan Friendship Hospital Ethics Committee(2018-145-k102). All subjects provided written consents.

### Study Design

Between November 2018 and June 2019, a cohort of 292 consecutively hospitalized patients suspected to have LRIs, including CAP, CAP-ICH, HAP, AEBX, AECOPD, and lung abscess, was enrolled after meeting the following inclusion criteria: Age ≥14 years, recent-onset/worsening cough, dyspnea, tachypnea, recent purulence or change in sputum characteristics, increased secretions or suctioning requirements, radiographic findings of new, progressive or persistent infiltrate (**Table 1**). BAL samples were collected from patients if bronchoscopy is necessary. Sputum was qualified by microscopy examination of gram staining slide: a sputum sample was qualified if they had <10 squamous cells and >25 leukocytes per low-power (×10) field. The on-site study involved collection of either BAL or qualified sputum sample, and parallel testing of these samples using routine microbiological techniques and rapid metagenomics.

**Table 1:** Demographic and clinical characteristics of the 292 patients.

Variable	N = 292
Age, median (IQR), years	64.0 (55.0,73.2)
Male, n (%)	201 (68.8)
Diagnosis at admission, n (%)	
CAP	83 (28.4)
HAP	66 (22.6)
Acute exacerbation of bronchiectasis	44 (15.1)
ICH^a^	44 (15.1)
AECOPD	33 (11.3)
Lung abscess^b^	22 (7.5)
Symptoms, n (%)	
Cough	243 (83.2)
Sputum	238 (81.5)
Fever	200 (68.5)
Dyspnea	78 (26.7)
Underlying Disease, n (%)	
Cardiovascular disease	123 (42.1)
Chronic respiratory disease	99 (33.9)
Diabetes	83 (28.4)
Immunosuppression^C^	60 (20.5)
Renal disease	34 (11.6)
Liver disease	22 (7.5)
Laboratory findings	
PCT≥0.25 ng/mL, n (%)	175 (59.9)
White blood cell count,median (IQR), × 10^9^/L	8.4 (6.0, 12.5)
Neutrophil count,median (IQR), × 10^9^/L	6.4 (4.1, 10.4)
Lymphocyte count,median (IQR), × 10^9^/L	1.1 (0.6, 1.6)
Platelet count,median (IQR), × 10^9^/L	206.0 (150.0, 284.0)
Hemoglobin,median (IQR), g/L	114.0 (94.0, 131.0)
ICU admission, n (%)	150 (51.4)
Invasive mechanical ventilation, n (%)	94 (32.2)
Current smoker, n (%)	76 (26.0)
Duration from admission to sequencing, median (IQR), days	3 (1,6)
Received antibiotic treatment before enrollment, n (%)	250 (85.6)
Length of hospital stay, median (IQR), days	
In ICU	15 (9,24)
In general ward	11 (9,14)
Outcome, n (%)	
Hospital discharge	237 (81.2)
Withdrawal of life support	19 (6.5)
In-hospital mortality	36 (12.3)

Categorical variables were presented as number (percentage).

Continuous variables were presented as median (IQR, inter quartile range).

CAP, Community Acquired Pneumonia; AECOPD, Acute exacerbation of chronic obstructive pulmonary; HAP, Hospital Acquired Pneumonia; PCT, Procalcitonin;

a Patients with CAP who are immunocompromised.

b Among the 22 patients with Lung Abscess, 2 were immunocompromised hosts.

c Immunosuppression: including 27 longterm steroid use, 24 receiving cancer chemotherapy, 5 solid organ transplantation, 2 primary immune deficiency diseases,2 receiving anti-rheumatic drugs or other immunosuppressive drugs.

### Reference Standard for Microbiological Diagnosis in This Study

Reference standard for microbiological diagnosis was defined as any positive result on routine microbiological culture, urinary antigen tests, or qPCR and Sanger sequencing tests. Respiratory tract samples from all 292 patients with LRIs underwent routine culture during their hospital stay. Urinary antigen tests were used for detection of *Streptococcus pneumoniae.* When rapid metagenomics results were discordant with culture or urinary antigen results, these pathogens were further verified by qPCR and Sanger sequencing (**Supplementary Table E1**). The primers and probes for qPCR and Sanger sequencing were shown in the **Supplementary Method**.

### Clinical Relevance and Appropriateness of Therapy

Patients’ medical records were assessed to determine whether the pathogens reported by rapid metagenomics were the potential cause of the clinical presentation. The appropriateness of the treatment regimen was assessed considering the treatment outcome and antibiotic regimen. The prescribed antimicrobial for each patient was compared with the hypothesized antimicrobial(s) which would be appropriate for pathogen-directed therapy based on the pathogen identified by metagenomic sequencing. The clinical features, radiologic and laboratory findings, antimicrobial use, and clinical improvement of each patient were independently reviewed by two clinicians (Dr. YL and XC). All patients were followed until discharge or death.

### Rapid Clinical Metagenomic Sequencing

Entire workflow of the rapid clinical metagenomic test were processed on-site including DNA extraction, host depletion, library construction and real-time sequencing and analysis (**Supplementary Figure E1** and **Method**). A species identified by rapid metagenomics with certain criteria met and contaminations eliminated was defined as a Meta-ID (**Supplementary Figure E2** and **Method**).

### Statistical Evaluation of Pathogen Identification Capacity

Meta-IDs on the common pathogen list used by routine microbiological tests were compared to the reference standard (**Supplementary Figures E1, E3**), to obtain statistics on diagnostic performance. Sensitivity, specificity, positive predictive value, negative predictive value, confidence intervals, positive concordance, negative concordance and pathogen identification rate were calculated using R software (v3.6.0). Gene set enrichment analysis (GSEA v4.0.3) (Subramanian et al., 2005) was performed on Meta-IDs against 6 types of diseases. See **Supplementary Methods** for more details.

## Results

### Overview of Subjects

The median age of the 292 involved patients was 64.0 years (IQR=55-73) and 68.8% of the subjects were male (**Table 1**). The cohort included patients with CAP (28%), CAP-ICH (15%), HAP (23%), AEBX (15%), AECOPD (11%), and lung abscess (8%). About half (51%) of the patients were from the ICU ward. Notably, a relatively high proportion of patients (85.6%) had received antibiotics before admission, reflecting the real-world scenarios (**Table 1**).

### Characteristics of Rapid Clinical Metagenomics Test

**Figure 1:**
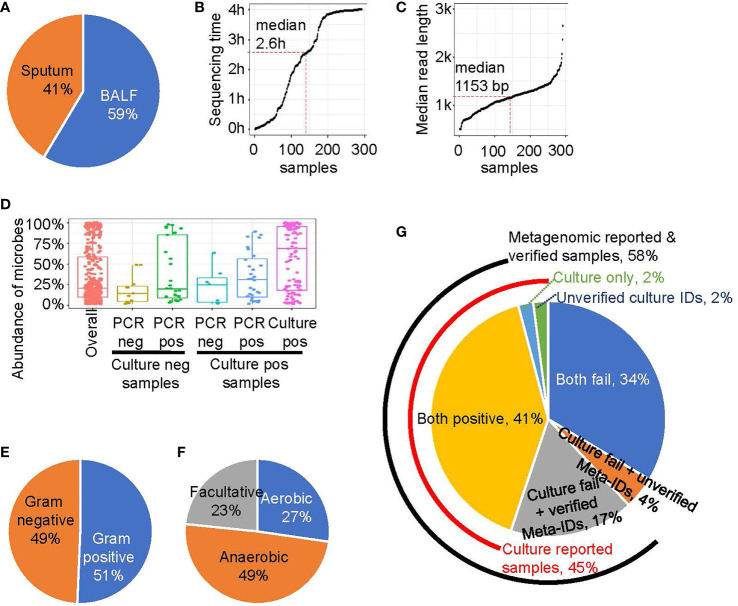
The rapid metagenomics assay characteristics. **(A)** Sample type distribution of this study. **(B)** The sequencing and analyzing time after library were loaded into sequencer. Each point represents a sample, ordered by sequence time. Median point is marked by red dash line. **(C)** The read length of a sample is represented by the median value of all reads’ length. Similar to B, median point is marked by red dash line. **(D)** The abundance of microbes that belongs to different groups: all microbes (red); experimentally unverified Meta-IDs from culture negative samples (brown); experimentally verified Meta-IDs from culture negative samples (green); experimentally unverified Meta-IDs from culture positive samples (light blue); experimentally verified Meta-IDs from culture positive samples (blue); culture reported Meta-IDs from culture positive samples (purple). **(E)** The Gram-stain of all identified microbes, except for *Chlamydia psittaci and Mycoplasma pneumoniae.* **(F)** The oxygen requirement of all identified microbes, except for *Chlamydia psittaci and Mycoplasma pneumoniae.* **(G)** A pie chart showing portion of different pathogen identification methods.

Each collected sample was divided into two aliquots for simultaneous routine microbiological testing and on-site rapid metagenomic sequencing. BAL samples were collected from 171 subjects (59%), and qualified sputum samples were collected from the remaining subjects (**Figure 1A**). The library construction method was optimized based on the fastest library preparation kit (ONT, Oxford, UK), making the total time required for library preparation, including sample DNA purification and loading, was less than 1 hour. In addition, as sequencing data are generated in real time during bioinformatics analysis, the reporting time was ≤ 4 hours when sufficient data were accumulated for pathogen identification. As the time required for microbe read number > 1,000 is typically less than 4 hours for some samples, the sequencing process was completed before the designed end-time and the median sequencing duration was 2.6 hours (**Figure 1B**). The overall turnaround time was 6.4 ± 1.4 hours. In contrast, the overall turnaround time for routine microbiological testing was 94.8 ± 34.9 hours (**Supplementary Figure E4**). The median read length of each sample is shown in **Figure 1C**. All reads used in this study were longer than 500 bp and most reads were in the range of 1,000-3,000 bp, with a median length of 1,152 bp. Furthermore, a previous study and our simulation analysis have demonstrated that the accuracy for identification of species is enhanced with longer read-lengths, even with relative poor single-base accuracy (**Supplementary Figure E5**).

We tested several threshold to define a Meta-ID and the result showed the threshold we chose is robust (**Supplementary Figure E2**). The abundance of Meta-IDs was highly related to its verification status (**Figure 1D**). In total, the abundance of all Meta-IDs ranged from 1% to 100%, with a median abundance of 20.6%. Meta-IDs of pathogens in culture-positive samples typically dominated or were in high abundance, with a median abundance of 69.1%; Meta-IDs missed in culture methods but verified by validation test exhibited medium abundance, with a median value of 31.0%, while Meta-IDs of unverified pathogen showed a median abundance of 25.0%; Meta-IDs verified using qPCR or sanger sequencing but culture-negative were typically in relatively low abundance of 20.0%, indicating the failure of culture methods possibly due to low abundance; Meta-IDs of experimentally-unverified pathogens in culture-negative samples were in low abundance, with a median abundance of 14.1% (**Figure 1D**).

In total, 80 microbe species were identified by rapid metagenomics (**Supplementary File E3**), among which about half of them are Gram-stain negative and another half is Gram-stain positive, with the exception of *Chlamydia psittaci, Mycoplasma pneumoniae*, and *Mycoplasma hominis*, which are not visualized by Gram-staining (**Figure 1E**). Meanwhile, anaerobes accounted for 49% of the total species identified by rapid metagenomics while none of them were reported by routine microbiological test (**Figure 1F**). Rapid metagenomics failed in 2 samples which reported *Acinetobacter nosocomialis* and *Leclercia adecarboxylata* by culture methods (**Supplementary Table E2**). Both species were found in the sequencing raw data but did not pass the Meta-ID criteria. To sum up, pathogens in 45% (n=131) were identified by culture methods, 58% (n=169) were reported by rapid metagenomics, 41% (n=119) were reported by both methods, and 17% (n=50) were verified by qPCR tests (**Figure 1G**). Pathogens in 2% (n=5) of the samples were only reported by culture but failed to be verified by qPCR tests. Pathogens in another 2% (n=5) of the samples were reported and verified by qPCR tests but not reported by rapid metagenomics. Both methods failed in 34% (n=98) of the total patients. Among ICU patients, pathogens were identified in 67% of the them using culture method plus rapid metagenomics. Furthermore, the culture method plus rapid metagenomics identified pathogens in 82% of HAP patients (**Supplementary Figure E6**).

### Performance of the Rapid Metagenomics Compared With Traditional Methods

In culture-positive samples, rapid metagenomics achieved a concordance ratio of 92.4% (121/131) in comparison with culture results (**Table 2**). With regards to the 10 samples with discordant results, 5 samples failed to be verified by qPCR tests; 3 samples showed low DNA concentrations (0.13, 0.87, and 1.3 ng/μL, respectively), and 2 samples failed to pass the pre-defined thresholds in metagenomic method.

**Table 2:** Statistical summary of rapid metagenomics compared with clinical microbiology and qPCR or Sanger sequencing.

Sample Type	Clinical Microbe^a^ Result	Meta Result	No. of Samples	Definition^c^	Category Description	Concordance with Clinical Microbe (95% CI)	Concordance with Clinical Microbe + 2° Method^b^(95% CI)
BALF (171)	Neg (96)	Neg (59)	59	TN	Completely identical	61.5% (59/96) (51.0%-71.2%)	90.6% (87/96) (82.9%-95.6%)
		Pos (37)	28	TP	qPCR/PCR+Sanger seq confirmed ≥ 1 ID		
			9	FP	qPCR/PCR+Sanger seq refuted all IDs		
	Pos (75)	Neg (6)	1	FN	qPCR/PCR+Sanger seq confirmed Culture ID	88.0% (66/75)(78.4%-94.4%)	93.3% (70/75) (85.1%-97.8%)
			2	FN	qPCR/PCR+Sanger seq results not complete		
			3	TN	qPCR/PCR+Sanger seq refuted all Culture IDs		
		Pos (69)	66	TP	Culture result confirmed, qPCR/PCR+Sanger seq confirmed ≥ 1 additional ID		
			1	TP	Culture result discordant, qPCR/PCR+Sanger seq confirmed ≥ 1 additional ID		
			1	FN	Culture result discordant, qPCR/PCR+Sanger seq results not complete		
			1	FP	Culture result discordant, qPCR/PCR+Sanger seq refuted all Culture and Meta-IDs		
Sputum (121)	Neg (65)	Neg (39)	39	TN	Completely identical	60.0% (39/65)(47.1%-72.0%)	93.8% (61/65) (85.0%-98.3%)
		Pos (26)	22	TP	qPCR/PCR+Sanger seq confirmed ≥ 1 ID		
			1	FP	qPCR/PCR+Sanger seq results not complete		
			3	FP	qPCR/PCR+Sanger seq refuted all IDs		
	Pos (56)	Neg (4)	2	FN	qPCR/PCR+Sanger seq results not complete	80.4% (45/56) (67.6%-89.8%)	96.4% (54/56) (87.7%-99.6%)
			2	TN	qPCR/PCR+Sanger seq refuted all Culture IDs		
		Pos (52)	45	TP	Culture result confirmed, qPCR/PCR+Sanger seq confirmed ≥ 1 additional ID		
			7	TP	Culture result discordant, qPCR/PCR+Sanger seq confirmed ≥ 1 additional ID		

a Clinical Microbe, culture or urinary antigen tests; Neg, negative; Pos, positive.

b 2° Method, verification by qPCR or Sanger sequencing.

c TN, true negative; TP, true positive FN, false negative FP, false positive; 95% CI, 95% confidence intervals.

In culture-negative samples, rapid metagenomics achieved a concordance ratio of 60.9% (98/161), in comparison with culture results (**Table 2**). For samples with discordant results, rapid metagenomics identified pathogens in all samples and 79.4% (50/63) of them had been experimentally verified, which improved the concordance ratio to 91.9% (148/161) compared with culture and validation results together. The remaining 13 samples failed to be verified by validation experiment. Overall, rapid metagenomic achived a sensitivity of 96.6%, specificity of 88.0%, overall positive predictive value (PPV) of 92.3%, and negative predictive value (NPV) of 94.5% (**Table 3**). Among the five LRI diseases, CAP achieved the highest performance, with sensitivity of 97.6%, specificity of 90.2%, PPV of 91.1%, and NPV of 97.4%. Diagnostic performance was similar between patients in ICU and those in the general ward, with sensitivity of 96.0% to 97.4%, specificity of 86.3% to 89.4%, PPV of 93.1% to 91.4%, NPV of 91.7% to 96.7%, respectively.

**Table 3:** Statistical performance of different group of patients, samples and diseases.

	True positive (#)	False positive (#)	False negative (#)	True negative (#)	Sensitivity (%, 95%CI)	Specificity (%, 95%CI)	Positive predictive value (%, 95%CI)	Negative predictive value (%, 95%CI)
overall	169	14	6	103	96.6 (92.3-98.6)	88.0 (80.4-93.1)	92.3 (87.2-95.6)	94.5 (87.9-97.7)
ICU	95	7	4	44	96.0 (89.4-98.7)	86.3 (73.1-93.8)	93.1 (85.9-97.0)	91.7 (79.1-97.3)
General wards	74	7	2	59	97.4 (90.0-99.5)	89.4 (78.8-95.3)	91.4 (82.5-96.2)	96.7 (87.6-99.4)
BALF	95	10	4	62	96.0 (89.4-98.7)	86.1 (75.5-92.8)	90.5 (82.8-95.1)	93.9 (84.4-98.0)
Sputum	74	4	2	41	97.4 (90.0-99.5)	91.1 (77.9-97.1)	94.9 (86.7-98.3)	95.3 (82.9-99.2)
AECOPD	19	1	1	12	95.0 (73.1-99.7)	92.3 (62.1-99.6)	95.0 (73.1-99.7)	92.3 (62.1-99.6)
CAP	41	4	1	37	97.6 (85.9-99.9)	90.2 (75.9-96.8)	91.1 (77.9-97.1)	97.4 (84.6-99.9)
CAP-ICH	25	2	1	16	96.2 (78.4-99.8)	88.9 (63.9-98.1)	92.6 (74.2-98.7)	94.1 (69.2-99.7)
HAP	51	2	2	11	96.2 (85.9-99.3)	84.6 (53.7-97.3)	96.2 (85.9-99.3)	84.6 (53.7-97.3)
AEBX	24	3	1	16	96.0 (77.7-99.8)	84.2 (59.5-95.8)	88.9 (69.7-97.1)	94.1 (69.2-99.7)
Lung abscess	9	2	0	11	100.0 (62.9-100.0)	84.6 (53.7-97.3)	81.8 (47.8-96.8)	100.0 (67.9-100.0)

Patient groups include: patients from ICU (intensive care unit) and from general wards.

Sample groups include: sputum and BALF (bronchoalveolar lavage fluid).

Disease groups include: AECOPD, Acute exacerbation of chronic obstructive pulmonary; CAP, community acquired pneumonia; CAP-ICH, community-acquired pneumonia in immunocompromised host; HAP, Hospital Acquired Pneumonia; AEBX, acute exacerbation of bronchiectasis and lung abscess.

95%CI: 95% confidence intervals, it was calculated in http://vassarstats.net/clin1.html#return.

### More Fastidious Pathogens Identified by Rapid Metagenomics

**Figure 2:**
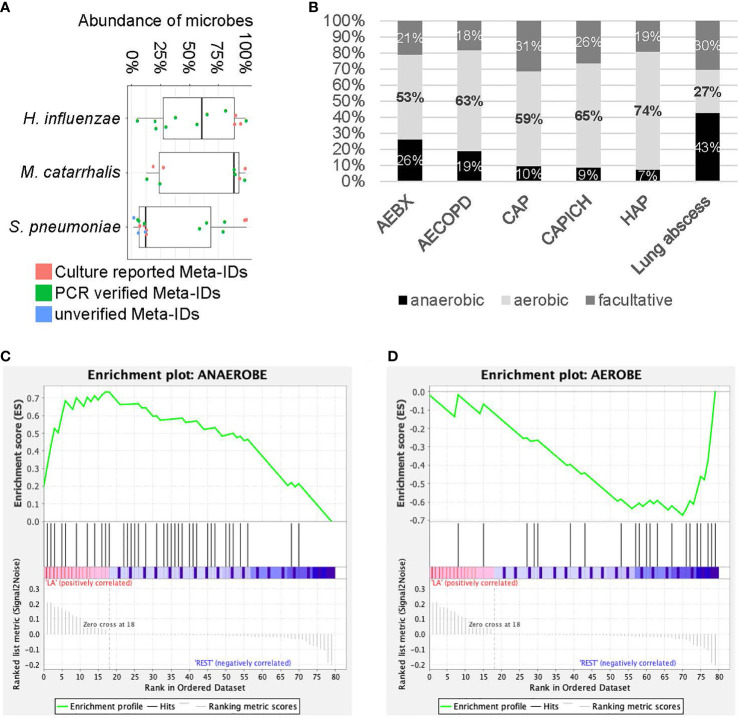
Analysis for fastidious species and anaerobic species. **(A)** The abundance of fastidious species. Each dot represents Meta-ID that belongs to different groups: culture verified Meta-IDs (red); experimentally verified Meta-IDs (green); experimentally unverified Meta-IDs (blue). **(B)** Portion of anaerobic, aerobic and facultative species in different diseases. **(C, D)** Gene set enrichment analysis for anaerobic **(C)** and aerobic **(D)** species in patients with lung abscess.

Identification fastidious pathogens are challeging for traditional methods, especially after exposure to antibiotics. Here we tested rapid metagenomics in three most common fastidious pathogens (Carroll, 2002; Kollef, 2006; Moran et al., 2013) i.e., *Streptococcus pneumoniae*, *Haemophilus influenzae*, and *Moraxella catarrhalis*. Rapid metagenomics identified fastidious pathogens in 37 cases, including *S. pneumoniae* (n=16), *H. influenzae* (n=12), and *M. catarrhalis* (n=9); while traditional methods identified in 13 cases, including 6 cases of *S. pneumoniae*, 3 cases of *H. influenzae*, and 4 cases of *M. catarrhalis*. Among the 11 cases with discordant results on *S. pneumoniae*, 7 were validated by qPCR, 3 failed, and 1 was not reported by rapid metagenomics as its abundance was below the cutoff value (only 1 reads in 4 hour data). The abundance of *S. pneumoniae* was relatively higher in samples with verified results than those without (**Figure 2A**). Moreover, samples with unverified *S. pneumoniae* tended to be dominated by high abundance of other commensal *Streptococcus* species. In total, *S. pneumoniae* was identified and verified in 5 patients with CAP, 2 patients with AECOPD, 2 patients with AEBX, 2 patients with HAP, 2 patients with CAP-ICH, and 1 patients with lung abscess (**Table 4**). One patient with CAP-ICH had their therapy deescalated because clinicians were made aware of a positive *S. pneumoniae* urinary antigen test.

**Table 4:** Results of rapid metagenomics, clinical diagnosis and medical records of patients with typical fastidious pathogens, *S. pneumonia*e, *H. influenzae* and *M. catarrhalis*.

Age | Sex | Sample type^a^	Clinical department^b^	Type of infection^c^	Comorbidities | Complication^d^	Empiric therapy |Switched therapy^e^	PCT^f^(ng/mL)	CRP^g^(mg/L)	Outcome | LOS^h^ (Days)^c^	Rapid metagenomics result	Comments^i^
**(Patients with *S. pneumoniae* identified by rapid metagenomics and urine antigen test)**									
57 | F | B	RICU	SCAP	None | Sepsis; RF	LVF+CRO+IPM | LVF; PIP/TAZ	5.48	372	Recovered | 23	*S. pneumoniae* (98.7%)	Early de-escalation would be achieved when clinicians knew Meta, as good as urinary antigen.
33 | M | B	RICU	SCAP	None | ARDS; RF	MFX	8.49	44.5	Recovered | 8	*R. mucilaginosa* (21.5%); *S. pneumoniae* (10.8%)	
44 | M | B	Ward	CAP-ICH	NS; Diabetes; Influenza | None	PIP/TAZ+MFX | AMC	0.58	11	Recovered | 9	*S. pneumoniae* (96.8%)	
**(Patients with *S. pneumoniae* identified by rapid metagenomics and culture)**									
70 | M | S	Ward	HAP (late-onset)	CTEPH; Chronic pulmonary heart disease | Cardiogenic shock	FOX | CSL	0.26	21	Dead | 15	*L. rhamnosus* (24.5%); *S. pneumoniae* (12.3%); *S. parasanguinis*(11.0%); *R. dentocariosa*(10.6%)	Narrow spectrum antibiotics could be chosen when *S. pneumoniae* was the most likely pathogen from Meta of’ sputum.
82 | M | S	Ward	CAP	Tuberculous pleurisy; GERD | None	LVF	0.28	19	Recovered | 11	*S. parasanguinis* (18.8%); *S. salivarius* (18.3%); *R. mucilaginosa*(15.4%); *S. pneumoniae* (6.7%)	
78 | M | S	Ward	AECOPD II	None	None	/	5	Recovered | 3	*C. argentoratense* (24.2%); *S. mitis* (20.0%); *S. pneumoniae* (12.3%)	
**(Patients with *S. pneumoniae* identified by rapid metagenomics and qPCR)**									
84 | M | B	RICU	SCAP	COPD; Chronic pulmonary heart disease; GERD | Sepsis; RF	CSL+AZM | CSL; LVF; VCM	5.36	39.1	Dead | 30	*S. pneumoniae* (58.3%)	De-escalation was not achieved because the clinicians did not know the causative pathogens.
75 | M | S	Ward	HAP (early-onset)	Cerebral infarction; Mental disorders | None	ETP	0.17	59	Recovered | 10	*S. pneumoniae* (64.3%)	
77 | F | S	Ward	AEBX	None	CSL	0.55	177	Recovered | 14	*S. mitis* (55.5%); *S. pneumoniae* (10.5%); *P. aeruginosa* (1.3%)	
56 | M | B	RICU	SCAP	Influenza | RF; ARDS	PIP/TAZ+LVF	9.89	38.07	Recovered | 19	*S. pneumoniae* (78.7%); *C. striatum* (6.7%)	
71 | M | B	Ward	Lung abscess	Metastatic carcinoma of lung; Colorectal cancer | None	CSL | PIP/TAZ	0.13	70	Recovered | 23	*S. pneumoniae* (80.0%)	
65 | M | S	Ward	AECOPD II	Lung cancer; GERD | None	None	/	24	Recovered | 29	*R. mucilaginosa* (16.9%); *S. pseudopneumoniae* (12.1%); *S. pneumoniae* (6%)	
52 | F | S	Ward	AEBX	GERD | None	PIP/TAZ	0.29	6	Recovered | 7	*P. aeruginosa* (53.5%); *S. mitis* (19.0%); *S. parasanguinis* (6.4%); *S. pneumoniae* (5.2%)	
**(Patients with *S. pneumoniae* identified by rapid metagenomics only)**									
59 | M | B	Ward	Lung abscess	Diabetes | None	PIP/TAZ+ORN	0.29	58	Recovered | 49	*P. aeruginosa* (24.3%); *S. mitis* (26.2%); *P. micra*(15.9%); *S. pneumoniae* (5.6%)	Non-verification might be low-abundance of *S. pneumoniae.*
88 | M | S	Ward	CAP-ICH	NHL; Chemotherapy; GERD; Lacunar infarction | Sepsis	MOX | MOX; LVF; MEPM; VCM	1.15	47	Recovered | 32	*S. mitis* (56.1%); *S. pneumoniae* (11.3%)	
69 | F | S	Ward	CAP-ICH	Multiple Myeloma; GERD | Cardiac failure; Pleural effusion	MOX | PIP/TAZ; LVF; AMK; TGC	0.69	23.5	Recovered | 21	*R. mucilaginosa* (27.2%); *L. pentosus* (14.2%); *V. parvula*(14.0%); *E. faecium* (3.1%); *S. pneumoniae* (1.7%)	
**(Patients with *H. influenzae* identified by rapid metagenomics and culture)**									
70 | F | B	Ward	CAP	Bronchiectasis | None	CRO	/	12	Recovered | 12	*H. influenzae* (93.2%)	Narrower spectrum antibiotics should be used.
74 | F | B	Ward	HAP (early-onset)	Sjögren’s syndrome; ILD | None	INH | INH; MFX	0.4	85	Recovered | 19	*H. influenzae* (88.4%); *M. intracellulare* (8.1%)	
65 | F | B	Ward	AEBX	None	None	0.26	6	Recovered | 6	*H. influenzae* (89.1%)	
**(Patients with *H. influenzae* identified by rapid metagenomics and qPCR)**									
60 | F | B	Ward	AEBX	None	CAZ	0.02	2	Recovered | 7	*H. influenzae* (98.1%)	Early de-escalation would be achieved if the clinicians knew Meta.
60 | M | B	RICU	AECOPD II	Chronic pulmonary heart disease | RF	PIP/TAZ+LVF	0.09	1.66	Recovered | 12	*H. influenzae* (80.6%)	
68 | M | S	Ward	AEBX	Diabetes | None	CAZ	0.26	7	Recovered | 8	*H. influenzae* (20.8%)	
81 | F | S	Ward	CAP	Chronic bronchitis; PAH; Chronic cardiac insufficiency | Pleural effusion	MFX | CAZ	0.21	3	Recovered | 16	*H. influenzae* (29.4%); *L. rhamnosus* (15.1%)	
38 | M | S	Ward	AEBX	Lung transplant; Emphysema | RF	PIP/TAZ	1.77	59	Recovered | 7	*C. striatum* (48.9%); *H. influenzae* (20.1%); *S. anginosus*(15.6%)	
43 | F | S	SICU	HAP (late-onset)	None | Brainstem hemorrhage	CRO | PIP/TAZ; CAZ	2.55	92.62	Recovered | 27	*H. influenzae* (64.4%); *P. aeruginosa* (16.4%)	*H. influenzae and P. aeruginosa* co-infection confirmed by both clinical diagnosis and Meta.
90 | M | S	Ward	AECOPD I	TB | None	CAZ | ETP; PIP/TAZ	13.8	53	Recovered | 13	*H. influenzae* (37.5%); *P. aeruginosa* (12.5%)	
60 | F | S	Ward	AEBX	AECOPD; PAH; Diabetes; Sjögren’s syndrome | None	None	0.3	5	Recovered | 11	*H. influenzae* (56.0%); *P. aeruginosa* (32.7%); *R. mucilaginosa* (5.7%)	
65 | M | B	Ward	CAP-ICH	Esophageal cancer; Chemotherapy | Myelosuppression	PIP/TAZ+TMP	0.23	5.41	Recovered | 9	*P. aeruginosa* (85.7%); *H. influenzae* (4.5%); *S. anginosus* (2.8%)	
**(Patients with *M. catarrhalis* identified by rapid metagenomics and culture)**									
56 | M | B	RICU	AEBX	Chronic pulmonary heart disease | RF	CIP+CAZ | CIP; CAZ; AZM	0.55	109.56	Recovered | 19	*M. catarrhalis* (97.4%)	*M. catarrhalis* in AEBX and AECOPD rapidly identified by Meta.
72 | M | S	Ward	AECOPD II	Chronic bronchitis | None	None	/	13	Recovered | 4	*M. catarrhalis* (92.4%); *R. mucilaginosa* (1.7%)	
54 | F | S	Ward	AECOPD II	None	None	/	7	Recovered | 17	*S. parasanguinis* (20.3%); *M. catarrhalis*(18.4%); *V. atypica* (13.0%)	
67 | M | S	Ward	AECOPD II	Chronic pulmonary heart disease | None	None	/	8	Recovered | 6	*P. aeruginosa* (69.1%); *M. catarrhalis* (27.3%)	
**(Patients with *M. catarrhalis* identified by rapid metagenomics and qPCR)**									
54 | M | B	RICU	SCAP	GRED | RF; Sepsis; Pleural effusion	PIP/TAZ | IPM; VCM	0.02	2	Dead | 5	*M. catarrhalis* (18.4%)	De-escalation was not achieved because the clinicians did not know the causative pathogens.
87 | M | S	Ward	AECOPD II	GRED; Cardiac insufficiency | None	ZOX	0.29	22	Recovered | 14	*M. catarrhalis* (87.9%); *R. mucilaginosa* (9.4%)	
62 | M | B	RICU	CAP-ICH	Rheumatoid arthritis; Rheumatoid lung disease; Diabetes; GRED; Cardiac insufficiency | None	ZOX+LVF	0.05	96.19	Recovered | 6	*M. catarrhalis* (24.0%); *V. atypica* (10.2%); *R. mucilaginosa* (8.4%)	
72 | M | S	Ward	AECOPD I	Bronchiectasis | RF	CSL+LVF	53	53	Recovered | 5	*M. catarrhalis* (88.8%); *R. mucilaginosa* (1.2%)	
58 | M | B	Ward	AECOPD II	Pulmonary interstitial fibrosis; Diabetes | None	MFX+CSL	0.21	3.41	Recovered | 13	M. catarrhalis (12.5%)	

a Gender: M, Male; F, Female. Sample type: S, Sputum; B, Bronchoalveolar lavage fluid.

b RICU, Respiratory intensive care unit; SICU, Surgical intensive care unit

c CAP, community acquired pneumonia; SCAP, severe community acquired pneumonia; HAP, hospital acquired pneumonia; AECOPD, acute exacerbation of chronic obstructive pulmonary disease, AnthonisenⅠ/Ⅱ; AEBX, Acute exacerbation of bronchiectasis

d CTEPH, chronic thromboembolic pulmonary hypertension; NS, Nephrotic syndrome; GERD, Gastroesophageal reflux disease; PAH, pulmonary arterial hypertension; TB, Tuberculosis; ILD, Interstitial lung disease; RF, Respiratory failure; NHL, non-Hodgkin’s lymphoma.

e If the antibiotics are not changed, only empirical therapy was reported.

AMC, Amoxicillin/clavulanic acid; AZM, Azithromycin; AMK, Amikacin; CAZ, Ceftazidime; CSL,Cefperazone/Sulbactam; CRO, Ceftriaxone; CIP, Ciprofloxacin; ETP, Ertapenem; FOX, Cefoxitin;

INH,Isoniazide; IPM, Imipenem; LVF, Levofloxacin; MFX, Moxifloxacin; MOX, Latamoxef; MEPM, Meropenem; ORN, Ornidazole; PIP/TAZ, Piperacillin/tazobactam; TGC, Tigecycline; TMP, Trimethoprim; VCM, Vancomycin; ZOX, Ceftizoxime.

f PCT, Procalcitonin.

g CRP, C-reactive protein.

h Length of stay.

i Meta, rapid metagenomics or identification results of rapid metagenomic.

All 12 patients positive for *H. influenzae* by metagenomics were also positive by the reference methods, i.e., 3 by culture method and 9 by qPCR, with a median abundance of 58% of the microbial reads. All 9 patients positive for *M. catarrhalis* by metagenomics were also positive by reference standard, plus 4 were verified by culture and 5 by PCR, with a median abundance of over 87% of the microbial reads (**Figure 2A**). *H. influenzae* was identified and verified in 12 patients including 2 with AECOPD, 5 with AEBX, 2 with HAP, and 1 with CAP-ICH, while *M. catarrhalis* was identified and verified in 9 patients, including 1 with CAP, 6 with AECOPD, 1 with AEBX, and 1 with CAP-ICH. Broad-spectrum antibiotics were empirically used in most patients, and no de-escalation was achieved as clinicians were not aware of the causative pathogens before prescription (**Table 4**).

### Higher Potential to Detect Anaerobic Species

Significantly more anaerobic species were identified by rapid metagenomics than by culture techniques (49% and 0%, respectively). Among the six types of LRI involved in this study, a large portion of anaerobic species (43%) and a small portion of aerobic species (27%) were identified in patients with lung abscess (**Figure 2B**). Subsequently, Gene Set Enrichment Analysis (GSEA) showed that anaerobic species were significantly enriched while aerobic species were significantly depleted in patients with lung abscess, with FDR < 0.25 (**Figures 2C, D**). As many of the anaerobic species are commensal bacteria in the upper respiratory tract, we performed additional enrichment analysis and the results showed similar association (**Supplementary Figure E7**). Finally, among the 50 patients with negative culture results, metagenomic results could be interpreted in 33 patients, as the probable or possible cause of their clinical presentation (**Supplementary File E5**) by adjudication definitions (Blauwkamp et al., 2019).

### Detection of Respiratory Viruses in Patients

Besides bacterial pathogens identified in these patients, viral infections were also detected in our patients. By reviewing clinical laboratory findings, among patients with both methods failed (n=98), 42% of patients got at least one virus detected in the 74 patients who were subjected to respiratory virus test. Among patients with positive culture results (n=131), 86 were tested for respiratory virus and 50% of them got at least one virus detected. Of the patients with positive results in nanopore test (n=169), 51% were positive for respiratory viruses among the 113 patients tested (**Supplementary File E4**). The most frequently detected viruses were Epstein-Barr virus (EBV) (n=47), human cytomegalovirus (HCMV) (n=33), influenza A (n=28), respiratory syncytial virus (RSV) (n=10), parainfluenza virus (n=2), adenovirus (ADV) (n=2), influenza B (n=1).

## Discussion

This study enrolled nearly three hundred patients to evaluate the performance of rapid metagenomics for the diagnosis of bacterial pathogens in LRI in the real-life scenarios, including patients from both ICU and general wards. Our study showed rapid metagenomics could improve the diagnositic yield for LRI, especially on pathogens that are difficult to culture, such as fastidious bacteria or anaerobes. For example, among the 34 patients with the three most common fastidious pathogens identified by rapid metagenomics, only 13 were reported by conventional methods. In fact, if the results of the metagenomics test had been used to guide therapy, 33 patients could have had their empiric therapy correctly de-escalated. In total, rapid metagenomics achieved an overall sensitivity of 96.6%, specificity of 88.0%, PPV of 92.3%, and NPV of 94.5%; the sensitivity and specificity were similar between patients from ICU and those from general wards. These performance characteristics make rapid metagenomics a very attractive solution for the rapid diagnosis of LRI.

Rapid metagenomics identified 38 anaerobic bacterial species in 49% samples, while none of the anaerobes were identified by culture. Although previous studies suggested that anaerobic bacteria are responsible for lung abscess (Hammond et al., 1995; Bartlett et al., 2000; Takayanagi et al., 2010), few anaerobes are cultured in clinical practice, and the treatment for lung abscess is depended on empirical broad-spectrum antibiotics without knowledge of the causative pathogens. Moving forward, if rapid clinical metagenomics was applied broadly on LRIs diagnosis, clinicians will have a growing amount of evidences identifying the bacteria associated with aspiration pneumonia and lung abscess, which is likely to increase the recovery rate of antibiotic therapy in these infections.

The turnaround time, from sample registration to pathogen identification, is much shorter for rapid metagenomics (6.4 ± 1.4 hours) than conventional culture-based diagnostics (94.8 ±34.9 hours). Considering the high mortality of ICU patients with severe LRI, a rapid diagnosis of causative pathogen is crucial for timely and appropriate antimicrobial therapy (Jain et al., 2015). With regards to the choice of antibiotics given to ICU patients with severe LRIs, intensivists believe that “broader is safer” (Waterer, 2019). Although antibiotics may be adjusted after the initial 72 hours of therapy (American Thoracic and Infectious Diseases Society of, 2005), intensivists are always reluctant to de-escalate without knowledge of causative pathogens (Morel et al., 2010; Joung et al., 2011; Heenen et al., 2012; Garnacho-Montero et al., 2014; Garnacho-Montero et al., 2015). The short turnaround time of rapid metagenomics will allow intensivists to be more confident in suspending broad-spectrum antibiotics earlier before causing additional adverse effects in patients. The current rapid metagenomic workflow required batch sequencing of several samples on one flowcell. To achieve the minimum turnaround time, metagenomic sequencing could be applied on low-throughput flowcell, such as Flongle, when a small number of sample need to be tested.

Though metagenomic sequencing achieved higher diagnositic yield, we still got 34% of samples with no pathogens identified in both metagenomic sequencing and traditional methods; we peculated that these patients might be of viral infection or non-infection respiratory diseases, as 42% of patients got respiratory virus detected. Furthermore, 50% of patients had both positive culture result and at least one virus detected. Interactions between viruses and bacteria in the pathogenesis of respiratory infections have been extensively reported in previous reports (Hament et al., 2004; Verkaik et al., 2011). For example, respiratory viruses could promote bacterial adhesion to respiratory epithelial cells, a process that may increase bacterial colonization and contribute to disease (Avadhanula et al., 2006). This findings suggested that viral infection should be considered in the design of future clinical metagenomic pipeline.

Limitations of this study should be noted. Firstly, more than 85% of the enrolled subjects had already received antibiotics before the collection of BAL or sputum samples, which may lead to bias in negative report of both culture methods and rapid metagenomic sequencing. Secondly, detecting a potential pathogen dosesn’t mean that we catched the causing agent of the diseases, as potential pathogens can be detected frequently in respiratory samples from people without respiratory symptoms. Thus, the full clincal pictures of the patients should be reviewed to determine the causive agents of LRTIs, including serum biomarker for infection, radiographic imaging, and clinical manifestation of patients. In our study, we involved two clincians to evaluate the potential role each species may play in the process of the disease, based on patient’s clinical data and guideline issued by ATS, CTS, IDSA and ESCMID (Takayanagi et al., 2010; Liu et al., 2012; Musher and Thorner, 2014; Di Pasquale et al., 2019). Moreover, the cost of test should be considered on the real clinical settings, as this rapid metagenomic test costed about 500$ per sample, which is much higher than that of traditional methods. Currently, metagenomic test is unlikely to replace conventional diagnostics in the near future but can be a complementary diagnositcs approach in certain clinical situations (Chiu and Miller, 2019), such as novel infectious diseases outbreak, critical patients with unexplained pathogens, and immunocompromised patients who are easily infected by uncommon pathogens which are not covered in conventional methods. (Xie et al., 2019; Yang et al., 2019; Wang et al., 2020; Azar et al., 2021). For these patients, timely detection of causing pathogens by the rapid metagenomic test could prevent delayed and inadequate therapy, prolonged stays, increased costs, and high mortality and morbidity (Graf et al., 2016).

In conclusion, we reported a rapid clinical metagenomics test for the untargeted detection of bacterial LRIs. It has been demonstrated to be faster and more sensitive than traditional diagnoisis methods, especially for fastidious and anaerobic bacteria, providing an understanding of the microbial community present in the respiratory sample and the relative abundance of the pathogen in that community. There is an urgent need for further carefully designed research to provide scientific evidences for the patient management and economic benefits that offered by this new technology in the clinical setting.

## Data Availability Statement

The datasets presented in this study can be found in online repositories. The names of the repository/repositories and accession number(s) can be found in the article/**Supplementary Material**.

## Ethics Statement

The studies involving human participants were reviewed and approved by China-Japan Friendship Hospital Ethics Committee(2018-145-k102). The patients/participants provided their written informed consent to participate in this study.

## Author Contributions 

Conception and design, BC. Acquisition of data, SM, LH, YZ, YL, XC, XZ, SZ, XL, CL, and YW. Analysis and interpretation of data, SM, LH, YZ, YL, JO’G, and SL. Drafting or revising of manuscript, LH, YZ, SM, YL, JO’G, and SL. Final approval of manuscript, LH, YZ, SM, YL, JO’G, SL, and BC. All authors contributed to the article and approved the submitted version.

## Funding

Funding provided by Ministry of Science and Technology (2018YFC1200102 and 2018YFE0102100), the CAMS Innovation Fund for Medical Sciences (CIFMS 2018-I2M-1-003), the UK-China Collaboration Fund to tackle AMR (Innovate UK (TS/S00887X/1), the Fundamental Research Funds for the Central Universities and Research projects on biomedical transformation of China-Japan Friendship Hospital (PYBZ1820) and the Ministry of Science and Technology of China (2017ZX10103004), the Biotechnology and Biological Sciences Research Council (BBSRC) Institute Strategic Programme Microbes in the Food Chain BB/R012504/1 and its constituent projects BBS/E/F/000PR10348 and BBS/E/F/000PR10349 (JO’G).

## Conflict of Interest

Authors LH, YZ, SZ, XL, CL and NX were employed by company Simcere Diagnostics Co., Ltd.

The remaining authors declare that the research was conducted in the absence of any commercial or financial relationships that could be construed as a potential conflict of interest.

## Publisher’s Note

All claims expressed in this article are solely those of the authors and do not necessarily represent those of their affiliated organizations, or those of the publisher, the editors and the reviewers. Any product that may be evaluated in this article, or claim that may be made by its manufacturer, is not guaranteed or endorsed by the publisher.
