# Supplementary material for: Prospective Evaluation of a Rapid Clinical Metagenomics Test for Bacterial Pneumonia
Source: Front Cell Infect Microbiol. 2021 Oct 19;11:684965. doi: 10.3389/fcimb.2021.684965 (PMC8560692; doi:10.3389/fcimb.2021.684965)
Supplement: Supplementary file 1 [file DataSheet_1.docx]

ONLINE DATA SUPPLEMENT

**Title:**

**Prospective Evaluation of a Rapid Clinical Metagenomics Test for Bacterial Pneumonia**

**Authors:**

Shengrui Mu^1,3,4*^, Long Hu^2*^, Ye Zhang^2*^, Yinmei Liu^1,3,4*^, Xiaojing Cui^1,3,4*^, Xiaohui Zou^1,3,4*^,Yeming Wang^1,4^,Binghuai Lu^1,3,4^ ,Shuilian Zhou^2^, Xiaoxue Liang^2^, Chen Liang^2^, Nick Xiao^2^, Justin O’Grady^6,7^, Shela Lee^2**^ and Bin Cao^1,3,4,5**^; CAP-China Network.

Affiliations:

1 China-Japan Friendship Hospital, National Clinical Research Center for Respiratory Diseases, Clinical Center for Pulmonary Infections, Capital Medical University, Beijing, China.

2 State Key Laboratory of Translational Medicine and Innovative Drug Development, Simcere Diagnostics Co., Ltd., Nanjing, China.

3 Institute of Respiratory Medicine, Chinese Academy of Medical Sciences, Peking Union Medical College, Beijing, China.

4 Department of Pulmonary and Critical Care Medicine, Center for Respiratory Diseases, China-Japan Friendship Hospital.

5 Tsinghua University-Peking University Joint Center for Life Sciences, Beijing, China.

6Norwich Medical School, University of East Anglia, Norwich, UK.

7Quadram Institute Bioscience, Norwich Research Park, Norwich, UK.

*These authors contributed equally to this work.

**Supplemental Methods**

**Reference standard**

In this study, the reference standard for microbiological diagnosis was defined as any positive of routine microbiological culture, urinary antigen tests, qPCR followed by Sanger sequencing tests.

***Routine microbiological culture:*** Respiratory tract samples from all 292 patients with suspected LRIs underwent routine culture during their hospital stay. BALF or sputum samples were incubated at 35°C with 3-5% CO_2_ on MacConkey agar, sheep blood agar, and chocolate agar. Bacterial isolates from patients were identified using VITEK^®^2 Compact (bioMerieux, Marcy-l' Etoile, France) and the identity of all isolated species was further validated via matrix-assisted laser desorption/ionization time of flight (MALDI-TOF) mass spectrometry (Bruker Daltonics, Billerica, MA, USA). The list of common pathogens reported by the routine microbiology laboratory includes: *Achromobacter xylosoxidans*, *Acinetobacter baumannii, Acinetobacter nosocomialis, Acinetobacter pittii, Burkholderia cepacia, Burkholderia multivorans, Corynebacterium striatum, Enterobacter cloacae, Escherichia coli, Haemophilus influenzae, Klebsiella aerogenes, Klebsiella oxytoca, Klebsiella pneumoniae, Leclercia adecarboxylata, Moraxella catarrhalis, Pseudomonas aeruginosa, Staphylococcus aureus, Stenotrophomonas maltophilia, Streptococcus pneumoniae*.

***Urinary antigen testing:*** Urinary antigen testing for *Streptococcus pneumoniae* was performed using a commercially available kit (BinaxNOW™ *Streptococcus pneumoniae*).

***Validation of discordant results by qPCR or Sanger sequencing:*** All qPCR assays were performed on QuantStudio™ 5 Real-Time PCR System (Applied Biosystems, Foster City, CA, USA). SYBR Green primers, TaqMan probes and target pathogens listed in **Supplemental Table E1** (Oligonucleotides were supplied by Thermo Fisher Scientific, Carlsbad, CA, USA). The master mix of probe-based reactions consisted of 7.5μL SGExcel GoldStar TaqMan Master (Sangon Biotech Co., Ltd., Nanjing, China), 0.4μL each of reverse and forward primers (final conc. 0.2μM), and 0.4μL probe (final conc. 0.2μM). For all qPCR based on SYBRGreen, the master mix consisted of 7.5μL FastStart Universal SYBR Green Master (Roche Diagnostics, Indianapolis, IN, USA), and 0.4μL each of reverse and forward primers (final conc. 0.2μM). Each reaction consisted of 2μL of DNA template and nuclease-free water was added to a total volume of 15μL. PCR products were then verified by agarose electrophoresis analysis and subsequently subjected to forward and reverse Sanger sequencing at Sangon Biotech Co., Ltd. QPCR and Sanger sequencing were used as third-party validation method to disentangle the discordancy between culture results and rapid metagenomics. Pathogens in samples with discordant culture results were preferentially verified by qPCR and Sanger sequencing, as the sample volume were limited. However, to prove such validation strategy was unbiased, when sample volume is sufficient, pathogens from culture and metagenomic positive samples were also tested by qPCR and Sanger sequencing. In this study, total 81 cases that reported positive by both culture and rapid metagenomics had been tested by qPCR and Sanger sequencing: 71 (87.6%) of them could be verified, suggesting that verification for discordant cases were enough.

**Clinical relevance and appropriateness of therapy**

Patients’ medical records were assessed to determine whether the pathogens reported by rapid metagenomics were the likely cause of the clinical presentation. The appropriateness of the treatment regimen was assessed considering the treatment outcome. The clinical features, radiologic and laboratory findings, antimicrobial use, and clinical improvement of each patient were independently reviewed by two clinicians. All patients were followed until discharge or death.

**Rapid nanopore-based metagenomic sequencing**

The on-site processing of clinical samples included 3 hours for sample preparation, 1 hour for library construction, and up to a maximum of 4 hours for sequencing and analysis (**Supplemental Figure E1**). Sequencing was run until >1000 microbial reads were identified in all the samples on the flow cell (except for the negative control) or to a maximum of 4 hours.

***Host depletion, DNA extraction, and library construction*:** BALF (1.5 mL) and sputum (400 μL) samples were collected and stored at 4°C before testing. Samples were treated using an optimized version of a previously reported sample preparation protocol[1]: for host DNA depletion in sputum the final saponin concentration was reduced to 0.1% and HL-SAN DNase volume was reduced to 3μL. The BALF depletion procedure was further optimized by increasing input sample volume to 1.5mL, reducing the HL-SAN DNase to 1μL, and two centrifugation steps were removed. Following host DNA depletion, the final pellet was re-suspended in 750 μL of bacterial lysis buffer (4659180001, Roche Diagnostics, Indianapolis, IN, USA), transferred to a bead-beating tube (Lysis Matrix E, MP Biomedicals, Santa Ana, CA, USA), and bead-beaten at 6 m/s for 40 s in FastPrep-24™ 5G Instrument (MP Biomedicals, Santa Ana, CA, USA). Samples were then centrifuged at 14,000 g for 5 min and DNA was extracted from the supernatant using Maxwell® RSC Whole Blood DNA Kit in Maxwell® RSC instrument (Promega Corp, Madison, WI, USA). DNA quantification was performed using the Quant-it high-sensitivity dsDNA assay kit on Qubit 3.0 Fluorometer (Invitrogen, Waltham, MA, USA). DNA libraries for nanopore-based sequencing were constructed using the Rapid Barcoding Kit (SQK-RBK004, Oxford Nanopore Technologies (ONT) Ltd., Oxford, UK) according to the manufacturer's instructions. Equal quantities of tagmented DNA for up to six samples were pooled and the total library (400 - 900 ng) was loaded on a R9.5 flow cell and sequenced on the GridION X5 platform (ONT Ltd., Oxford UK) according to manufacturer's instructions.

***Real-time sequencing and bioinformatic analysis:*** The sequencing process was controlled through ONT MinKNOW software (Version 3.3.2). ONT Guppy (Version 3.0.3) was used for the base-calling of raw sequenced data (fast5 files) into reads (fastq files). Sequencing reads were demultiplexed by blastn-short (Versions 2.7.1+), followed by removal of short (read length ≤ 500 nt) and low-quality reads (mean q-score ≤ 8). Subsequently, host reads were removed by aligning reads to the human reference genome (GRCh38) using Minimap2 (Version 2.14-r883). The remaining reads were assigned to taxonomy using Centrifuge software (Version 1.0.4) and validated by Megablast (Version 2.7.1). Reads with alignments to multiple bacterial species were excluded from further analysis. Abundance was calculated as the number of reads of a microbe divided by the number of total reads of all microbes.

***Definition of Meta-ID:*** The Simcere metagenomic test output is described as the Meta-ID and is defined as follows: for species in the common pathogen list (see above section of ***Routine microbiological culture***), the top two most abundant species or all species with abundance over 10% are considered as the Meta-ID; for species not on the list of common pathogens, the top three most abundant species or all species with abundance over 10% were considered as Meta-ID and such thresholds had been proved robust (**Supplemental Figure E2**). The following thresholds were applied to remove contamination, misclassification and barcode crosstalk:

1) To reduce barcode crosstalk (misclassification of barcodes during demultiplexing), we excluded Meta-IDs with read number ≤10% of the of the same species from other samples in the same run (e.g. if there were 100 *S. pneumoniae* reads in barcode 2 and 9 *S. pneumoniae* reads in barcode 4, barcode 4 would not be considered positive for *S. pneumoniae*).

2) ONT sequencing produces lower single-read accuracy compared to NGS at an error rate of ~10%, which can lead to the misclassification of related species within the same genus. To reduce such error, we used only reads with unique-alignment and excluded any Meta-IDs with read number ≤10% the read number of a species within the same genus.

3) DNA extraction and library preparation reagents, plastics and the laboratory environment can contaminate the sequencing library with a low abundance species.[2] To overcome this contamination issue, any species with abundance <1% or read number lower than 5 were not considered as Meta-IDs.

**Gene set enrichment analysis**

Gene set enrichment analysis (GSEA, v4.0.3) is a state-of-art analysis tool for enrichment analysis. GSEA was originally designed to reveal enrichment of any gene set in different conditions (or phenotypes), using these genes’ expression matrix. In this study, species were categorized into three "gene sets" based on their oxygen requirements (i.e., anaerobe, aerobe, and facultative). Patients with different diseases were "phenotype". The microbial abundance matrix of any species in different patients was used as "gene expression matrix". Recommended significant thresholds of false discovery rate (FDR) value < 0.25 and nominal *p*-value < 0.05 were used in this study.[3]

**Statistical evaluation of pathogen identification comparison**

Meta-IDs on the common pathogen list from routine clinical microbiology tests were verified by qPCR or Sanger sequencing (**Supplemental Figure E1**), to obtain statistics on diagnostic performance. A sample was defined as a true positive (TP) when at least one Meta-ID was verified by at least one of the following four microbiology testing methods, culture, urinary antigen tests, qPCR, and Sanger sequencing. A sample was defined as a false positive (FP) when none of its Meta-IDs could be verified by any one of the microbiology testing methods. A sample was defined as a false negative (FN) when culture or antigen tests reported pathogens were confirmed by qPCR/Sanger sequencing but not reported by rapid metagenomics. A sample was defined as a true negative (TN) when there was no Meta-ID and no pathogen was reported by culture or antigen tests, or all culture or antigen tests reported pathogens were refuted by qPCR/Sanger verification.[1,4] (**Supplemental Figure E3**).

Sensitivity was calculated as TP / (TP + FP); specificity was calculated as TN / (TN + FP); positive predictive value was calculated as TP / (TP + FP); negative predictive value was calculated as TN / (TN + FN).

Concordance between rapid metagenomics results and reference standard are evaluated (**Table 2**). Negative concordance between rapid metagenomics and clinical microbiology results (culture or urinary antigen tests) was defined as number of rapid metagenomics negative samples divided by total number of clinical microbiology negative samples. Negative concordance between rapid metagenomics and clinical microbiology plus secondary verification methods (qPCR or Sanger sequencing) was defined as sum of TN and TP samples divided by total number of clinical microbiology negative samples. Positive concordance between rapid metagenomics and clinical microbiology results was defined as number of rapid metagenomics positive samples with Meta-ID identical to clinical microbiology results, divided by total number of clinical microbiology positive samples. Positive concordance between rapid metagenomics and clinical microbiology plus secondary verification methods (qPCR or Sanger sequencing) was defined as sum of TN and TP samples divided by total number of clinical microbiology positive samples.

The pathogen identification rate by culture techniques was defined as the number of culture-positive samples divided by the total number of samples. Pathogen identification rate by rapid metagenomics was defined as the number of true positive samples divided by the total number of samples. Pathogen identification rate by culture plus rapid metagenomics was defined as the sum of culture-positive and -negative samples with verified Meta-ID divided by the total number of samples.

Supplemental Table E1. qPCR primers and probes used in this study

| Organism | **Gene target** | **Forward primer** | **Reverse primer** | **Probe** | **Reference** |
| --- | --- | --- | --- | --- | --- |
| *Achromobacter xylosoxidans* | *blaOxa-114* | CACGAGCCGGTCTGGAA | GTGAATACCAGACCACCGAATAC | [FAM]TACCAGCCYGCCTATCCCGACT[BHQ1] | Rapid detection of four non-fermenting Gram-negative bacteria directly from cystic fibrosis patient's respiratory samples on the BD MAX™ system. |
| *Acinetobacter baumannii* | *ompA* | TCTTGGTGGTCACTTGAAGC | ACTCTTGTGGTTGTGGAGCA | [FAM]AAGTTGCTCCAGTTGAACCAACTCCA[BHQ1] | Rapid detection of antibiotic resistance in Acinetobacter baumannii using quantitative real-time PCR |
| *Acinetobacter pittii* | *gyrB* | GATAACAGCTATAAAGTTTCAGGTGGT | CAAAAACGTACAGTTGTACCACTGC | SYBR Green | gyrB Multiplex PCR To Differentiate between *Acinetobacter calcoaceticus* and *Acinetobacter* Genomic Species 3 |
| *Burkholderia cepacia* | *TonB* | GACTGGGAATTGCGCCATAC | CTTCGCGACGTAGCTTTGC | SYBR Green | Accurate identification of members of the *Burkholderia cepacia*complex in cystic fibrosis sputum |
| *Burkholderia multivoran* | *EGY20_19360* | GATGGAGGGCAACGGACTTT | CTTCAGATACTCGCGTGCCA | SYBR Green | Designed in house |
| *Corynebacterium striatum* | *A9D01_00835* | CTTCGAAGAACATGAAGGCA | CCGTAGTACATCGCTACGGC | SYBR Green | Efficient differentiation of Corynebacterium striatum, Corynebacterium amycolatum and Corynebacterium xerosis clinical isolates by multiplex PCR using novel species-specific primers |
| *Enterococcus faecalis* | *16S rRNA* | CCGAGTGCTTGCACTCAATTGG | CTCTTATGCCATGCGGCATAAAC | SYBR Green | Prevalence of putative virulence factors and antimicrobial susceptibility of *Enterococcus faecalis*isolates from patients with dental Diseases |
| *Enterococcus faecium* | *ddl* | TTGAGGCAGACCAGATTGACG | CGGAAGTGATGCTTCCTACTG | SYBR Green | Vancomycin-Resistant Enteroccus Faecium and Enterococcus FaecalisIsolated from Education Hospital of Iran |
| *Escherichia coli* | *cyaA* | CGATAATCGCCAGATGGC | CCTAAGTTGCAGGAGATGG | [VIC]TAGAGCGCCTTCGGTGTCGGT[BHQ1] | Nanopore metagenomics enables rapid clinical diagnosis of bacterial lower respiratory infection |
| *Haemophilus influenzae* | *omp P6* | AGCGGCTTGTAGTTCCTCTAACA | CAACAGAGTATCCGCCAAAAGTT | SYBR Green | Characterisation of gut, lung, and upper airways microbiota in patients with non-small cell lung carcinoma |
| *Klebsiella aerogenes* | *OprD* | CGTGCGATACCAAGTACCTATCA | TATATTCCGTCGTCCATGTCACC | Sanger sequencing | Designed in house |
| *Klebsiella oxytoca* | *pehX* | TACCGTCACGCACTATCCTC | TCAAGCGGATACTGGGCC | SYBR Green | Speciation of common Gram-negative pathogens using a highly multiplexed high resolution melt curve assay |
| *Klebsiella pnenmoniae* | *16S rRNA* | ACGGCCGAATATGACGAATTC | AGAGTGATCTGCTCATGAA | SYBR Green | Detection of Bacterial 16S rRNA and Identification of Four Clinically Important Bacteria by Real-Time PCR |
| *Moraxella catarrhalis* | *16S rRNA* | GTCAAACAGCTGGAGGTATTGC | GACATGATGCTCACCTGCTCTA | [VIC]ATCGCAATTGCAACTTT[BHQ1] | A Reservoir of Moraxella catarrhalis in Human Pharyngeal Lymphoid Tissue |
| *Pseudomonas aeruginosa* | *peptidoglycan-associated lipoprotein* | ATGGAAATGCTGAAATTCGGC | CTTCTTCAGCTCGACGCGACG | SYBR Green | Designed in house |
| *Staphylococcus aureus* | *thermonuclease* | TTTGCGTATTGCCCTTTCG | GCTGGCATATGTATGGCAATTGT | SYBR Green | Designed in house |
| *Stenotrophomonas maltophilia* | *23S rRNA* | GCCGAAAGCCCAAGGTTT | CGACTTTCGTCCTCGCCTTA | SYBR Green | Development of a new real-time PCR system for simultaneous detection of bacteria and fungi in pathological samples |
| *Streptococcus pneumoniae* | *ply* | GCTTATGGGCGCCAAGTCTA | CAAAGCTTCAAAAGCAGCCTCTA | [ROX]CTCAAGTTGGAAACCACGAGTAAGAGTGATGAA[BHQ2] | Standardisation and evaluation of a quantitative multiplex real-time PCR assay for the rapid identification of Streptococcus pneumoniae |

Supplemental Table E2. Gram-stain and oxygen requirement for the common pathogens identified by rapid metagenomics.

| species | Gram stain | Oxygen requirement |
| --- | --- | --- |
| *Achromobacter xylosoxidans* | Gram- | aerobe |
| *Acinetobacter baumannii* | Gram- | aerobe |
| *Acinetobacter pittii* | Gram- | aerobe |
| *Burkholderia cepacia* | Gram- | aerobe |
| *Burkholderia multivorans* | Gram- | aerobe |
| *Corynebacterium striatum* | Gram+ | aerobe |
| *Enterobacter aerogenes* | Gram- | facultative |
| *Enterobacter cloacae* | Gram- | facultative |
| *Escherichia coli* | Gram- | facultative |
| *Haemophilus influenzae* | Gram- | facultative |
| *Klebsiella oxytoca* | Gram- | facultative |
| *Klebsiella pneumoniae* | Gram- | facultative |
| *Moraxella catarrhalis* | Gram- | aerobe |
| *Pseudomonas aeruginosa* | Gram- | aerobe |
| *Staphylococcus aureus* | Gram+ | facultative |
| *Stenotrophomonas maltophilia* | Gram- | aerobe |
| *Streptococcus pneumoniae* | Gram+ | facultative |

Note:

Gram+,Gram-stain positive; Gram-, Gram-stain negative.

Supplemental Figure E1. Procedure of Rapid Nanopore-based Clinical Metagenomics and its Evaluation

A. Workflow of rapid nanopore-based clinical metagenomics. a. samples were collected from patients and processed with sample preparation, including host depletion and DNA extraction. Sequencing library were built after samples were fully prepared. Sequencing was preformed using ONT’s GridION platform and real-time analysis was executed. The identified microbes are denoted as Meta-IDs. b. Time point at each step of the workflow. B. Evaluation pipeline. Meta-IDs were first compared with pre-defined pathogens (25 kinds of pathogens either from the culture results or from the experimental validation results), then compared with the experimental verification results, and at last went through clinical review.

Supplemental Figure E2. Heatmap of PPV, NPV, sensitivity and specificity using different thresholds in Meta-ID definition. X-axis is the thresholds for abundance, i.e., species with abundance over certain value are considered as the Meta-ID; y-axis is the thresholds for top N rank, i.e., top N most abundant species are considered as the Meta-ID. PPV is abbreviation for positive predictive value, SEN is abbreviation for sensitivity, SPE is abbreviation for specificity and NPV is abbreviation for negative predictive value.

Supplemental Figure E3. Comparison between routine clinical microbiology tests and rapid nanopore-based metagenomics

Culture results were annotated as positive or negative, by the results of routine culture and urinary antigen testing. true positive (TP), false positive (FP), true negative (TN) and false negative (FN) were defined as shown above.

Supplemental Figure E4. Comparison of turn-around time for rapid metagenomics and routine culture methods. Green dots represent the turn-around time of rapid metagenomics and red dots represent the turn-around time of culture method. Turn-around time is the time from sample to result reporting.

Supplemental Figure E5. Simulation of how reads length and error rate affect microbe alignment. Four species’ reference genomes (*Mycobacterium tuberculosis*: GCF_000195955.2, *Escherichia coli*: GCF_000008865.2, *Pseudomonas aeruginosa*: GCF_000006765.1 and *Shigella flexneri:* GCF_000006925.2) were used to simulate nanopore sequencing reads by DeepSimulator.[5] Reads with different length and different error rate (error rate was control by different parameters of DeepSimulator) were aligned back to corresponding reference genome. If a read aligned back to its corresponding reference genome, it was properly aligned, otherwise, it was wrongly aligned. The distribution of properly- and wrongly-aligned reads were plotted in boxplot, where X-axis showed different simulation parameters that controls error rate and Y-axis showed reads length. Wrongly-aligned reads showed much shorter read length but not affected by different error rate.

Supplemental Figure E6. Clinical insights analysis. Pathogen identification rate of Culture method (blue) and culture method plus rapid metagenomics (red), when applied in different samples types (BALF and sputum), different wards ( general wards and ICU) and different diseases.


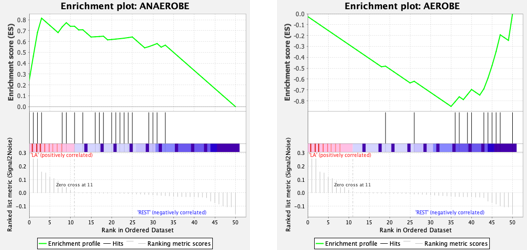


Supplemental Figure E7. Gene set enrichment analysis for anaerobic(left) and aerobic (right) species in patients with lung abscess. Only BALF samples were used.

**References:**

1 Charalampous T, Kay GL, Richardson H, *et al.* Nanopore metagenomics enables rapid clinical diagnosis of bacterial lower respiratory infection. *Nat Biotechnol* 2019;**37**:783–92. doi:10.1038/s41587-019-0156-5

2 Salter SJ, Cox MJ, Turek EM, *et al.* Reagent and laboratory contamination can critically impact sequence-based microbiome analyses. *BMC Biol* 2014;**12**:87.

3 Subramanian A, Tamayo P, Mootha VK, *et al.* Gene set enrichment analysis: a knowledge-based approach for interpreting genome-wide expression profiles. *Proc Natl Acad Sci U S A* 2005;**102**:15545–50. doi:10.1073/pnas.0506580102

4 Blauwkamp TA, Thair S, Rosen MJ, *et al.* Analytical and clinical validation of a microbial cell-free DNA sequencing test for infectious disease. *Nat Microbiol* 2019;**4**:663–74. doi:10.1038/s41564-018-0349-6

5 Li Y, Han R, Bi C, *et al.* DeepSimulator: a deep simulator for Nanopore sequencing. *Bioinforma Oxf Engl* 2018;**34**:2899–908. doi:10.1093/bioinformatics/bty223
