# Supplementary material for: Prospective Evaluation of a Rapid Clinical Metagenomics Test for Bacterial Pneumonia
Source: Front Cell Infect Microbiol. 2021 Oct 19;11:684965. doi: 10.3389/fcimb.2021.684965 (PMC8560692; doi:10.3389/fcimb.2021.684965)
Supplement: Supplementary file 2 [file DataSheet_2.docx]

**Diagnostic criteria**

**Ⅰ. CAP**

1. Acute infection of the pulmonary parenchyma acquired at community；
2. a: Cough with sputum production or change in color of respiratory secretions with or without chest discomfort, dyspnea or hemoptysis;

b: Fever;

c: Signs of lung consolidation and auscultatory findings such as altered breath sounds and/or localized rales;

d: WBC > 10×10^9^/L or < 4×10^9^/L;

1. An acute infiltration on a chest radio graph with or without pleural effusion;

If meet 1, 3 and any of 2, and exclude tuberculosis, lung abscess, interstitial lung disease et al, we could establish the diagnosis.^1,2^

**Ⅱ. HAP**

1. Patients with pneumonia that occurs 48 hours or more after admission and did not appear to be incubating at the time of admission;
2. Patient has a radiographic infiltrate that is new or progressive, along with clinical findings suggesting infection, which include the onset of fever, purulent sputum, leukocytosis, and decline in oxygenation.^3^

**Ⅲ. AECOPD**

Patients with COPD develop acute exacerbations of respiratory symptoms (dyspnoea, cough and/or sputum beyond day-to-day variability) and need to change drug therapy. Patients with Anthonisen Ⅰ and Anthonisen Ⅱ were enrolled in study.^4,5^

**Ⅳ. AEBX**

Patients with bronchiectasis develop acute exacerbations of respiratory symptoms (increasing sputum volume or purulence, worsening dyspnea, increased cough, declining lung function, increased fatigue/malaise) or the appearance of new symptoms (fever, pleurisy, haemoptysis, requirement for antibiotic treatment).^6^

**Ⅴ. CAP-ICH**

Patients with CAP meet following conditions should be considered immunocompromised, such as HIV infection, hematological cancer, chemotherapy during the last 3 months, biological drug use, lung transplantation, long term steroid use, lung cancer with either neutropenia or chemotherapy, other solid tumor with either neutropenia or chemotherapy.^7^

**Ⅵ. Lung abscess**

According to the history of oral surgery, disturbance states of consciousness, frequent vomiting, aspiration, fever with shivering, cough, and the significant increase in the total number of white blood cells and neutrophils, and chest radiography showing a lung cavity with an air-fluid level, and computed tomographic (CT) scans , ^8,9^the diagnosis can be made.

**Reference：**

1.Bartlett J. G., Dowell S. F., Mandell L. A., File T. M., Musher D. M., Fine M. J. Practice guidelines for the management of community-acquired pneumonia in adults. Infectious Diseases Society of America. *Clin Infect Dis Off Publ Infect Dis Soc Am* 2000;**31**(2):347–82. Doi: 10.1086/313954.

2.Liu You-ning, Cao Bin, Wang Hui, Chen Liang-an, She Dan-yang, Zhao Tie-mei, et al. [Adult hospital acquired pneumonia: a multicenter study on microbiology and clinical characteristics of patients from 9 Chinese cities]. *Zhonghua Jie He He Hu Xi Za Zhi Zhonghua Jiehe He Huxi Zazhi Chin J Tuberc Respir Dis* 2012;**35**(10):739–46.

3.American Thoracic Society, Infectious Diseases Society of America. Guidelines for the management of adults with hospital-acquired, ventilator-associated, and healthcare-associated pneumonia. *Am J Respir Crit Care Med* 2005;**171**(4):388–416. Doi: 10.1164/rccm.200405-644ST.

4.Woodhead M., Blasi F., Ewig S., Garau J., Huchon G., Ieven M., et al. Guidelines for the management of adult lower respiratory tract infections--full version. *Clin Microbiol Infect Off Publ Eur Soc Clin Microbiol Infect Dis* 2011;**17 Suppl 6**:E1-59. Doi: 10.1111/j.1469-0691.2011.03672.x.

5.Anthonisen N. R., Manfreda J., Warren C. P., Hershfield E. S., Harding G. K., Nelson N. A. Antibiotic therapy in exacerbations of chronic obstructive pulmonary disease. *Ann Intern Med* 1987;**106**(2):196–204. Doi: 10.7326/0003-4819-106-2-196.

6.Pasteur M. C., Bilton D., Hill A. T., British Thoracic Society Bronchiectasis non-CF Guideline Group. British Thoracic Society guideline for non-CF bronchiectasis. *Thorax* 2010;**65 Suppl 1**:i1-58. Doi: 10.1136/thx.2010.136119.

7.Di Pasquale Marta Francesca, Sotgiu Giovanni, Gramegna Andrea, Radovanovic Dejan, Terraneo Silvia, Reyes Luis F., et al. Prevalence and Etiology of Community-acquired Pneumonia in Immunocompromised Patients. *Clin Infect Dis Off Publ Infect Dis Soc Am* 2019;**68**(9):1482–93. Doi: 10.1093/cid/ciy723.

8.Stark D. D., Federle M. P., Goodman P. C., Podrasky A. E., Webb W. R. Differentiating lung abscess and empyema: radiography and computed tomography. *AJR Am J Roentgenol* 1983;**141**(1):163–7. Doi: 10.2214/ajr.141.1.163.

9.Williford ME, Godwin JD. Computed tomography of lung abscess and empyema. *Radiol Clin North Am* 1983;**21**(3):575–583.
