# Supplementary material for: Prospective Evaluation of a Rapid Clinical Metagenomics Test for Bacterial Pneumonia
Source: Front Cell Infect Microbiol. 2021 Oct 19;11:684965. doi: 10.3389/fcimb.2021.684965 (PMC8560692; doi:10.3389/fcimb.2021.684965)
Supplement: Supplementary file 3 [file DataSheet_3.docx]

| Study ID | Sample type | Culture Results | Meta-IDs | Abundance | qPCR or Sanger Verified Species | qPCR or Sanger Refuted Species |
| --- | --- | --- | --- | --- | --- | --- |
| L2 | Sputum |  | Veillonella_parvula | 26.2% |  | Streptococcus_pneumoniae;Enterococcus_faecalis;Stenotrophomonas_maltophilia;Pseudomonas_aeruginosa |
| L10 | Sputum |  | Rothia_mucilaginosa | 15.9% | Pseudomonas_aeruginosa | Enterococcus_faecalis;Enterococcus_faecium;Haemophilus_influenzae;Corynebacterium_striatum;Staphylococcus_aureus;Streptococcus_pneumoniae |
| L10 | Sputum |  | Streptococcus_mitis | 8.4% | Pseudomonas_aeruginosa | Enterococcus_faecalis;Enterococcus_faecium;Haemophilus_influenzae;Corynebacterium_striatum;Staphylococcus_aureus;Streptococcus_pneumoniae |
| L22 | BALF |  | Pseudomonas_aeruginosa | 68.8% | Pseudomonas_aeruginosa |  |
| L24 | BALF | Haemophilus_influenzae | Haemophilus_influenzae | 89.1% | Haemophilus_influenzae |  |
| M3 | BALF | Acinetobacter_baumannii;Klebsiella_pneumoniae | Acinetobacter_baumannii | 96.9% | Klebsiella_pneumoniae;Enterococcus_faecium;Corynebacterium_striatum;Acinetobacter_baumannii | Acinetobacter_pittii;Pseudomonas_aeruginosa;Escherichia_coli |
| M3 | BALF | Acinetobacter_baumannii;Klebsiella_pneumoniae | Klebsiella_pneumoniae | 2.6% | Klebsiella_pneumoniae;Enterococcus_faecium;Corynebacterium_striatum;Acinetobacter_baumannii | Acinetobacter_pittii;Pseudomonas_aeruginosa;Escherichia_coli |
| M7 | BALF |  | Pseudomonas_aeruginosa | 50.0% |  | Acinetobacter_baumannii;Pseudomonas_aeruginosa |
| M8 | BALF |  | Pseudomonas_aeruginosa | 63.0% | Pseudomonas_aeruginosa | Acinetobacter_baumannii;Enterococcus_faecium |
| M9 | BALF | Enterobacter_cloacae | Bacteroides_cellulosilyticus | 5.9% | Enterococcus_faecium;Corynebacterium_striatum;Escherichia_coli;Streptococcus_pneumoniae | Klebsiella_pneumoniae;Acinetobacter_baumannii;Staphylococcus_aureus;Pseudomonas_aeruginosa;Enterococcus_faecalis;Enterobacter_aerogenes;Klebsiella_oxytoca |
| M9 | BALF | Enterobacter_cloacae | Corynebacterium_striatum | 4.4% | Enterococcus_faecium;Corynebacterium_striatum;Escherichia_coli;Streptococcus_pneumoniae | Klebsiella_pneumoniae;Acinetobacter_baumannii;Staphylococcus_aureus;Pseudomonas_aeruginosa;Enterococcus_faecalis;Enterobacter_aerogenes;Klebsiella_oxytoca |
| M9 | BALF | Enterobacter_cloacae | Enterococcus_faecium | 70.2% | Enterococcus_faecium;Corynebacterium_striatum;Escherichia_coli;Streptococcus_pneumoniae | Klebsiella_pneumoniae;Acinetobacter_baumannii;Staphylococcus_aureus;Pseudomonas_aeruginosa;Enterococcus_faecalis;Enterobacter_aerogenes;Klebsiella_oxytoca |
| N1 | BALF | Streptococcus_pneumoniae | Rothia_mucilaginosa | 21.5% | Streptococcus_pneumoniae;Pseudomonas_aeruginosa | Enterococcus_faecium;Enterococcus_faecalis;Acinetobacter_baumannii |
| N1 | BALF | Streptococcus_pneumoniae | Streptococcus_pneumoniae | 10.8% | Streptococcus_pneumoniae;Pseudomonas_aeruginosa | Enterococcus_faecium;Enterococcus_faecalis;Acinetobacter_baumannii |
| N8 | BALF | Escherichia_coli | Pseudomonas_aeruginosa | 68.2% |  | Pseudomonas_aeruginosa;Escherichia_coli |
| O3 | BALF | Acinetobacter_baumannii | Acinetobacter_baumannii | 71.2% | Enterococcus_faecium | Acinetobacter_baumannii;Pseudomonas_aeruginosa |
| O3 | BALF | Acinetobacter_baumannii | Enterococcus_faecium | 16.2% | Enterococcus_faecium | Acinetobacter_baumannii;Pseudomonas_aeruginosa |
| O5 | Sputum | Pseudomonas_aeruginosa | Corynebacterium_striatum | 11.9% | Corynebacterium_striatum;Achromobacter_xylosoxidans;Stenotrophomonas_maltophilia |  |
| O5 | Sputum | Pseudomonas_aeruginosa | Pseudomonas_aeruginosa | 39.1% | Corynebacterium_striatum;Achromobacter_xylosoxidans;Stenotrophomonas_maltophilia |  |
| O5 | Sputum | Pseudomonas_aeruginosa | Stenotrophomonas_maltophilia | 13.6% | Corynebacterium_striatum;Achromobacter_xylosoxidans;Stenotrophomonas_maltophilia |  |
| O6 | Sputum | Klebsiella_pneumoniae | Klebsiella_pneumoniae | 11.9% | Klebsiella_pneumoniae;Pseudomonas_aeruginosa |  |
| O6 | Sputum | Klebsiella_pneumoniae | Pseudomonas_aeruginosa | 8.3% | Klebsiella_pneumoniae;Pseudomonas_aeruginosa |  |
| O6 | Sputum | Klebsiella_pneumoniae | Rhodococcus_qingshengii | 10.1% | Klebsiella_pneumoniae;Pseudomonas_aeruginosa |  |
| O7 | Sputum | Pseudomonas_aeruginosa;Stenotrophomonas_maltophilia | Burkholderia_cepacia | 14.9% | Corynebacterium_striatum;Achromobacter_xylosoxidans;Stenotrophomonas_maltophilia;Burkholderia_cepacia |  |
| O7 | Sputum | Pseudomonas_aeruginosa;Stenotrophomonas_maltophilia | Corynebacterium_striatum | 31.8% | Corynebacterium_striatum;Achromobacter_xylosoxidans;Stenotrophomonas_maltophilia;Burkholderia_cepacia |  |
| O7 | Sputum | Pseudomonas_aeruginosa;Stenotrophomonas_maltophilia | Pseudomonas_aeruginosa | 29.4% | Corynebacterium_striatum;Achromobacter_xylosoxidans;Stenotrophomonas_maltophilia;Burkholderia_cepacia |  |
| O7 | Sputum | Pseudomonas_aeruginosa;Stenotrophomonas_maltophilia | Stenotrophomonas_maltophilia | 12.4% | Corynebacterium_striatum;Achromobacter_xylosoxidans;Stenotrophomonas_maltophilia;Burkholderia_cepacia |  |
| O8 | Sputum |  | Rhodococcus_qingshengii | 11.4% | Pseudomonas_aeruginosa | Streptococcus_pneumoniae;Acinetobacter_baumannii |
| O8 | Sputum |  | Streptococcus_mitis | 25.0% | Pseudomonas_aeruginosa | Streptococcus_pneumoniae;Acinetobacter_baumannii |
| P2 | BALF | Pseudomonas_aeruginosa | Parvimonas_micra | 15.9% | Enterococcus_faecium | Acinetobacter_baumannii;Klebsiella_pneumoniae;Streptococcus_pneumoniae |
| P2 | BALF | Pseudomonas_aeruginosa | Pseudomonas_aeruginosa | 24.3% | Enterococcus_faecium | Acinetobacter_baumannii;Klebsiella_pneumoniae;Streptococcus_pneumoniae |
| P2 | BALF | Pseudomonas_aeruginosa | Streptococcus_mitis | 26.2% | Enterococcus_faecium | Acinetobacter_baumannii;Klebsiella_pneumoniae;Streptococcus_pneumoniae |
| P2 | BALF | Pseudomonas_aeruginosa | Streptococcus_pneumoniae | 5.6% | Enterococcus_faecium | Acinetobacter_baumannii;Klebsiella_pneumoniae;Streptococcus_pneumoniae |
| P5 | BALF |  | Corynebacterium_striatum | 23.1% | Enterococcus_faecium | Klebsiella_pneumoniae;Acinetobacter_pittii;Corynebacterium_striatum;Pseudomonas_aeruginosa |
| P5 | BALF |  | Enterococcus_faecium | 12.8% | Enterococcus_faecium | Klebsiella_pneumoniae;Acinetobacter_pittii;Corynebacterium_striatum;Pseudomonas_aeruginosa |
| P7 | BALF | Klebsiella_pneumoniae;Acinetobacter_baumannii | Acinetobacter_baumannii | 85.0% | Klebsiella_pneumoniae;Corynebacterium_striatum | Acinetobacter_pittii;Stenotrophomonas_maltophilia;Pseudomonas_aeruginosa;Achromobacter_xylosoxidans;Escherichia_coli;Streptococcus_pneumoniae |
| P7 | BALF | Klebsiella_pneumoniae;Acinetobacter_baumannii | Klebsiella_pneumoniae | 14.0% | Klebsiella_pneumoniae;Corynebacterium_striatum | Acinetobacter_pittii;Stenotrophomonas_maltophilia;Pseudomonas_aeruginosa;Achromobacter_xylosoxidans;Escherichia_coli;Streptococcus_pneumoniae |
| P8 | Sputum |  | Prevotella_jejuni | 10.9% | Klebsiella_pneumoniae;Pseudomonas_aeruginosa | Streptococcus_pneumoniae;Enterococcus_faecalis;Stenotrophomonas_maltophilia;Corynebacterium_striatum;Enterococcus_faecium;Staphylococcus_aureus;Acinetobacter_baumannii |
| P8 | Sputum |  | Streptococcus_parasanguinis | 31.6% | Klebsiella_pneumoniae;Pseudomonas_aeruginosa | Streptococcus_pneumoniae;Enterococcus_faecalis;Stenotrophomonas_maltophilia;Corynebacterium_striatum;Enterococcus_faecium;Staphylococcus_aureus;Acinetobacter_baumannii |
| P8 | Sputum |  | Veillonella_atypica | 24.6% | Klebsiella_pneumoniae;Pseudomonas_aeruginosa | Streptococcus_pneumoniae;Enterococcus_faecalis;Stenotrophomonas_maltophilia;Corynebacterium_striatum;Enterococcus_faecium;Staphylococcus_aureus;Acinetobacter_baumannii |
| P9 | Sputum |  | Bacteroides_heparinolyticus | 28.6% |  | Klebsiella_pneumoniae;Streptococcus_pneumoniae |
| P9 | Sputum |  | Porphyromonas_gingivalis | 57.6% |  | Klebsiella_pneumoniae;Streptococcus_pneumoniae |
| P9 | Sputum |  | Prevotella_intermedia | 3.5% |  | Klebsiella_pneumoniae;Streptococcus_pneumoniae |
| P10 | Sputum |  | Pseudomonas_aeruginosa | 27.2% |  | Klebsiella_pneumoniae;Staphylococcus_aureus;Enterococcus_faecalis;Enterococcus_faecium;Haemophilus_influenzae;Stenotrophomonas_maltophilia |
| P10 | Sputum |  | Veillonella_parvula | 17.7% |  | Klebsiella_pneumoniae;Staphylococcus_aureus;Enterococcus_faecalis;Enterococcus_faecium;Haemophilus_influenzae;Stenotrophomonas_maltophilia |
| Q5 | Sputum |  | Lactobacillus_paracasei | 20.0% | Pseudomonas_aeruginosa | Moraxella_catarrhalis;Streptococcus_pneumoniae;Corynebacterium_striatum |
| Q5 | Sputum |  | Pseudomonas_aeruginosa | 5.2% | Pseudomonas_aeruginosa | Moraxella_catarrhalis;Streptococcus_pneumoniae;Corynebacterium_striatum |
| Q5 | Sputum |  | Rothia_mucilaginosa | 33.0% | Pseudomonas_aeruginosa | Moraxella_catarrhalis;Streptococcus_pneumoniae;Corynebacterium_striatum |
| Q9 | Sputum |  | Streptococcus_mitis | 56.1% |  | Enterococcus_faecalis;Streptococcus_pneumoniae;Klebsiella_pneumoniae;Staphylococcus_aureus;Stenotrophomonas_maltophilia;Moraxella_catarrhalis |
| Q9 | Sputum |  | Streptococcus_pneumoniae | 11.3% |  | Enterococcus_faecalis;Streptococcus_pneumoniae;Klebsiella_pneumoniae;Staphylococcus_aureus;Stenotrophomonas_maltophilia;Moraxella_catarrhalis |
| R5 | Sputum |  | Corynebacterium_striatum | 17.4% | Staphylococcus_aureus;Enterococcus_faecium;Corynebacterium_striatum | Streptococcus_pneumoniae;Enterococcus_faecalis;Stenotrophomonas_maltophilia;Pseudomonas_aeruginosa; |
| R5 | Sputum |  | Staphylococcus_aureus | 4.5% | Staphylococcus_aureus;Enterococcus_faecium;Corynebacterium_striatum | Streptococcus_pneumoniae;Enterococcus_faecalis;Stenotrophomonas_maltophilia;Pseudomonas_aeruginosa; |
| R5 | Sputum |  | Streptococcus_oralis | 15.2% | Staphylococcus_aureus;Enterococcus_faecium;Corynebacterium_striatum | Streptococcus_pneumoniae;Enterococcus_faecalis;Stenotrophomonas_maltophilia;Pseudomonas_aeruginosa; |
| R6 | Sputum |  | Enterococcus_faecium | 3.1% | Enterococcus_faecium | Streptococcus_pneumoniae;Pseudomonas_aeruginosa |
| R6 | Sputum |  | Lactobacillus_pentosus | 14.2% | Enterococcus_faecium | Streptococcus_pneumoniae;Pseudomonas_aeruginosa |
| R6 | Sputum |  | Rothia_mucilaginosa | 27.2% | Enterococcus_faecium | Streptococcus_pneumoniae;Pseudomonas_aeruginosa |
| R6 | Sputum |  | Streptococcus_pneumoniae | 1.7% | Enterococcus_faecium | Streptococcus_pneumoniae;Pseudomonas_aeruginosa |
| R6 | Sputum |  | Veillonella_parvula | 14.0% | Enterococcus_faecium | Streptococcus_pneumoniae;Pseudomonas_aeruginosa |
| S2 | BALF |  | Veillonella_parvula | 11.6% |  | Stenotrophomonas_maltophilia;Haemophilus_influenzae;Pseudomonas_aeruginosa;Corynebacterium_striatum |
| S3 | Sputum |  | Fusobacterium_necrophorum | 13.2% |  |  |
| S3 | Sputum |  | Fusobacterium_nucleatum | 26.3% |  |  |
| S3 | Sputum |  | Parvimonas_micra | 30.9% |  |  |
| S4 | Sputum |  | Rothia_mucilaginosa | 67.0% |  | Corynebacterium_striatum;Streptococcus_pneumoniae;Pseudomonas_aeruginosa;Enterococcus_faecalis;Achromobacter_xylosoxidans;Stenotrophomonas_maltophilia |
| S4 | Sputum |  | Veillonella_parvula | 4.4% |  | Corynebacterium_striatum;Streptococcus_pneumoniae;Pseudomonas_aeruginosa;Enterococcus_faecalis;Achromobacter_xylosoxidans;Stenotrophomonas_maltophilia |
| S11 | Sputum |  | Haemophilus_influenzae | 29.4% | Haemophilus_influenzae;Pseudomonas_aeruginosa | Corynebacterium_striatum;Acinetobacter_baumannii |
| S11 | Sputum |  | Lactobacillus_rhamnosus | 15.1% | Haemophilus_influenzae;Pseudomonas_aeruginosa | Corynebacterium_striatum;Acinetobacter_baumannii |
| T3 | BALF |  | Chlamydia_psittaci | 94.8% |  | Acinetobacter_pittii;Corynebacterium_striatum;Acinetobacter_baumannii |
| T4 | BALF |  | Pseudomonas_aeruginosa | 64.7% |  | Streptococcus_pneumoniae;Stenotrophomonas_maltophilia;Pseudomonas_aeruginosa |
| T8 | BALF |  | Streptococcus_mitis | 10.1% |  | Enterococcus_faecium;Streptococcus_pneumoniae;Haemophilus_influenzae;Enterococcus_faecalis;Pseudomonas_aeruginosa;Corynebacterium_striatum |
| T8 | BALF |  | Streptococcus_salivarius | 10.5% |  | Enterococcus_faecium;Streptococcus_pneumoniae;Haemophilus_influenzae;Enterococcus_faecalis;Pseudomonas_aeruginosa;Corynebacterium_striatum |
| T8 | BALF |  | Veillonella_atypica | 8.4% |  | Enterococcus_faecium;Streptococcus_pneumoniae;Haemophilus_influenzae;Enterococcus_faecalis;Pseudomonas_aeruginosa;Corynebacterium_striatum |
| V6 | Sputum | Pseudomonas_aeruginosa | Corynebacterium_striatum | 68.1% | Achromobacter_xylosoxidans;Burkholderia_cepacia;Stenotrophomonas_maltophilia;Corynebacterium_striatum | Haemophilus_influenzae;Klebsiella_pneumoniae |
| V6 | Sputum | Pseudomonas_aeruginosa | Pseudomonas_aeruginosa | 7.1% | Achromobacter_xylosoxidans;Burkholderia_cepacia;Stenotrophomonas_maltophilia;Corynebacterium_striatum | Haemophilus_influenzae;Klebsiella_pneumoniae |
| T10 | BALF | Acinetobacter_baumannii | Acinetobacter_baumannii | 99.7% |  |  |
| T16 | Sputum |  | Haemophilus_influenzae | 56.0% | Haemophilus_influenzae |  |
| T16 | Sputum |  | Pseudomonas_aeruginosa | 32.7% | Haemophilus_influenzae |  |
| T16 | Sputum |  | Rothia_mucilaginosa | 5.7% | Haemophilus_influenzae |  |
| W3 | BALF | Staphylococcus_aureus | Pseudomonas_aeruginosa | 27.3% | Pseudomonas_aeruginosa | Corynebacterium_striatum;Stenotrophomonas_maltophilia |
| W3 | BALF | Staphylococcus_aureus | Staphylococcus_aureus | 26.0% | Pseudomonas_aeruginosa | Corynebacterium_striatum;Stenotrophomonas_maltophilia |
| Y2 | BALF | Klebsiella_pneumoniae | Klebsiella_pneumoniae | 75.8% |  | Klebsiella_pneumoniae;Escherichia_coli;Enterobacter_aerogenes;Klebsiella_oxytoca |
| Y13 | Sputum |  | Corynebacterium_striatum | 95.0% | Corynebacterium_striatum | Streptococcus_pneumoniae;Pseudomonas_aeruginosa;Stenotrophomonas_maltophilia |
| Z1 | BALF |  | Pseudomonas_aeruginosa | 66.7% |  | Pseudomonas_aeruginosa |
| Z5 | BALF | Pseudomonas_aeruginosa | Pseudomonas_aeruginosa | 95.6% | Pseudomonas_aeruginosa | Stenotrophomonas_maltophilia |
| Z7 | BALF |  | Prevotella_jejuni | 42.7% | Staphylococcus_aureus;Pseudomonas_aeruginosa | Streptococcus_pneumoniae |
| Z7 | BALF |  | Rothia_mucilaginosa | 19.2% | Staphylococcus_aureus;Pseudomonas_aeruginosa | Streptococcus_pneumoniae |
| Z7 | BALF |  | Staphylococcus_aureus | 6.1% | Staphylococcus_aureus;Pseudomonas_aeruginosa | Streptococcus_pneumoniae |
| AB3 | BALF | Klebsiella_pneumoniae | Bacteroides_fragilis | 48.2% |  | Klebsiella_pneumoniae;Burkholderia_cepacia;Acinetobacter_baumannii |
| AB3 | BALF | Klebsiella_pneumoniae | Parvimonas_micra | 12.6% |  | Klebsiella_pneumoniae;Burkholderia_cepacia;Acinetobacter_baumannii |
| AB8 | Sputum |  | Filifactor_alocis | 4.3% | Streptococcus_pneumoniae;Haemophilus_influenzae;Pseudomonas_aeruginosa | Enterococcus_faecalis |
| AB8 | Sputum |  | Porphyromonas_gingivalis | 51.9% | Streptococcus_pneumoniae;Haemophilus_influenzae;Pseudomonas_aeruginosa | Enterococcus_faecalis |
| AB8 | Sputum |  | Tannerella_forsythia | 32.2% | Streptococcus_pneumoniae;Haemophilus_influenzae;Pseudomonas_aeruginosa | Enterococcus_faecalis |
| AC1 | BALF |  | Pseudomonas_aeruginosa | 84.0% | Pseudomonas_aeruginosa | Stenotrophomonas_maltophilia;Achromobacter_xylosoxidans;Enterococcus_faecium;Corynebacterium_striatum |
| AC11 | Sputum | Pseudomonas_aeruginosa;Stenotrophomonas_maltophilia | Corynebacterium_striatum | 20.8% | Stenotrophomonas_maltophilia;Corynebacterium_striatum;Achromobacter_xylosoxidans;Klebsiella_pneumoniae;Burkholderia_cepacia | Enterococcus_faecium;Escherichia_coli |
| AC11 | Sputum | Pseudomonas_aeruginosa;Stenotrophomonas_maltophilia | Pseudomonas_aeruginosa | 52.3% | Stenotrophomonas_maltophilia;Corynebacterium_striatum;Achromobacter_xylosoxidans;Klebsiella_pneumoniae;Burkholderia_cepacia | Enterococcus_faecium;Escherichia_coli |
| AC11 | Sputum | Pseudomonas_aeruginosa;Stenotrophomonas_maltophilia | Stenotrophomonas_maltophilia | 14.7% | Stenotrophomonas_maltophilia;Corynebacterium_striatum;Achromobacter_xylosoxidans;Klebsiella_pneumoniae;Burkholderia_cepacia | Enterococcus_faecium;Escherichia_coli |
| AD6 | Sputum |  | Haemophilus_influenzae | 64.4% | Haemophilus_influenzae | Stenotrophomonas_maltophilia;Corynebacterium_striatum;Pseudomonas_aeruginosa;Enterococcus_faecium |
| AD6 | Sputum |  | Pseudomonas_aeruginosa | 16.4% | Haemophilus_influenzae | Stenotrophomonas_maltophilia;Corynebacterium_striatum;Pseudomonas_aeruginosa;Enterococcus_faecium |
| AD7 | Sputum | Pseudomonas_aeruginosa | Pseudomonas_aeruginosa | 99.1% |  | Streptococcus_pneumoniae;Corynebacterium_striatum;Klebsiella_pneumoniae;Achromobacter_xylosoxidans;Enterococcus_faecalis;Stenotrophomonas_maltophilia;Burkholderia_cepacia |
| AD8 | Sputum |  | Rothia_mucilaginosa | 16.9% | Streptococcus_pneumoniae | Haemophilus_influenzae;Pseudomonas_aeruginosa;Corynebacterium_striatum;Enterococcus_faecium;Klebsiella_pneumoniae;Escherichia_coli;Stenotrophomonas_maltophilia;Staphylococcus_aureus;Acinetobacter_baumannii;Enterococcus_faecalis |
| AD8 | Sputum |  | Streptococcus_pneumoniae | 6.0% | Streptococcus_pneumoniae | Haemophilus_influenzae;Pseudomonas_aeruginosa;Corynebacterium_striatum;Enterococcus_faecium;Klebsiella_pneumoniae;Escherichia_coli;Stenotrophomonas_maltophilia;Staphylococcus_aureus;Acinetobacter_baumannii;Enterococcus_faecalis |
| AD8 | Sputum |  | Streptococcus_pseudopneumoniae | 12.1% | Streptococcus_pneumoniae | Haemophilus_influenzae;Pseudomonas_aeruginosa;Corynebacterium_striatum;Enterococcus_faecium;Klebsiella_pneumoniae;Escherichia_coli;Stenotrophomonas_maltophilia;Staphylococcus_aureus;Acinetobacter_baumannii;Enterococcus_faecalis |
| AE2 | BALF |  | Streptococcus_oralis | 43.0% | Enterococcus_faecium | Streptococcus_pneumoniae;Enterococcus_faecalis |
| AE6 | BALF |  | Pseudomonas_aeruginosa | 11.1% | Pseudomonas_aeruginosa | Streptococcus_pneumoniae;Haemophilus_influenzae; |
| AF8 | BALF | Pseudomonas_aeruginosa | Pseudomonas_aeruginosa | 88.3% | Pseudomonas_aeruginosa | Stenotrophomonas_maltophilia |
| AF17 | Sputum | Pseudomonas_aeruginosa | Pseudomonas_aeruginosa | 79.6% | Pseudomonas_aeruginosa |  |
| AG2 | BALF | Streptococcus_pneumoniae | Streptococcus_pneumoniae | 98.7% | Streptococcus_pneumoniae;Pseudomonas_aeruginosa | Stenotrophomonas_maltophilia;Enterococcus_faecalis;Enterococcus_faecium |
| AG6 | Sputum | Acinetobacter_baumannii | Prevotella_melaninogenica | 9.1% | Pseudomonas_aeruginosa | Streptococcus_pneumoniae;Acinetobacter_baumannii;Stenotrophomonas_maltophilia |
| AG6 | Sputum | Acinetobacter_baumannii | Rothia_mucilaginosa | 24.7% | Pseudomonas_aeruginosa | Streptococcus_pneumoniae;Acinetobacter_baumannii;Stenotrophomonas_maltophilia |
| AG6 | Sputum | Acinetobacter_baumannii | Veillonella_parvula | 19.6% | Pseudomonas_aeruginosa | Streptococcus_pneumoniae;Acinetobacter_baumannii;Stenotrophomonas_maltophilia |
| AI5 | BALF |  | Corynebacterium_striatum | 92.8% | Corynebacterium_striatum;Pseudomonas_aeruginosa | Enterococcus_faecalis |
| AI11 | Sputum |  | Streptococcus_mitis | 20.7% | Streptococcus_pneumoniae;Pseudomonas_aeruginosa | Enterococcus_faecium;Corynebacterium_striatum |
| AH9 | Sputum | Acinetobacter_pittii | Acinetobacter_pittii | 80.4% | Acinetobacter_pittii;Pseudomonas_aeruginosa | Acinetobacter_baumannii |
| AI14 | Sputum | Acinetobacter_baumannii | Pseudomonas_aeruginosa | 5.8% | Streptococcus_pneumoniae;Pseudomonas_aeruginosa |  |
| AI14 | Sputum | Acinetobacter_baumannii | Rothia_mucilaginosa | 65.6% | Streptococcus_pneumoniae;Pseudomonas_aeruginosa |  |
| AK2 | BALF |  | Pseudomonas_aeruginosa | 34.1% | Pseudomonas_aeruginosa | Acinetobacter_baumannii;Acinetobacter_pittii |
| AJ10 | Sputum | Acinetobacter_baumannii;Streptococcus_pneumoniae | Acinetobacter_baumannii | 100.0% | Enterococcus_faecium;Pseudomonas_aeruginosa;Acinetobacter_baumannii | Klebsiella_pneumoniae;Acinetobacter_pittii;Escherichia_coli;Enterobacter_aerogenes |
| AJ13 | Sputum | Acinetobacter_nosocomialis | Acinetobacter_baumannii | 38.5% | Staphylococcus_aureus;Corynebacterium_striatum;Pseudomonas_aeruginosa | Acinetobacter_pittii;Stenotrophomonas_maltophilia;Acinetobacter_baumannii |
| AJ13 | Sputum | Acinetobacter_nosocomialis | Staphylococcus_aureus | 42.9% | Staphylococcus_aureus;Corynebacterium_striatum;Pseudomonas_aeruginosa | Acinetobacter_pittii;Stenotrophomonas_maltophilia;Acinetobacter_baumannii |
| AK6 | Sputum |  | Pseudomonas_aeruginosa | 100.0% | Pseudomonas_aeruginosa |  |
| AJ12 | Sputum | Klebsiella_pneumoniae | Klebsiella_pneumoniae | 19.1% | Klebsiella_pneumoniae;Pseudomonas_aeruginosa | Streptococcus_pneumoniae |
| AJ12 | Sputum | Klebsiella_pneumoniae | Prevotella_melaninogenica | 16.9% | Klebsiella_pneumoniae;Pseudomonas_aeruginosa | Streptococcus_pneumoniae |
| AJ12 | Sputum | Klebsiella_pneumoniae | Veillonella_atypica | 18.0% | Klebsiella_pneumoniae;Pseudomonas_aeruginosa | Streptococcus_pneumoniae |
| AJ12 | Sputum | Klebsiella_pneumoniae | Veillonella_parvula | 15.7% | Klebsiella_pneumoniae;Pseudomonas_aeruginosa | Streptococcus_pneumoniae |
| AM5 | BALF | Pseudomonas_aeruginosa | Corynebacterium_striatum | 1.7% | Corynebacterium_striatum | Burkholderia_cepacia |
| AM5 | BALF | Pseudomonas_aeruginosa | Pseudomonas_aeruginosa | 95.3% | Corynebacterium_striatum | Burkholderia_cepacia |
| AL12 | Sputum | Burkholderia_cepacia | Burkholderia_cepacia | 99.5% |  |  |
| AN10 | Sputum |  | Filifactor_alocis | 7.9% | Haemophilus_influenzae;Streptococcus_pneumoniae;Pseudomonas_aeruginosa | Acinetobacter_baumannii;Enterococcus_faecalis;Enterococcus_faecium;Staphylococcus_aureus |
| AN10 | Sputum |  | Parvimonas_micra | 11.4% | Haemophilus_influenzae;Streptococcus_pneumoniae;Pseudomonas_aeruginosa | Acinetobacter_baumannii;Enterococcus_faecalis;Enterococcus_faecium;Staphylococcus_aureus |
| AN10 | Sputum |  | Streptococcus_constellatus | 12.8% | Haemophilus_influenzae;Streptococcus_pneumoniae;Pseudomonas_aeruginosa | Acinetobacter_baumannii;Enterococcus_faecalis;Enterococcus_faecium;Staphylococcus_aureus |
| AO3 | BALF | Pseudomonas_aeruginosa;Acinetobacter_baumannii | Acinetobacter_baumannii | 21.3% | Corynebacterium_striatum;Enterococcus_faecium;Burkholderia_cepacia | Acinetobacter_pittii;Enterococcus_faecalis;Escherichia_coli;Stenotrophomonas_maltophilia |
| AO3 | BALF | Pseudomonas_aeruginosa;Acinetobacter_baumannii | Corynebacterium_striatum | 18.8% | Corynebacterium_striatum;Enterococcus_faecium;Burkholderia_cepacia | Acinetobacter_pittii;Enterococcus_faecalis;Escherichia_coli;Stenotrophomonas_maltophilia |
| AO3 | BALF | Pseudomonas_aeruginosa;Acinetobacter_baumannii | Pseudomonas_aeruginosa | 58.9% | Corynebacterium_striatum;Enterococcus_faecium;Burkholderia_cepacia | Acinetobacter_pittii;Enterococcus_faecalis;Escherichia_coli;Stenotrophomonas_maltophilia |
| AO9 | Sputum | Burkholderia_cepacia | Burkholderia_cepacia | 99.2% | Pseudomonas_aeruginosa;Burkholderia_cepacia | Streptococcus_pneumoniae;Stenotrophomonas_maltophilia;Burkholderia_multivorans |
| AN11 | Sputum |  | Streptococcus_gordonii | 14.3% | Streptococcus_pneumoniae | Haemophilus_influenzae;Enterococcus_faecalis;Pseudomonas_aeruginosa;Corynebacterium_striatum;Enterococcus_faecium;Staphylococcus_aureus |
| AN11 | Sputum |  | Streptococcus_parasanguinis | 9.3% | Streptococcus_pneumoniae | Haemophilus_influenzae;Enterococcus_faecalis;Pseudomonas_aeruginosa;Corynebacterium_striatum;Enterococcus_faecium;Staphylococcus_aureus |
| AO2 | BALF |  | Pseudomonas_aeruginosa | 90.0% | Pseudomonas_aeruginosa | Haemophilus_influenzae;Burkholderia_cepacia |
| AN2 | BALF |  | Corynebacterium_striatum | 56.1% | Corynebacterium_striatum;Enterococcus_faecium;Pseudomonas_aeruginosa | Acinetobacter_baumannii;Stenotrophomonas_maltophilia |
| AN2 | BALF |  | Pseudomonas_aeruginosa | 29.3% | Corynebacterium_striatum;Enterococcus_faecium;Pseudomonas_aeruginosa | Acinetobacter_baumannii;Stenotrophomonas_maltophilia |
| AM8 | Sputum |  | Moraxella_catarrhalis | 87.9% | Moraxella_catarrhalis;Pseudomonas_aeruginosa | Acinetobacter_baumannii;Stenotrophomonas_maltophilia |
| AM8 | Sputum |  | Rothia_mucilaginosa | 9.4% | Moraxella_catarrhalis;Pseudomonas_aeruginosa | Acinetobacter_baumannii;Stenotrophomonas_maltophilia |
| AR7 | Sputum | Pseudomonas_aeruginosa | Pseudomonas_aeruginosa | 53.5% | Streptococcus_pneumoniae | Enterococcus_faecalis;Staphylococcus_aureus |
| AR7 | Sputum | Pseudomonas_aeruginosa | Streptococcus_mitis | 19.0% | Streptococcus_pneumoniae | Enterococcus_faecalis;Staphylococcus_aureus |
| AR7 | Sputum | Pseudomonas_aeruginosa | Streptococcus_parasanguinis | 6.4% | Streptococcus_pneumoniae | Enterococcus_faecalis;Staphylococcus_aureus |
| AR7 | Sputum | Pseudomonas_aeruginosa | Streptococcus_pneumoniae | 5.2% | Streptococcus_pneumoniae | Enterococcus_faecalis;Staphylococcus_aureus |
| AR8 | Sputum |  | Rothia_mucilaginosa | 45.6% |  | Streptococcus_pneumoniae;Pseudomonas_aeruginosa |
| AR8 | Sputum |  | Streptococcus_mitis | 8.1% |  | Streptococcus_pneumoniae;Pseudomonas_aeruginosa |
| AR12 | Sputum | Klebsiella_pneumoniae | Acinetobacter_baumannii | 76.8% | Klebsiella_pneumoniae;Enterococcus_faecium;Pseudomonas_aeruginosa;Acinetobacter_baumannii |  |
| AP3 | BALF |  | Acinetobacter_baumannii | 20.9% | Pseudomonas_aeruginosa | Acinetobacter_baumannii;Corynebacterium_striatum;Stenotrophomonas_maltophilia;Acinetobacter_baumannii |
| AP3 | BALF |  | Corynebacterium_striatum | 48.6% | Pseudomonas_aeruginosa | Acinetobacter_baumannii;Corynebacterium_striatum;Stenotrophomonas_maltophilia;Acinetobacter_baumannii |
| AP3 | BALF |  | Pseudomonas_aeruginosa | 11.5% | Pseudomonas_aeruginosa | Acinetobacter_baumannii;Corynebacterium_striatum;Stenotrophomonas_maltophilia;Acinetobacter_baumannii |
| AP3 | BALF |  | Stenotrophomonas_maltophilia | 14.2% | Pseudomonas_aeruginosa | Acinetobacter_baumannii;Corynebacterium_striatum;Stenotrophomonas_maltophilia;Acinetobacter_baumannii |
| AP4 | BALF | Klebsiella_pneumoniae | Klebsiella_pneumoniae | 99.0% | Klebsiella_pneumoniae;Pseudomonas_aeruginosa | Acinetobacter_baumannii;Corynebacterium_striatum |
| AR2 | BALF | Acinetobacter_baumannii;Pseudomonas_aeruginosa | Acinetobacter_baumannii | 18.1% | Corynebacterium_striatum;Stenotrophomonas_maltophilia | Klebsiella_pneumoniae;Achromobacter_xylosoxidans;Escherichia_coli;Burkholderia_cepacia;Enterobacter_aerogenes;Acinetobacter_baumannii;Acinetobacter_pittii |
| AR2 | BALF | Acinetobacter_baumannii;Pseudomonas_aeruginosa | Corynebacterium_striatum | 55.1% | Corynebacterium_striatum;Stenotrophomonas_maltophilia | Klebsiella_pneumoniae;Achromobacter_xylosoxidans;Escherichia_coli;Burkholderia_cepacia;Enterobacter_aerogenes;Acinetobacter_baumannii;Acinetobacter_pittii |
| AR2 | BALF | Acinetobacter_baumannii;Pseudomonas_aeruginosa | Pseudomonas_aeruginosa | 14.3% | Corynebacterium_striatum;Stenotrophomonas_maltophilia | Klebsiella_pneumoniae;Achromobacter_xylosoxidans;Escherichia_coli;Burkholderia_cepacia;Enterobacter_aerogenes;Acinetobacter_baumannii;Acinetobacter_pittii |
| AR2 | BALF | Acinetobacter_baumannii;Pseudomonas_aeruginosa | Stenotrophomonas_maltophilia | 10.6% | Corynebacterium_striatum;Stenotrophomonas_maltophilia | Klebsiella_pneumoniae;Achromobacter_xylosoxidans;Escherichia_coli;Burkholderia_cepacia;Enterobacter_aerogenes;Acinetobacter_baumannii;Acinetobacter_pittii |
| AR15 | Sputum | Staphylococcus_aureus | Staphylococcus_aureus | 99.5% | Staphylococcus_aureus | Stenotrophomonas_maltophilia;Pseudomonas_aeruginosa |
| AS1 | BALF | Pseudomonas_aeruginosa | Pseudomonas_aeruginosa | 99.8% | Pseudomonas_aeruginosa | Staphylococcus_aureus |
| AS3 | BALF | Moraxella_catarrhalis | Moraxella_catarrhalis | 97.4% | Moraxella_catarrhalis;Pseudomonas_aeruginosa | Staphylococcus_aureus |
| AS7 | BALF | Pseudomonas_aeruginosa | Haemophilus_influenzae | 4.5% | Haemophilus_influenzae;Streptococcus_pneumoniae | Corynebacterium_striatum;Enterococcus_faecalis;Klebsiella_pneumoniae;Stenotrophomonas_maltophilia;Staphylococcus_aureus |
| AS7 | BALF | Pseudomonas_aeruginosa | Pseudomonas_aeruginosa | 85.7% | Haemophilus_influenzae;Streptococcus_pneumoniae | Corynebacterium_striatum;Enterococcus_faecalis;Klebsiella_pneumoniae;Stenotrophomonas_maltophilia;Staphylococcus_aureus |
| AS7 | BALF | Pseudomonas_aeruginosa | Streptococcus_anginosus | 2.8% | Haemophilus_influenzae;Streptococcus_pneumoniae | Corynebacterium_striatum;Enterococcus_faecalis;Klebsiella_pneumoniae;Stenotrophomonas_maltophilia;Staphylococcus_aureus |
| AV2 | BALF | Streptococcus_pneumoniae | Streptococcus_pneumoniae | 96.8% | Streptococcus_pneumoniae | Klebsiella_pneumoniae |
| AV6 | BALF | Staphylococcus_aureus | Staphylococcus_aureus | 91.2% | Staphylococcus_aureus;Pseudomonas_aeruginosa | Klebsiella_pneumoniae;Acinetobacter_baumannii |
| AS8 | BALF | Enterobacter_cloacae | Capnocytophaga_leadbetteri | 5.1% | Streptococcus_pneumoniae;Pseudomonas_aeruginosa | Enterococcus_faecalis;Enterococcus_faecium;Klebsiella_pneumoniae;Acinetobacter_baumannii |
| AS8 | BALF | Enterobacter_cloacae | Enterobacter_cloacae | 3.2% | Streptococcus_pneumoniae;Pseudomonas_aeruginosa | Enterococcus_faecalis;Enterococcus_faecium;Klebsiella_pneumoniae;Acinetobacter_baumannii |
| AS8 | BALF | Enterobacter_cloacae | Enterococcus_faecalis | 25.0% | Streptococcus_pneumoniae;Pseudomonas_aeruginosa | Enterococcus_faecalis;Enterococcus_faecium;Klebsiella_pneumoniae;Acinetobacter_baumannii |
| AU2 | BALF | Acinetobacter_baumannii;Pseudomonas_aeruginosa | Acinetobacter_baumannii | 98.3% |  | Acinetobacter_pittii;Klebsiella_pneumoniae;Escherichia_coli;Stenotrophomonas_maltophilia;Acinetobacter_baumannii |
| AV8 | Sputum | Pseudomonas_aeruginosa | Pseudomonas_aeruginosa | 28.6% | Achromobacter_xylosoxidans;Corynebacterium_striatum | Enterococcus_faecalis;Klebsiella_pneumoniae;Escherichia_coli;Streptococcus_pneumoniae |
| AV8 | Sputum | Pseudomonas_aeruginosa | Serratia_marcescens | 32.1% | Achromobacter_xylosoxidans;Corynebacterium_striatum | Enterococcus_faecalis;Klebsiella_pneumoniae;Escherichia_coli;Streptococcus_pneumoniae |
| AV8 | Sputum | Pseudomonas_aeruginosa | Stenotrophomonas_maltophilia | 4.6% | Achromobacter_xylosoxidans;Corynebacterium_striatum | Enterococcus_faecalis;Klebsiella_pneumoniae;Escherichia_coli;Streptococcus_pneumoniae |
| AV8 | Sputum | Pseudomonas_aeruginosa | Streptococcus_oralis | 11.8% | Achromobacter_xylosoxidans;Corynebacterium_striatum | Enterococcus_faecalis;Klebsiella_pneumoniae;Escherichia_coli;Streptococcus_pneumoniae |
| AU5 | Sputum |  | Corynebacterium_striatum | 93.6% | Corynebacterium_striatum | Pseudomonas_aeruginosa;Stenotrophomonas_maltophilia |
| AU6 | Sputum |  | Prevotella_melaninogenica | 33.3% | Pseudomonas_aeruginosa | Corynebacterium_striatum |
| AV5 | BALF |  | Parvimonas_micra | 84.6% |  | Pseudomonas_aeruginosa |
| AW9 | Sputum |  | Actinomyces_pacaensis | 5.2% |  |  |
| AW9 | Sputum |  | Rothia_mucilaginosa | 64.6% |  |  |
| AX8 | BALF | Pseudomonas_aeruginosa | Pseudomonas_mendocina | 97.3% |  |  |
| AX12 | Sputum |  | Lactobacillus_crispatus | 11.0% |  |  |
| AX12 | Sputum |  | Parascardovia_denticolens | 20.7% |  |  |
| AX12 | Sputum |  | Veillonella_parvula | 8.7% |  |  |
| AY4 | BALF |  | Enterococcus_faecium | 29.0% | Enterococcus_faecium;Pseudomonas_aeruginosa |  |
| AY4 | BALF |  | Mycoplasma_pneumoniae | 65.2% | Enterococcus_faecium;Pseudomonas_aeruginosa |  |
| AY9 | Sputum |  | Bacteroides_heparinolyticus | 31.0% |  | Pseudomonas_aeruginosa |
| AY9 | Sputum |  | Parvimonas_micra | 48.3% |  | Pseudomonas_aeruginosa |
| AZ4 | BALF | Enterobacter_cloacae | Pseudomonas_aeruginosa | 15.2% |  | Streptococcus_pneumoniae;Pseudomonas_aeruginosa;Acinetobacter_baumannii |
| AZ4 | BALF | Enterobacter_cloacae | Rothia_mucilaginosa | 47.8% |  | Streptococcus_pneumoniae;Pseudomonas_aeruginosa;Acinetobacter_baumannii |
| AY7 | Sputum | Corynebacterium_striatum | Corynebacterium_striatum | 21.5% | Enterococcus_faecium;Corynebacterium_striatum | Pseudomonas_aeruginosa;Streptococcus_pneumoniae;Enterococcus_faecalis;Acinetobacter_baumannii |
| AY7 | Sputum | Corynebacterium_striatum | Rothia_mucilaginosa | 61.1% | Enterococcus_faecium;Corynebacterium_striatum | Pseudomonas_aeruginosa;Streptococcus_pneumoniae;Enterococcus_faecalis;Acinetobacter_baumannii |
| AX105 | Sputum |  | Streptococcus_pneumoniae | 64.3% | Streptococcus_pneumoniae;Haemophilus_influenzae;Pseudomonas_aeruginosa | Acinetobacter_baumannii |
| BA1 | BALF |  | Pseudomonas_aeruginosa | 88.9% | Pseudomonas_aeruginosa |  |
| BA6 | BALF |  | Haemophilus_influenzae | 98.1% | Haemophilus_influenzae | Acinetobacter_baumannii |
| BA10 | BALF | Acinetobacter_baumannii | Acinetobacter_baumannii | 96.8% | Enterococcus_faecium;Acinetobacter_baumannii | Klebsiella_pneumoniae;Acinetobacter_pittii;Pseudomonas_aeruginosa;Corynebacterium_striatum;Escherichia_coli;Streptococcus_pneumoniae;Staphylococcus_aureus |
| BA10 | BALF | Acinetobacter_baumannii | Lactobacillus_mucosae | 1.3% | Enterococcus_faecium;Acinetobacter_baumannii | Klebsiella_pneumoniae;Acinetobacter_pittii;Pseudomonas_aeruginosa;Corynebacterium_striatum;Escherichia_coli;Streptococcus_pneumoniae;Staphylococcus_aureus |
| BA11 | Sputum |  | Mycobacterium_tuberculosis | 91.4% |  | Streptococcus_pneumoniae;Pseudomonas_aeruginosa;Acinetobacter_baumannii |
| BA15 | Sputum |  | Corynebacterium_simulans | 84.7% |  | Corynebacterium_striatum;Acinetobacter_baumannii;Pseudomonas_aeruginosa;Staphylococcus_aureus;Streptococcus_pneumoniae |
| BA15 | Sputum |  | Rothia_mucilaginosa | 7.8% |  | Corynebacterium_striatum;Acinetobacter_baumannii;Pseudomonas_aeruginosa;Staphylococcus_aureus;Streptococcus_pneumoniae |
| BB11 | Sputum | Klebsiella_pneumoniae;Corynebacterium_striatum | Acinetobacter_baumannii | 30.3% | Staphylococcus_aureus;Corynebacterium_striatum;Streptococcus_pneumoniae;Pseudomonas_aeruginosa;Acinetobacter_baumannii |  |
| BB11 | Sputum | Klebsiella_pneumoniae;Corynebacterium_striatum | Corynebacterium_striatum | 54.8% | Staphylococcus_aureus;Corynebacterium_striatum;Streptococcus_pneumoniae;Pseudomonas_aeruginosa;Acinetobacter_baumannii |  |
| BB11 | Sputum | Klebsiella_pneumoniae;Corynebacterium_striatum | Lactobacillus_gasseri | 7.2% | Staphylococcus_aureus;Corynebacterium_striatum;Streptococcus_pneumoniae;Pseudomonas_aeruginosa;Acinetobacter_baumannii |  |
| BB12 | Sputum | Streptococcus_pneumoniae | Lactobacillus_rhamnosus | 24.5% | Streptococcus_pneumoniae |  |
| BB12 | Sputum | Streptococcus_pneumoniae | Rothia_dentocariosa | 10.6% | Streptococcus_pneumoniae |  |
| BB12 | Sputum | Streptococcus_pneumoniae | Streptococcus_parasanguinis | 11.0% | Streptococcus_pneumoniae |  |
| BB12 | Sputum | Streptococcus_pneumoniae | Streptococcus_pneumoniae | 12.3% | Streptococcus_pneumoniae |  |
| BB15 | Sputum |  | Rothia_mucilaginosa | 19.2% |  | Streptococcus_pneumoniae;Stenotrophomonas_maltophilia;Enterococcus_faecalis;Pseudomonas_aeruginosa;Corynebacterium_striatum |
| BB15 | Sputum |  | Streptococcus_parasanguinis | 16.8% |  | Streptococcus_pneumoniae;Stenotrophomonas_maltophilia;Enterococcus_faecalis;Pseudomonas_aeruginosa;Corynebacterium_striatum |
| BB15 | Sputum |  | Veillonella_parvula | 20.9% |  | Streptococcus_pneumoniae;Stenotrophomonas_maltophilia;Enterococcus_faecalis;Pseudomonas_aeruginosa;Corynebacterium_striatum |
| BB16 | Sputum | Moraxella_catarrhalis | Moraxella_catarrhalis | 18.4% | Streptococcus_pneumoniae;Moraxella_catarrhalis | Enterococcus_faecalis;Corynebacterium_striatum;Haemophilus_influenzae;Staphylococcus_aureus |
| BB16 | Sputum | Moraxella_catarrhalis | Streptococcus_parasanguinis | 20.3% | Streptococcus_pneumoniae;Moraxella_catarrhalis | Enterococcus_faecalis;Corynebacterium_striatum;Haemophilus_influenzae;Staphylococcus_aureus |
| BB16 | Sputum | Moraxella_catarrhalis | Veillonella_atypica | 13.0% | Streptococcus_pneumoniae;Moraxella_catarrhalis | Enterococcus_faecalis;Corynebacterium_striatum;Haemophilus_influenzae;Staphylococcus_aureus |
| BC8 | Sputum |  | Enterococcus_faecalis | 4.7% | Enterococcus_faecium;Enterococcus_faecalis;Pseudomonas_aeruginosa | Streptococcus_pneumoniae;Staphylococcus_aureus |
| BC8 | Sputum |  | Streptococcus_oralis | 45.0% | Enterococcus_faecium;Enterococcus_faecalis;Pseudomonas_aeruginosa | Streptococcus_pneumoniae;Staphylococcus_aureus |
| BD5 | BALF | Corynebacterium_striatum | Corynebacterium_striatum | 60.0% |  | Corynebacterium_striatum;Pseudomonas_aeruginosa |
| BE4 | BALF | Acinetobacter_baumannii | Acinetobacter_baumannii | 91.0% | Pseudomonas_aeruginosa | Achromobacter_xylosoxidans;Stenotrophomonas_maltophilia;Acinetobacter_baumannii |
| BE5 | Sputum | Pseudomonas_aeruginosa;Moraxella_catarrhalis | Moraxella_catarrhalis | 27.3% | Moraxella_catarrhalis | Achromobacter_xylosoxidans;Streptococcus_pneumoniae |
| BE5 | Sputum | Pseudomonas_aeruginosa;Moraxella_catarrhalis | Pseudomonas_aeruginosa | 69.1% | Moraxella_catarrhalis | Achromobacter_xylosoxidans;Streptococcus_pneumoniae |
| BE6 | Sputum | Pseudomonas_aeruginosa | Achromobacter_xylosoxidans | 85.3% | Achromobacter_xylosoxidans;Corynebacterium_striatum | Klebsiella_pneumoniae;Escherichia_coli;Streptococcus_pneumoniae;Moraxella_catarrhalis;Stenotrophomonas_maltophilia;Acinetobacter_baumannii;Burkholderia_cepacia |
| BE6 | Sputum | Pseudomonas_aeruginosa | Pseudomonas_aeruginosa | 12.3% | Achromobacter_xylosoxidans;Corynebacterium_striatum | Klebsiella_pneumoniae;Escherichia_coli;Streptococcus_pneumoniae;Moraxella_catarrhalis;Stenotrophomonas_maltophilia;Acinetobacter_baumannii;Burkholderia_cepacia |
| BE7 | Sputum | Enterobacter_cloacae | Staphylococcus_epidermidis | 8.2% | Stenotrophomonas_maltophilia;Staphylococcus_aureus | Achromobacter_xylosoxidans;Corynebacterium_striatum;Enterococcus_faecium |
| BE7 | Sputum | Enterobacter_cloacae | Stenotrophomonas_maltophilia | 47.8% | Stenotrophomonas_maltophilia;Staphylococcus_aureus | Achromobacter_xylosoxidans;Corynebacterium_striatum;Enterococcus_faecium |
| BF5 | Sputum |  | Corynebacterium_striatum | 48.9% | Haemophilus_influenzae | Achromobacter_xylosoxidans;Corynebacterium_striatum;Enterococcus_faecium;Stenotrophomonas_maltophilia |
| BF5 | Sputum |  | Haemophilus_influenzae | 20.1% | Haemophilus_influenzae | Achromobacter_xylosoxidans;Corynebacterium_striatum;Enterococcus_faecium;Stenotrophomonas_maltophilia |
| BF5 | Sputum |  | Parvimonas_micra | 15.6% | Haemophilus_influenzae | Achromobacter_xylosoxidans;Corynebacterium_striatum;Enterococcus_faecium;Stenotrophomonas_maltophilia |
| BF6 | Sputum | Pseudomonas_aeruginosa | Pseudomonas_aeruginosa | 95.2% |  | Enterococcus_faecium;Stenotrophomonas_maltophilia;Achromobacter_xylosoxidans;Corynebacterium_striatum |
| BF9 | Sputum | Stenotrophomonas_maltophilia | Enterococcus_faecium | 82.2% | Enterococcus_faecium;Pseudomonas_aeruginosa | Corynebacterium_striatum;Enterococcus_faecalis;Achromobacter_xylosoxidans;Staphylococcus_aureus |
| BF9 | Sputum | Stenotrophomonas_maltophilia | Stenotrophomonas_maltophilia | 16.9% | Enterococcus_faecium;Pseudomonas_aeruginosa | Corynebacterium_striatum;Enterococcus_faecalis;Achromobacter_xylosoxidans;Staphylococcus_aureus |
| BG2 | BALF | Pseudomonas_aeruginosa | Pseudomonas_aeruginosa | 75.3% | Pseudomonas_aeruginosa | Stenotrophomonas_maltophilia |
| BG2 | BALF | Pseudomonas_aeruginosa | Veillonella_parvula | 2.7% | Pseudomonas_aeruginosa | Stenotrophomonas_maltophilia |
| BG5 | BALF | Corynebacterium_striatum | Corynebacterium_striatum | 83.7% | Corynebacterium_striatum;Pseudomonas_aeruginosa | Stenotrophomonas_maltophilia |
| BG5 | BALF | Corynebacterium_striatum | Pseudomonas_aeruginosa | 12.2% | Corynebacterium_striatum;Pseudomonas_aeruginosa | Stenotrophomonas_maltophilia |
| BH2 | BALF | Pseudomonas_aeruginosa | Pseudomonas_aeruginosa | 22.9% |  | Streptococcus_pneumoniae;Corynebacterium_striatum |
| BH4 | BALF | Burkholderia_cepacia | Burkholderia_cepacia | 11.4% | Pseudomonas_aeruginosa;Burkholderia_cepacia | Corynebacterium_striatum |
| BH10 | Sputum | Stenotrophomonas_maltophilia;Corynebacterium_striatum | Corynebacterium_striatum | 86.8% | Stenotrophomonas_maltophilia;Corynebacterium_striatum | Streptococcus_pneumoniae;Pseudomonas_aeruginosa;Klebsiella_pneumoniae;Escherichia_coli |
| BH10 | Sputum | Stenotrophomonas_maltophilia;Corynebacterium_striatum | Stenotrophomonas_maltophilia | 2.9% | Stenotrophomonas_maltophilia;Corynebacterium_striatum | Streptococcus_pneumoniae;Pseudomonas_aeruginosa;Klebsiella_pneumoniae;Escherichia_coli |
| BH11 | Sputum |  | Rothia_mucilaginosa | 30.9% | Streptococcus_pneumoniae;Pseudomonas_aeruginosa | Enterococcus_faecalis;Corynebacterium_striatum;Enterococcus_faecium;Staphylococcus_aureus |
| BH11 | Sputum |  | Streptococcus_mitis | 6.9% | Streptococcus_pneumoniae;Pseudomonas_aeruginosa | Enterococcus_faecalis;Corynebacterium_striatum;Enterococcus_faecium;Staphylococcus_aureus |
| BH13 | Sputum | Moraxella_catarrhalis | Moraxella_catarrhalis | 92.4% | Moraxella_catarrhalis;Streptococcus_pneumoniae;Pseudomonas_aeruginosa | Enterococcus_faecium;Achromobacter_xylosoxidans;Haemophilus_influenzae;Stenotrophomonas_maltophilia |
| BH13 | Sputum | Moraxella_catarrhalis | Rothia_mucilaginosa | 1.7% | Moraxella_catarrhalis;Streptococcus_pneumoniae;Pseudomonas_aeruginosa | Enterococcus_faecium;Achromobacter_xylosoxidans;Haemophilus_influenzae;Stenotrophomonas_maltophilia |
| CB4 | Sputum |  | Porphyromonas_gingivalis | 26.5% |  |  |
| CB4 | Sputum |  | Rothia_mucilaginosa | 15.9% |  |  |
| CB4 | Sputum |  | Streptococcus_parasanguinis | 15.4% |  |  |
| CB4 | Sputum |  | Streptococcus_salivarius | 14.5% |  |  |
| CC2 | Sputum |  | Enterococcus_faecium | 65.2% | Enterococcus_faecium | Pseudomonas_aeruginosa |
| CC5 | Sputum |  | Pseudomonas_aeruginosa | 1.3% | Streptococcus_pneumoniae |  |
| CC5 | Sputum |  | Streptococcus_mitis | 55.5% | Streptococcus_pneumoniae |  |
| CC5 | Sputum |  | Streptococcus_pneumoniae | 10.5% | Streptococcus_pneumoniae |  |
| CC6 | Sputum | Streptococcus_pneumoniae | Rothia_mucilaginosa | 15.4% | Streptococcus_pneumoniae |  |
| CC6 | Sputum | Streptococcus_pneumoniae | Streptococcus_parasanguinis | 18.8% | Streptococcus_pneumoniae |  |
| CC6 | Sputum | Streptococcus_pneumoniae | Streptococcus_pneumoniae | 6.7% | Streptococcus_pneumoniae |  |
| CC6 | Sputum | Streptococcus_pneumoniae | Streptococcus_salivarius | 18.3% | Streptococcus_pneumoniae |  |
| CF2 | BALF |  | Acinetobacter_baumannii | 44.7% | Acinetobacter_baumannii;Pseudomonas_aeruginosa |  |
| CF2 | BALF |  | Pseudomonas_aeruginosa | 15.8% | Acinetobacter_baumannii;Pseudomonas_aeruginosa |  |
| CH5 | BALF | Pseudomonas_aeruginosa;Burkholderia_cepacia | Acinetobacter_baumannii | 5.3% | Burkholderia_cepacia;Acinetobacter_baumannii;Pseudomonas_aeruginosa |  |
| CH5 | BALF | Pseudomonas_aeruginosa;Burkholderia_cepacia | Pseudomonas_aeruginosa | 92.5% | Burkholderia_cepacia;Acinetobacter_baumannii;Pseudomonas_aeruginosa |  |
| CI9 | Sputum | Pseudomonas_aeruginosa;Burkholderia_cepacia | Burkholderia_cepacia | 95.5% | Burkholderia_cepacia;Stenotrophomonas_maltophilia |  |
| CI9 | Sputum | Pseudomonas_aeruginosa;Burkholderia_cepacia | Stenotrophomonas_maltophilia | 3.3% | Burkholderia_cepacia;Stenotrophomonas_maltophilia |  |
| CI11 | Sputum | Staphylococcus_aureus | Staphylococcus_aureus | 31.2% | Burkholderia_cepacia;Staphylococcus_aureus | Stenotrophomonas_maltophilia;Streptococcus_pneumoniae |
| CI11 | Sputum | Staphylococcus_aureus | Streptococcus_constellatus | 8.1% | Burkholderia_cepacia;Staphylococcus_aureus | Stenotrophomonas_maltophilia;Streptococcus_pneumoniae |
| CI12 | Sputum | Klebsiella_pneumoniae | Corynebacterium_striatum | 34.2% | Corynebacterium_striatum | Klebsiella_pneumoniae |
| CI12 | Sputum | Klebsiella_pneumoniae | Klebsiella_pneumoniae | 3.1% | Corynebacterium_striatum | Klebsiella_pneumoniae |
| CI12 | Sputum | Klebsiella_pneumoniae | Staphylococcus_epidermidis | 44.2% | Corynebacterium_striatum | Klebsiella_pneumoniae |
| CI12 | Sputum | Klebsiella_pneumoniae | Streptococcus_oralis | 6.9% | Corynebacterium_striatum | Klebsiella_pneumoniae |
| CJ1 | BALF | Acinetobacter_baumannii | Acinetobacter_baumannii | 2.4% | Corynebacterium_striatum;Enterococcus_faecium;Acinetobacter_baumannii | Pseudomonas_aeruginosa |
| CJ1 | BALF | Acinetobacter_baumannii | Corynebacterium_striatum | 89.2% | Corynebacterium_striatum;Enterococcus_faecium;Acinetobacter_baumannii | Pseudomonas_aeruginosa |
| CJ8 | Sputum | Pseudomonas_aeruginosa | Pseudomonas_aeruginosa | 96.5% | Pseudomonas_aeruginosa | Stenotrophomonas_maltophilia |
| CJ8 | Sputum | Pseudomonas_aeruginosa | Stenotrophomonas_maltophilia | 1.8% | Pseudomonas_aeruginosa | Stenotrophomonas_maltophilia |
| CH3 | BALF |  | Tannerella_forsythia | 23.6% | Pseudomonas_aeruginosa |  |
| CH3 | BALF |  | Treponema_denticola | 30.0% | Pseudomonas_aeruginosa |  |
| CH9 | Sputum | Pseudomonas_aeruginosa | Pseudomonas_aeruginosa | 81.2% | Pseudomonas_aeruginosa |  |
| CH9 | Sputum | Pseudomonas_aeruginosa | Streptococcus_oralis | 5.6% | Pseudomonas_aeruginosa |  |
| CI8 | BALF | Corynebacterium_striatum | Corynebacterium_striatum | 6.7% | Corynebacterium_striatum;Streptococcus_pneumoniae;Pseudomonas_aeruginosa |  |
| CI8 | BALF | Corynebacterium_striatum | Streptococcus_pneumoniae | 78.7% | Corynebacterium_striatum;Streptococcus_pneumoniae;Pseudomonas_aeruginosa |  |
| CM2 | BALF | Pseudomonas_aeruginosa;Acinetobacter_baumannii | Acinetobacter_baumannii | 95.6% | Pseudomonas_aeruginosa;Acinetobacter_baumannii |  |
| CM2 | BALF | Pseudomonas_aeruginosa;Acinetobacter_baumannii | Pseudomonas_aeruginosa | 4.4% | Pseudomonas_aeruginosa;Acinetobacter_baumannii |  |
| CM3 | BALF |  | Pseudomonas_aeruginosa | 35.3% | Pseudomonas_aeruginosa | Acinetobacter_baumannii |
| CN8 | BALF |  | Pseudomonas_aeruginosa | 31.3% | Pseudomonas_aeruginosa | Acinetobacter_baumannii |
| CN12_XX | BALF | Pseudomonas_aeruginosa;Acinetobacter_baumannii | Pseudomonas_aeruginosa | 43.9% | Acinetobacter_baumannii;Pseudomonas_aeruginosa | Burkholderia_cepacia |
| CO2 | BALF | Pseudomonas_aeruginosa;Acinetobacter_baumannii | Acinetobacter_baumannii | 94.3% | Staphylococcus_aureus;Acinetobacter_baumannii |  |
| CO2 | BALF | Pseudomonas_aeruginosa;Acinetobacter_baumannii | Parvimonas_micra | 1.6% | Staphylococcus_aureus;Acinetobacter_baumannii |  |
| CR9 | Sputum | Acinetobacter_baumannii | Bifidobacterium_breve | 27.0% |  |  |
| CR9 | Sputum | Acinetobacter_baumannii | Lactobacillus_paracasei | 10.6% |  |  |
| CR9 | Sputum | Acinetobacter_baumannii | Streptococcus_parasanguinis | 11.2% |  |  |
| CR1 | BALF |  | Enterococcus_faecium | 85.0% | Enterococcus_faecium | Pseudomonas_aeruginosa |
| CR1 | BALF |  | Pseudomonas_aeruginosa | 5.6% | Enterococcus_faecium | Pseudomonas_aeruginosa |
| CR1 | BALF |  | Staphylococcus_haemolyticus | 4.7% | Enterococcus_faecium | Pseudomonas_aeruginosa |
| CR4 | BALF | Klebsiella_pneumoniae | Corynebacterium_striatum | 59.8% | Corynebacterium_striatum;Klebsiella_pneumoniae |  |
| CR4 | BALF | Klebsiella_pneumoniae | Klebsiella_pneumoniae | 29.5% | Corynebacterium_striatum;Klebsiella_pneumoniae |  |
| CR4 | BALF | Klebsiella_pneumoniae | Veillonella_parvula | 5.9% | Corynebacterium_striatum;Klebsiella_pneumoniae |  |
| CS2 | BALF | Pseudomonas_aeruginosa | Pseudomonas_aeruginosa | 97.8% | Pseudomonas_aeruginosa |  |
| CS8 | BALF | Klebsiella_pneumoniae | Klebsiella_pneumoniae | 75.2% |  | Pseudomonas_aeruginosa |
| CS13 | Sputum |  | Haemophilus_influenzae | 20.8% | Haemophilus_influenzae | Pseudomonas_aeruginosa |
| CS14 | Sputum |  | Prevotella_denticola | 53.8% |  | Pseudomonas_aeruginosa |
| CT6 | BALF |  | Actinomyces_pacaensis | 12.2% |  |  |
| CT6 | BALF |  | Lactobacillus_reuteri | 14.6% |  |  |
| CT6 | BALF |  | Staphylococcus_epidermidis | 19.5% |  |  |
| CT6 | BALF |  | Staphylococcus_haemolyticus | 14.6% |  |  |
| CU11_lxx | Sputum | Pseudomonas_aeruginosa | Pseudomonas_aeruginosa | 99.3% | Pseudomonas_aeruginosa |  |
| CV12_lxx | Sputum | Acinetobacter_baumannii | Acinetobacter_baumannii | 59.0% | Corynebacterium_striatum;Acinetobacter_baumannii |  |
| CV12_lxx | Sputum | Acinetobacter_baumannii | Corynebacterium_striatum | 31.7% | Corynebacterium_striatum;Acinetobacter_baumannii |  |
| CV12_lxx | Sputum | Acinetobacter_baumannii | Streptococcus_oralis | 4.3% | Corynebacterium_striatum;Acinetobacter_baumannii |  |
| CU5 | BALF |  | Mycoplasma_hominis | 29.4% | Pseudomonas_aeruginosa |  |
| CU5 | BALF |  | Pseudomonas_aeruginosa | 58.8% | Pseudomonas_aeruginosa |  |
| CU6 | BALF |  | Pseudomonas_aeruginosa | 61.9% | Pseudomonas_aeruginosa |  |
| CV3 | BALF | Haemophilus_influenzae | Haemophilus_influenzae | 93.2% | Pseudomonas_aeruginosa;Haemophilus_influenzae |  |
| CV10 | BALF |  | Pseudomonas_aeruginosa | 9.5% | Pseudomonas_aeruginosa | Corynebacterium_striatum;Acinetobacter_baumannii |
| CV14 | Sputum |  | Corynebacterium_striatum | 87.7% | Corynebacterium_striatum | Acinetobacter_baumannii |
| CW2 | BALF | Acinetobacter_baumannii;Escherichia_coli | Acinetobacter_baumannii | 97.2% | Acinetobacter_baumannii | Escherichia_coli |
| CW2 | BALF | Acinetobacter_baumannii;Escherichia_coli | Escherichia_coli | 2.8% | Acinetobacter_baumannii | Escherichia_coli |
| CY4 | BALF | Stenotrophomonas_maltophilia;Acinetobacter_baumannii | Stenotrophomonas_maltophilia | 93.6% | Stenotrophomonas_maltophilia |  |
| CZ4 | BALF |  | Pseudomonas_aeruginosa | 30.0% | Pseudomonas_aeruginosa |  |
| DA3 | BALF | Stenotrophomonas_maltophilia;Pseudomonas_aeruginosa;Achromobacter_xylosoxidans | Achromobacter_xylosoxidans | 29.6% | Pseudomonas_aeruginosa;Stenotrophomonas_maltophilia;Achromobacter_xylosoxidans |  |
| DA3 | BALF | Stenotrophomonas_maltophilia;Pseudomonas_aeruginosa;Achromobacter_xylosoxidans | Pseudomonas_aeruginosa | 61.4% | Pseudomonas_aeruginosa;Stenotrophomonas_maltophilia;Achromobacter_xylosoxidans |  |
| DC11 | Sputum | Pseudomonas_aeruginosa | Pseudomonas_aeruginosa | 95.9% | Pseudomonas_aeruginosa |  |
| DC7 | Sputum |  | Nocardia_brasiliensis | 22.4% |  | Pseudomonas_aeruginosa |
| DC7 | Sputum |  | Nocardia_farcinica | 9.7% |  | Pseudomonas_aeruginosa |
| DC7 | Sputum |  | Veillonella_atypica | 9.7% |  | Pseudomonas_aeruginosa |
| DC7 | Sputum |  | Veillonella_parvula | 17.2% |  | Pseudomonas_aeruginosa |
| DC8 | Sputum | Klebsiella_pneumoniae;Pseudomonas_aeruginosa | Achromobacter_xylosoxidans | 20.8% | Achromobacter_xylosoxidans | Pseudomonas_aeruginosa;Stenotrophomonas_maltophilia |
| DC8 | Sputum | Klebsiella_pneumoniae;Pseudomonas_aeruginosa | Elizabethkingia_anophelis | 31.2% | Achromobacter_xylosoxidans | Pseudomonas_aeruginosa;Stenotrophomonas_maltophilia |
| DA10 | BALF | Pseudomonas_aeruginosa;Acinetobacter_baumannii | Acinetobacter_baumannii | 91.5% | Pseudomonas_aeruginosa;Acinetobacter_baumannii;Burkholderia_cepacia |  |
| DE3 | BALF |  | Prevotella_melaninogenica | 10.3% |  | Pseudomonas_aeruginosa |
| DE3 | BALF |  | Rothia_mucilaginosa | 8.6% |  | Pseudomonas_aeruginosa |
| DE5 | Sputum | Pseudomonas_aeruginosa | Pseudomonas_aeruginosa | 84.6% | Pseudomonas_aeruginosa |  |
| DE5 | Sputum | Pseudomonas_aeruginosa | Streptococcus_anginosus | 5.0% | Pseudomonas_aeruginosa |  |
| DE6 | Sputum | Acinetobacter_baumannii;Pseudomonas_aeruginosa | Pseudomonas_aeruginosa | 96.4% | Pseudomonas_aeruginosa | Acinetobacter_baumannii |
| DD2 | BALF | Pseudomonas_aeruginosa | Pseudomonas_aeruginosa | 68.5% | Pseudomonas_aeruginosa | Klebsiella_pneumoniae |
| DD10 | Sputum | Klebsiella_pneumoniae | Bifidobacterium_longum | 19.6% |  | Klebsiella_pneumoniae;Streptococcus_pneumoniae |
| DD10 | Sputum | Klebsiella_pneumoniae | Streptococcus_parasanguinis | 20.8% |  | Klebsiella_pneumoniae;Streptococcus_pneumoniae |
| DD12 | Sputum |  | Enterococcus_faecium | 6.4% | Enterococcus_faecium |  |
| DD12 | Sputum |  | Rothia_mucilaginosa | 70.8% | Enterococcus_faecium |  |
| DD12 | Sputum |  | Staphylococcus_epidermidis | 21.3% | Enterococcus_faecium |  |
| DF9 | BALF | Klebsiella_pneumoniae | Klebsiella_pneumoniae | 99.2% | Klebsiella_pneumoniae |  |
| DJ14 | Sputum |  | Haemophilus_parainfluenzae | 8.1% |  |  |
| DJ14 | Sputum |  | Neisseria_meningitidis | 29.7% |  |  |
| DJ14 | Sputum |  | Parvimonas_micra | 28.8% |  |  |
| DK4 | BALF | Achromobacter_xylosoxidans | Achromobacter_xylosoxidans | 52.9% | Achromobacter_xylosoxidans | Pseudomonas_aeruginosa |
| DK6 | BALF |  | Streptococcus_pneumoniae | 80.0% | Pseudomonas_aeruginosa;Streptococcus_pneumoniae |  |
| DK15 | BALF |  | Corynebacterium_striatum | 3.8% |  | Corynebacterium_striatum |
| DK15 | BALF |  | Staphylococcus_epidermidis | 58.7% |  | Corynebacterium_striatum |
| DK16 | BALF |  | Pseudomonas_aeruginosa | 71.4% |  | Pseudomonas_aeruginosa |
| DI8 | Sputum |  | Achromobacter_xylosoxidans | 17.1% | Pseudomonas_aeruginosa;Corynebacterium_striatum;Stenotrophomonas_maltophilia;Achromobacter_xylosoxidans |  |
| DI8 | Sputum |  | Corynebacterium_striatum | 49.6% | Pseudomonas_aeruginosa;Corynebacterium_striatum;Stenotrophomonas_maltophilia;Achromobacter_xylosoxidans |  |
| DI8 | Sputum |  | Pseudomonas_aeruginosa | 20.0% | Pseudomonas_aeruginosa;Corynebacterium_striatum;Stenotrophomonas_maltophilia;Achromobacter_xylosoxidans |  |
| DJ8 | Sputum |  | Prevotella_melaninogenica | 19.3% |  |  |
| DJ8 | Sputum |  | Streptococcus_parasanguinis | 38.0% |  |  |
| DJ8 | Sputum |  | Veillonella_parvula | 7.6% |  |  |
| DG6 | Sputum | Pseudomonas_aeruginosa | Pseudomonas_aeruginosa | 99.0% | Pseudomonas_aeruginosa |  |
| DH8 | Sputum | Acinetobacter_baumannii;Enterobacter_cloacae | Acinetobacter_baumannii | 7.1% |  | Acinetobacter_baumannii;Achromobacter_xylosoxidans |
| DH8 | Sputum | Acinetobacter_baumannii;Enterobacter_cloacae | Pseudopropionibacterium_propionicum | 18.6% |  | Acinetobacter_baumannii;Achromobacter_xylosoxidans |
| DI4 | BALF |  | Moraxella_catarrhalis | 24.0% | Pseudomonas_aeruginosa;Moraxella_catarrhalis |  |
| DI4 | BALF |  | Rothia_mucilaginosa | 8.4% | Pseudomonas_aeruginosa;Moraxella_catarrhalis |  |
| DI4 | BALF |  | Veillonella_parvula | 10.2% | Pseudomonas_aeruginosa;Moraxella_catarrhalis |  |
| DK111 | Sputum | Streptococcus_pneumoniae | Corynebacterium_argentoratense | 24.2% | Streptococcus_pneumoniae |  |
| DK111 | Sputum | Streptococcus_pneumoniae | Streptococcus_mitis | 20.0% | Streptococcus_pneumoniae |  |
| DK111 | Sputum | Streptococcus_pneumoniae | Streptococcus_pneumoniae | 12.3% | Streptococcus_pneumoniae |  |
| DL13 | Sputum |  | Corynebacterium_striatum | 12.4% | Corynebacterium_striatum;Enterococcus_faecalis | Acinetobacter_baumannii |
| DL13 | Sputum |  | Enterococcus_faecalis | 10.0% | Corynebacterium_striatum;Enterococcus_faecalis | Acinetobacter_baumannii |
| DL13 | Sputum |  | Rothia_mucilaginosa | 71.9% | Corynebacterium_striatum;Enterococcus_faecalis | Acinetobacter_baumannii |
| DL16 | Sputum |  | Prevotella_intermedia | 19.2% |  | Streptococcus_pneumoniae;Acinetobacter_baumannii;Pseudomonas_aeruginosa |
| DL16 | Sputum |  | Pseudomonas_aeruginosa | 2.3% |  | Streptococcus_pneumoniae;Acinetobacter_baumannii;Pseudomonas_aeruginosa |
| DL16 | Sputum |  | Rothia_mucilaginosa | 13.1% |  | Streptococcus_pneumoniae;Acinetobacter_baumannii;Pseudomonas_aeruginosa |
| DM1 | BALF | Klebsiella_pneumoniae;Pseudomonas_aeruginosa;Acinetobacter_baumannii | Acinetobacter_baumannii | 63.7% | Corynebacterium_striatum;Klebsiella_pneumoniae;Stenotrophomonas_maltophilia;Acinetobacter_baumannii;Pseudomonas_aeruginosa |  |
| DM1 | BALF | Klebsiella_pneumoniae;Pseudomonas_aeruginosa;Acinetobacter_baumannii | Pseudomonas_aeruginosa | 23.0% | Corynebacterium_striatum;Klebsiella_pneumoniae;Stenotrophomonas_maltophilia;Acinetobacter_baumannii;Pseudomonas_aeruginosa |  |
| DM5 | Sputum | Klebsiella_pneumoniae | Haemophilus_parainfluenzae | 4.4% | Klebsiella_pneumoniae;Pseudomonas_aeruginosa | Acinetobacter_baumannii |
| DM5 | Sputum | Klebsiella_pneumoniae | Pseudomonas_aeruginosa | 83.0% | Klebsiella_pneumoniae;Pseudomonas_aeruginosa | Acinetobacter_baumannii |
| DM9 | Sputum |  | Haemophilus_influenzae | 37.5% | Haemophilus_influenzae | Acinetobacter_baumannii |
| DM9 | Sputum |  | Streptococcus_oralis | 12.5% | Haemophilus_influenzae | Acinetobacter_baumannii |
| DN7 | BALF |  | Pseudomonas_aeruginosa | 44.0% |  | Corynebacterium_striatum;Staphylococcus_aureus;Pseudomonas_aeruginosa |
| DN10 | Sputum | Klebsiella_pneumoniae | Moraxella_catarrhalis | 88.8% | Moraxella_catarrhalis |  |
| DN10 | Sputum | Klebsiella_pneumoniae | Rothia_mucilaginosa | 1.2% | Moraxella_catarrhalis |  |
| DN11 | Sputum | Staphylococcus_aureus;Acinetobacter_baumannii | Corynebacterium_striatum | 46.9% | Corynebacterium_striatum;Staphylococcus_aureus |  |
| DN11 | Sputum | Staphylococcus_aureus;Acinetobacter_baumannii | Prevotella_melaninogenica | 2.3% | Corynebacterium_striatum;Staphylococcus_aureus |  |
| DN11 | Sputum | Staphylococcus_aureus;Acinetobacter_baumannii | Staphylococcus_aureus | 43.7% | Corynebacterium_striatum;Staphylococcus_aureus |  |
| DO9 | Sputum | Acinetobacter_baumannii | Acinetobacter_baumannii | 99.0% | Acinetobacter_baumannii |  |
| DO10 | Sputum |  | Bacteroides_zoogleoformans | 8.9% |  |  |
| DO10 | Sputum |  | Parvimonas_micra | 6.2% |  |  |
| DO10 | Sputum |  | Prevotella_intermedia | 69.3% |  |  |
| DT2 | BALF | Corynebacterium_striatum | Corynebacterium_striatum | 79.8% | Corynebacterium_striatum | Acinetobacter_baumannii;Pseudomonas_aeruginosa |
| DU10 | Sputum |  | Prevotella_melaninogenica | 24.0% |  | Corynebacterium_striatum;Haemophilus_influenzae;Streptococcus_pneumoniae |
| DU10 | Sputum |  | Streptococcus_mitis | 10.6% |  | Corynebacterium_striatum;Haemophilus_influenzae;Streptococcus_pneumoniae |
| DU10 | Sputum |  | Veillonella_parvula | 13.8% |  | Corynebacterium_striatum;Haemophilus_influenzae;Streptococcus_pneumoniae |
| DV1 | BALF | Haemophilus_influenzae | Haemophilus_influenzae | 88.4% |  |  |
| DV1 | BALF | Haemophilus_influenzae | Mycobacterium_intracellulare | 8.1% |  |  |
| DV2 | BALF |  | Olsenella_uli | 8.0% |  | Corynebacterium_striatum;Pseudomonas_aeruginosa |
| DV2 | BALF |  | Parvimonas_micra | 62.1% |  | Corynebacterium_striatum;Pseudomonas_aeruginosa |
| DV2 | BALF |  | Prevotella_intermedia | 10.3% |  | Corynebacterium_striatum;Pseudomonas_aeruginosa |
| DW9 | BALF | Escherichia_coli | Escherichia_coli | 100.0% |  |  |
| DX6 | BALF | Acinetobacter_baumannii | Acinetobacter_baumannii | 97.9% |  | Pseudomonas_aeruginosa |
| DX11 | BALF | Pseudomonas_aeruginosa | Pseudomonas_aeruginosa | 96.3% |  |  |
| DX11 | BALF | Pseudomonas_aeruginosa | Streptococcus_oralis | 1.6% |  |  |
| DX12 | BALF | Acinetobacter_baumannii;Burkholderia_cepacia | Acinetobacter_baumannii | 4.8% |  |  |
| DX12 | BALF | Acinetobacter_baumannii;Burkholderia_cepacia | Burkholderia_cepacia | 93.9% |  |  |
| EA4 | BALF |  | Pseudomonas_aeruginosa | 50.0% |  | Corynebacterium_striatum;Klebsiella_pneumoniae;Pseudomonas_aeruginosa |
| EB2 | BALF |  | Moraxella_catarrhalis | 97.0% | Moraxella_catarrhalis | Klebsiella_pneumoniae;Pseudomonas_aeruginosa |
| EB10 | Sputum | Corynebacterium_striatum | Corynebacterium_striatum | 86.8% |  |  |
| EB10 | Sputum | Corynebacterium_striatum | Streptococcus_oralis | 6.3% |  |  |
| EC5 | BALF |  | Haemophilus_influenzae | 80.6% | Haemophilus_influenzae;Pseudomonas_aeruginosa |  |
| EE1 | BALF | Burkholderia_cepacia | Burkholderia_cepacia | 92.5% |  | Pseudomonas_aeruginosa |
| EE3 | BALF |  | Prevotella_melaninogenica | 10.4% |  | Pseudomonas_aeruginosa |
| EE3 | BALF |  | Rothia_mucilaginosa | 26.4% |  | Pseudomonas_aeruginosa |
| EE3 | BALF |  | Streptococcus_parasanguinis | 11.8% |  | Pseudomonas_aeruginosa |
| EE4 | BALF |  | Parvimonas_micra | 20.3% |  | Acinetobacter_baumannii |
| EE4 | BALF |  | Tannerella_forsythia | 50.8% |  | Acinetobacter_baumannii |
| EH6 | BALF | Acinetobacter_baumannii | Acinetobacter_baumannii | 98.6% |  |  |
| EK1 | BALF | Acinetobacter_baumannii;Pseudomonas_aeruginosa | Acinetobacter_baumannii | 99.3% | Acinetobacter_baumannii;Pseudomonas_aeruginosa |  |
| EK8 | Sputum | Pseudomonas_aeruginosa | Prevotella_melaninogenica | 9.7% |  |  |
| EK8 | Sputum | Pseudomonas_aeruginosa | Pseudomonas_aeruginosa | 23.2% |  |  |
| EK2 | BALF | Klebsiella_pneumoniae | Klebsiella_pneumoniae | 37.9% | Acinetobacter_baumannii | Pseudomonas_aeruginosa;Stenotrophomonas_maltophilia |
| EL6 | BALF | Pseudomonas_aeruginosa;Acinetobacter_baumannii | Acinetobacter_baumannii | 98.1% |  |  |
| EL6 | BALF | Pseudomonas_aeruginosa;Acinetobacter_baumannii | Pseudomonas_aeruginosa | 1.9% |  |  |
| EL18 | Sputum |  | Corynebacterium_argentoratense | 15.5% |  | Acinetobacter_baumannii;Streptococcus_pneumoniae |
| EL18 | Sputum |  | Rothia_mucilaginosa | 28.0% |  | Acinetobacter_baumannii;Streptococcus_pneumoniae |
| EL18 | Sputum |  | Streptococcus_parasanguinis | 12.5% |  | Acinetobacter_baumannii;Streptococcus_pneumoniae |
| EM5 | BALF |  | Streptococcus_pneumoniae | 58.3% | Streptococcus_pneumoniae | Pseudomonas_aeruginosa;Acinetobacter_baumannii |
| EO3 | Sputum |  | Corynebacterium_resistens | 18.3% |  | Stenotrophomonas_maltophilia;Enterococcus_faecium |
| EO3 | Sputum |  | Enterococcus_faecium | 21.3% |  | Stenotrophomonas_maltophilia;Enterococcus_faecium |
| EO3 | Sputum |  | Staphylococcus_haemolyticus | 23.0% |  | Stenotrophomonas_maltophilia;Enterococcus_faecium |
| EO3 | Sputum |  | Stenotrophomonas_maltophilia | 25.3% |  | Stenotrophomonas_maltophilia;Enterococcus_faecium |
| ET3 | BALF |  | Fusobacterium_periodonticum | 6.0% |  |  |
| ET3 | BALF |  | Nocardia_brasiliensis | 23.8% |  |  |
| ET4 | BALF | Klebsiella_oxytoca;Leclercia_adecarboxylata | Klebsiella_oxytoca | 24.3% |  | Pseudomonas_aeruginosa |
| ET4 | BALF | Klebsiella_oxytoca;Leclercia_adecarboxylata | Staphylococcus_epidermidis | 13.6% |  | Pseudomonas_aeruginosa |
| ET4 | BALF | Klebsiella_oxytoca;Leclercia_adecarboxylata | Staphylococcus_haemolyticus | 49.6% |  | Pseudomonas_aeruginosa |
| EU7 | Sputum | Acinetobacter_baumannii | Acinetobacter_baumannii | 65.8% |  | Corynebacterium_striatum |
| EU7 | Sputum | Acinetobacter_baumannii | Corynebacterium_striatum | 28.1% |  | Corynebacterium_striatum |
| EU7 | Sputum | Acinetobacter_baumannii | Streptococcus_constellatus | 2.9% |  | Corynebacterium_striatum |
| EV1 | BALF | Pseudomonas_aeruginosa;Enterobacter_aerogenes | Enterobacter_aerogenes | 56.1% | Enterobacter_aerogenes;Enterococcus_faecalis | Pseudomonas_aeruginosa |
| EV1 | BALF | Pseudomonas_aeruginosa;Enterobacter_aerogenes | Enterococcus_faecalis | 37.6% | Enterobacter_aerogenes;Enterococcus_faecalis | Pseudomonas_aeruginosa |
| EV1 | BALF | Pseudomonas_aeruginosa;Enterobacter_aerogenes | Weissella_cibaria | 1.7% | Enterobacter_aerogenes;Enterococcus_faecalis | Pseudomonas_aeruginosa |
| EX1 | BALF | Klebsiella_pneumoniae | Enterococcus_faecium | 63.3% |  | Pseudomonas_aeruginosa;Enterococcus_faecium |
| EX1 | BALF | Klebsiella_pneumoniae | Klebsiella_pneumoniae | 2.0% |  | Pseudomonas_aeruginosa;Enterococcus_faecium |
| EX1 | BALF | Klebsiella_pneumoniae | Pseudomonas_aeruginosa | 2.0% |  | Pseudomonas_aeruginosa;Enterococcus_faecium |
| EX1 | BALF | Klebsiella_pneumoniae | Streptococcus_oralis | 14.0% |  | Pseudomonas_aeruginosa;Enterococcus_faecium |
| EX6 | Sputum | Pseudomonas_aeruginosa | Prevotella_jejuni | 10.1% |  |  |
| EX6 | Sputum | Pseudomonas_aeruginosa | Prevotella_melaninogenica | 13.0% |  |  |
| EX6 | Sputum | Pseudomonas_aeruginosa | Pseudomonas_aeruginosa | 24.6% |  |  |
| EX6 | Sputum | Pseudomonas_aeruginosa | Veillonella_atypica | 17.4% |  |  |
| EY2 | BALF | Burkholderia_cepacia | Burkholderia_cepacia | 93.8% |  |  |
| FB01 | BALF |  | Porphyromonas_gingivalis | 16.5% |  |  |
| FB01 | BALF |  | Tannerella_forsythia | 76.3% |  |  |
| FB01 | BALF |  | Treponema_denticola | 4.0% |  |  |
| FD2 | BALF |  | Prevotella_melaninogenica | 12.8% |  | Pseudomonas_aeruginosa;Stenotrophomonas_maltophilia |
| FD2 | BALF |  | Pseudomonas_aeruginosa | 14.9% |  | Pseudomonas_aeruginosa;Stenotrophomonas_maltophilia |
| FE3 | BALF | Pseudomonas_aeruginosa | Elizabethkingia_anophelis | 96.4% |  | Pseudomonas_aeruginosa |
| FE3 | BALF | Pseudomonas_aeruginosa | Pseudomonas_aeruginosa | 1.2% |  | Pseudomonas_aeruginosa |
| FF2 | BALF |  | Corynebacterium_striatum | 11.1% |  | Pseudomonas_aeruginosa;Stenotrophomonas_maltophilia;Corynebacterium_striatum |
| FF03 | BALF | Pseudomonas_aeruginosa;Klebsiella_pneumoniae;Escherichia_coli | Escherichia_coli | 3.1% |  |  |
| FF03 | BALF | Pseudomonas_aeruginosa;Klebsiella_pneumoniae;Escherichia_coli | Pseudomonas_aeruginosa | 94.4% |  |  |
| FF04 | BALF | Acinetobacter_baumannii;Burkholderia_multivorans | Acinetobacter_baumannii | 9.4% |  |  |
| FF04 | BALF | Acinetobacter_baumannii;Burkholderia_multivorans | Burkholderia_multivorans | 87.7% |  |  |
| FF8 | BALF |  | Moraxella_catarrhalis | 12.5% | Moraxella_catarrhalis | Pseudomonas_aeruginosa;Stenotrophomonas_maltophilia;Burkholderia_multivorans |
| Study ID | Sample type | Culture Results | Meta-IDs | Abundance | qPCR or Sanger Verified Species | qPCR or Sanger Refuted Species |
| L2 | Sputum |  | Veillonella_parvula | 26.2% |  | Streptococcus_pneumoniae;Enterococcus_faecalis;Stenotrophomonas_maltophilia;Pseudomonas_aeruginosa |
| L10 | Sputum |  | Rothia_mucilaginosa | 15.9% | Pseudomonas_aeruginosa | Enterococcus_faecalis;Enterococcus_faecium;Haemophilus_influenzae;Corynebacterium_striatum;Staphylococcus_aureus;Streptococcus_pneumoniae |
| L10 | Sputum |  | Streptococcus_mitis | 8.4% | Pseudomonas_aeruginosa | Enterococcus_faecalis;Enterococcus_faecium;Haemophilus_influenzae;Corynebacterium_striatum;Staphylococcus_aureus;Streptococcus_pneumoniae |
| L22 | BALF |  | Pseudomonas_aeruginosa | 68.8% | Pseudomonas_aeruginosa |  |
| L24 | BALF | Haemophilus_influenzae | Haemophilus_influenzae | 89.1% | Haemophilus_influenzae |  |
| M3 | BALF | Acinetobacter_baumannii;Klebsiella_pneumoniae | Acinetobacter_baumannii | 96.9% | Klebsiella_pneumoniae;Enterococcus_faecium;Corynebacterium_striatum;Acinetobacter_baumannii | Acinetobacter_pittii;Pseudomonas_aeruginosa;Escherichia_coli |
| M3 | BALF | Acinetobacter_baumannii;Klebsiella_pneumoniae | Klebsiella_pneumoniae | 2.6% | Klebsiella_pneumoniae;Enterococcus_faecium;Corynebacterium_striatum;Acinetobacter_baumannii | Acinetobacter_pittii;Pseudomonas_aeruginosa;Escherichia_coli |
| M7 | BALF |  | Pseudomonas_aeruginosa | 50.0% |  | Acinetobacter_baumannii;Pseudomonas_aeruginosa |
| M8 | BALF |  | Pseudomonas_aeruginosa | 63.0% | Pseudomonas_aeruginosa | Acinetobacter_baumannii;Enterococcus_faecium |
| M9 | BALF | Enterobacter_cloacae | Bacteroides_cellulosilyticus | 5.9% | Enterococcus_faecium;Corynebacterium_striatum;Escherichia_coli;Streptococcus_pneumoniae | Klebsiella_pneumoniae;Acinetobacter_baumannii;Staphylococcus_aureus;Pseudomonas_aeruginosa;Enterococcus_faecalis;Enterobacter_aerogenes;Klebsiella_oxytoca |
| M9 | BALF | Enterobacter_cloacae | Corynebacterium_striatum | 4.4% | Enterococcus_faecium;Corynebacterium_striatum;Escherichia_coli;Streptococcus_pneumoniae | Klebsiella_pneumoniae;Acinetobacter_baumannii;Staphylococcus_aureus;Pseudomonas_aeruginosa;Enterococcus_faecalis;Enterobacter_aerogenes;Klebsiella_oxytoca |
| M9 | BALF | Enterobacter_cloacae | Enterococcus_faecium | 70.2% | Enterococcus_faecium;Corynebacterium_striatum;Escherichia_coli;Streptococcus_pneumoniae | Klebsiella_pneumoniae;Acinetobacter_baumannii;Staphylococcus_aureus;Pseudomonas_aeruginosa;Enterococcus_faecalis;Enterobacter_aerogenes;Klebsiella_oxytoca |
| N1 | BALF | Streptococcus_pneumoniae | Rothia_mucilaginosa | 21.5% | Streptococcus_pneumoniae;Pseudomonas_aeruginosa | Enterococcus_faecium;Enterococcus_faecalis;Acinetobacter_baumannii |
| N1 | BALF | Streptococcus_pneumoniae | Streptococcus_pneumoniae | 10.8% | Streptococcus_pneumoniae;Pseudomonas_aeruginosa | Enterococcus_faecium;Enterococcus_faecalis;Acinetobacter_baumannii |
| N8 | BALF | Escherichia_coli | Pseudomonas_aeruginosa | 68.2% |  | Pseudomonas_aeruginosa;Escherichia_coli |
| O3 | BALF | Acinetobacter_baumannii | Acinetobacter_baumannii | 71.2% | Enterococcus_faecium | Acinetobacter_baumannii;Pseudomonas_aeruginosa |
| O3 | BALF | Acinetobacter_baumannii | Enterococcus_faecium | 16.2% | Enterococcus_faecium | Acinetobacter_baumannii;Pseudomonas_aeruginosa |
| O5 | Sputum | Pseudomonas_aeruginosa | Corynebacterium_striatum | 11.9% | Corynebacterium_striatum;Achromobacter_xylosoxidans;Stenotrophomonas_maltophilia |  |
| O5 | Sputum | Pseudomonas_aeruginosa | Pseudomonas_aeruginosa | 39.1% | Corynebacterium_striatum;Achromobacter_xylosoxidans;Stenotrophomonas_maltophilia |  |
| O5 | Sputum | Pseudomonas_aeruginosa | Stenotrophomonas_maltophilia | 13.6% | Corynebacterium_striatum;Achromobacter_xylosoxidans;Stenotrophomonas_maltophilia |  |
| O6 | Sputum | Klebsiella_pneumoniae | Klebsiella_pneumoniae | 11.9% | Klebsiella_pneumoniae;Pseudomonas_aeruginosa |  |
| O6 | Sputum | Klebsiella_pneumoniae | Pseudomonas_aeruginosa | 8.3% | Klebsiella_pneumoniae;Pseudomonas_aeruginosa |  |
| O6 | Sputum | Klebsiella_pneumoniae | Rhodococcus_qingshengii | 10.1% | Klebsiella_pneumoniae;Pseudomonas_aeruginosa |  |
| O7 | Sputum | Pseudomonas_aeruginosa;Stenotrophomonas_maltophilia | Burkholderia_cepacia | 14.9% | Corynebacterium_striatum;Achromobacter_xylosoxidans;Stenotrophomonas_maltophilia;Burkholderia_cepacia |  |
| O7 | Sputum | Pseudomonas_aeruginosa;Stenotrophomonas_maltophilia | Corynebacterium_striatum | 31.8% | Corynebacterium_striatum;Achromobacter_xylosoxidans;Stenotrophomonas_maltophilia;Burkholderia_cepacia |  |
| O7 | Sputum | Pseudomonas_aeruginosa;Stenotrophomonas_maltophilia | Pseudomonas_aeruginosa | 29.4% | Corynebacterium_striatum;Achromobacter_xylosoxidans;Stenotrophomonas_maltophilia;Burkholderia_cepacia |  |
| O7 | Sputum | Pseudomonas_aeruginosa;Stenotrophomonas_maltophilia | Stenotrophomonas_maltophilia | 12.4% | Corynebacterium_striatum;Achromobacter_xylosoxidans;Stenotrophomonas_maltophilia;Burkholderia_cepacia |  |
| O8 | Sputum |  | Rhodococcus_qingshengii | 11.4% | Pseudomonas_aeruginosa | Streptococcus_pneumoniae;Acinetobacter_baumannii |
| O8 | Sputum |  | Streptococcus_mitis | 25.0% | Pseudomonas_aeruginosa | Streptococcus_pneumoniae;Acinetobacter_baumannii |
| P2 | BALF | Pseudomonas_aeruginosa | Parvimonas_micra | 15.9% | Enterococcus_faecium | Acinetobacter_baumannii;Klebsiella_pneumoniae;Streptococcus_pneumoniae |
| P2 | BALF | Pseudomonas_aeruginosa | Pseudomonas_aeruginosa | 24.3% | Enterococcus_faecium | Acinetobacter_baumannii;Klebsiella_pneumoniae;Streptococcus_pneumoniae |
| P2 | BALF | Pseudomonas_aeruginosa | Streptococcus_mitis | 26.2% | Enterococcus_faecium | Acinetobacter_baumannii;Klebsiella_pneumoniae;Streptococcus_pneumoniae |
| P2 | BALF | Pseudomonas_aeruginosa | Streptococcus_pneumoniae | 5.6% | Enterococcus_faecium | Acinetobacter_baumannii;Klebsiella_pneumoniae;Streptococcus_pneumoniae |
| P5 | BALF |  | Corynebacterium_striatum | 23.1% | Enterococcus_faecium | Klebsiella_pneumoniae;Acinetobacter_pittii;Corynebacterium_striatum;Pseudomonas_aeruginosa |
| P5 | BALF |  | Enterococcus_faecium | 12.8% | Enterococcus_faecium | Klebsiella_pneumoniae;Acinetobacter_pittii;Corynebacterium_striatum;Pseudomonas_aeruginosa |
| P7 | BALF | Klebsiella_pneumoniae;Acinetobacter_baumannii | Acinetobacter_baumannii | 85.0% | Klebsiella_pneumoniae;Corynebacterium_striatum | Acinetobacter_pittii;Stenotrophomonas_maltophilia;Pseudomonas_aeruginosa;Achromobacter_xylosoxidans;Escherichia_coli;Streptococcus_pneumoniae |
| P7 | BALF | Klebsiella_pneumoniae;Acinetobacter_baumannii | Klebsiella_pneumoniae | 14.0% | Klebsiella_pneumoniae;Corynebacterium_striatum | Acinetobacter_pittii;Stenotrophomonas_maltophilia;Pseudomonas_aeruginosa;Achromobacter_xylosoxidans;Escherichia_coli;Streptococcus_pneumoniae |
| P8 | Sputum |  | Prevotella_jejuni | 10.9% | Klebsiella_pneumoniae;Pseudomonas_aeruginosa | Streptococcus_pneumoniae;Enterococcus_faecalis;Stenotrophomonas_maltophilia;Corynebacterium_striatum;Enterococcus_faecium;Staphylococcus_aureus;Acinetobacter_baumannii |
| P8 | Sputum |  | Streptococcus_parasanguinis | 31.6% | Klebsiella_pneumoniae;Pseudomonas_aeruginosa | Streptococcus_pneumoniae;Enterococcus_faecalis;Stenotrophomonas_maltophilia;Corynebacterium_striatum;Enterococcus_faecium;Staphylococcus_aureus;Acinetobacter_baumannii |
| P8 | Sputum |  | Veillonella_atypica | 24.6% | Klebsiella_pneumoniae;Pseudomonas_aeruginosa | Streptococcus_pneumoniae;Enterococcus_faecalis;Stenotrophomonas_maltophilia;Corynebacterium_striatum;Enterococcus_faecium;Staphylococcus_aureus;Acinetobacter_baumannii |
| P9 | Sputum |  | Bacteroides_heparinolyticus | 28.6% |  | Klebsiella_pneumoniae;Streptococcus_pneumoniae |
| P9 | Sputum |  | Porphyromonas_gingivalis | 57.6% |  | Klebsiella_pneumoniae;Streptococcus_pneumoniae |
| P9 | Sputum |  | Prevotella_intermedia | 3.5% |  | Klebsiella_pneumoniae;Streptococcus_pneumoniae |
| P10 | Sputum |  | Pseudomonas_aeruginosa | 27.2% |  | Klebsiella_pneumoniae;Staphylococcus_aureus;Enterococcus_faecalis;Enterococcus_faecium;Haemophilus_influenzae;Stenotrophomonas_maltophilia |
| P10 | Sputum |  | Veillonella_parvula | 17.7% |  | Klebsiella_pneumoniae;Staphylococcus_aureus;Enterococcus_faecalis;Enterococcus_faecium;Haemophilus_influenzae;Stenotrophomonas_maltophilia |
| Q5 | Sputum |  | Lactobacillus_paracasei | 20.0% | Pseudomonas_aeruginosa | Moraxella_catarrhalis;Streptococcus_pneumoniae;Corynebacterium_striatum |
| Q5 | Sputum |  | Pseudomonas_aeruginosa | 5.2% | Pseudomonas_aeruginosa | Moraxella_catarrhalis;Streptococcus_pneumoniae;Corynebacterium_striatum |
| Q5 | Sputum |  | Rothia_mucilaginosa | 33.0% | Pseudomonas_aeruginosa | Moraxella_catarrhalis;Streptococcus_pneumoniae;Corynebacterium_striatum |
| Q9 | Sputum |  | Streptococcus_mitis | 56.1% |  | Enterococcus_faecalis;Streptococcus_pneumoniae;Klebsiella_pneumoniae;Staphylococcus_aureus;Stenotrophomonas_maltophilia;Moraxella_catarrhalis |
| Q9 | Sputum |  | Streptococcus_pneumoniae | 11.3% |  | Enterococcus_faecalis;Streptococcus_pneumoniae;Klebsiella_pneumoniae;Staphylococcus_aureus;Stenotrophomonas_maltophilia;Moraxella_catarrhalis |
| R5 | Sputum |  | Corynebacterium_striatum | 17.4% | Staphylococcus_aureus;Enterococcus_faecium;Corynebacterium_striatum | Streptococcus_pneumoniae;Enterococcus_faecalis;Stenotrophomonas_maltophilia;Pseudomonas_aeruginosa; |
| R5 | Sputum |  | Staphylococcus_aureus | 4.5% | Staphylococcus_aureus;Enterococcus_faecium;Corynebacterium_striatum | Streptococcus_pneumoniae;Enterococcus_faecalis;Stenotrophomonas_maltophilia;Pseudomonas_aeruginosa; |
| R5 | Sputum |  | Streptococcus_oralis | 15.2% | Staphylococcus_aureus;Enterococcus_faecium;Corynebacterium_striatum | Streptococcus_pneumoniae;Enterococcus_faecalis;Stenotrophomonas_maltophilia;Pseudomonas_aeruginosa; |
| R6 | Sputum |  | Enterococcus_faecium | 3.1% | Enterococcus_faecium | Streptococcus_pneumoniae;Pseudomonas_aeruginosa |
| R6 | Sputum |  | Lactobacillus_pentosus | 14.2% | Enterococcus_faecium | Streptococcus_pneumoniae;Pseudomonas_aeruginosa |
| R6 | Sputum |  | Rothia_mucilaginosa | 27.2% | Enterococcus_faecium | Streptococcus_pneumoniae;Pseudomonas_aeruginosa |
| R6 | Sputum |  | Streptococcus_pneumoniae | 1.7% | Enterococcus_faecium | Streptococcus_pneumoniae;Pseudomonas_aeruginosa |
| R6 | Sputum |  | Veillonella_parvula | 14.0% | Enterococcus_faecium | Streptococcus_pneumoniae;Pseudomonas_aeruginosa |
| S2 | BALF |  | Veillonella_parvula | 11.6% |  | Stenotrophomonas_maltophilia;Haemophilus_influenzae;Pseudomonas_aeruginosa;Corynebacterium_striatum |
| S3 | Sputum |  | Fusobacterium_necrophorum | 13.2% |  |  |
| S3 | Sputum |  | Fusobacterium_nucleatum | 26.3% |  |  |
| S3 | Sputum |  | Parvimonas_micra | 30.9% |  |  |
| S4 | Sputum |  | Rothia_mucilaginosa | 67.0% |  | Corynebacterium_striatum;Streptococcus_pneumoniae;Pseudomonas_aeruginosa;Enterococcus_faecalis;Achromobacter_xylosoxidans;Stenotrophomonas_maltophilia |
| S4 | Sputum |  | Veillonella_parvula | 4.4% |  | Corynebacterium_striatum;Streptococcus_pneumoniae;Pseudomonas_aeruginosa;Enterococcus_faecalis;Achromobacter_xylosoxidans;Stenotrophomonas_maltophilia |
| S11 | Sputum |  | Haemophilus_influenzae | 29.4% | Haemophilus_influenzae;Pseudomonas_aeruginosa | Corynebacterium_striatum;Acinetobacter_baumannii |
| S11 | Sputum |  | Lactobacillus_rhamnosus | 15.1% | Haemophilus_influenzae;Pseudomonas_aeruginosa | Corynebacterium_striatum;Acinetobacter_baumannii |
| T3 | BALF |  | Chlamydia_psittaci | 94.8% |  | Acinetobacter_pittii;Corynebacterium_striatum;Acinetobacter_baumannii |
| T4 | BALF |  | Pseudomonas_aeruginosa | 64.7% |  | Streptococcus_pneumoniae;Stenotrophomonas_maltophilia;Pseudomonas_aeruginosa |
| T8 | BALF |  | Streptococcus_mitis | 10.1% |  | Enterococcus_faecium;Streptococcus_pneumoniae;Haemophilus_influenzae;Enterococcus_faecalis;Pseudomonas_aeruginosa;Corynebacterium_striatum |
| T8 | BALF |  | Streptococcus_salivarius | 10.5% |  | Enterococcus_faecium;Streptococcus_pneumoniae;Haemophilus_influenzae;Enterococcus_faecalis;Pseudomonas_aeruginosa;Corynebacterium_striatum |
| T8 | BALF |  | Veillonella_atypica | 8.4% |  | Enterococcus_faecium;Streptococcus_pneumoniae;Haemophilus_influenzae;Enterococcus_faecalis;Pseudomonas_aeruginosa;Corynebacterium_striatum |
| V6 | Sputum | Pseudomonas_aeruginosa | Corynebacterium_striatum | 68.1% | Achromobacter_xylosoxidans;Burkholderia_cepacia;Stenotrophomonas_maltophilia;Corynebacterium_striatum | Haemophilus_influenzae;Klebsiella_pneumoniae |
| V6 | Sputum | Pseudomonas_aeruginosa | Pseudomonas_aeruginosa | 7.1% | Achromobacter_xylosoxidans;Burkholderia_cepacia;Stenotrophomonas_maltophilia;Corynebacterium_striatum | Haemophilus_influenzae;Klebsiella_pneumoniae |
| T10 | BALF | Acinetobacter_baumannii | Acinetobacter_baumannii | 99.7% |  |  |
| T16 | Sputum |  | Haemophilus_influenzae | 56.0% | Haemophilus_influenzae |  |
| T16 | Sputum |  | Pseudomonas_aeruginosa | 32.7% | Haemophilus_influenzae |  |
| T16 | Sputum |  | Rothia_mucilaginosa | 5.7% | Haemophilus_influenzae |  |
| W3 | BALF | Staphylococcus_aureus | Pseudomonas_aeruginosa | 27.3% | Pseudomonas_aeruginosa | Corynebacterium_striatum;Stenotrophomonas_maltophilia |
| W3 | BALF | Staphylococcus_aureus | Staphylococcus_aureus | 26.0% | Pseudomonas_aeruginosa | Corynebacterium_striatum;Stenotrophomonas_maltophilia |
| Y2 | BALF | Klebsiella_pneumoniae | Klebsiella_pneumoniae | 75.8% |  | Klebsiella_pneumoniae;Escherichia_coli;Enterobacter_aerogenes;Klebsiella_oxytoca |
| Y13 | Sputum |  | Corynebacterium_striatum | 95.0% | Corynebacterium_striatum | Streptococcus_pneumoniae;Pseudomonas_aeruginosa;Stenotrophomonas_maltophilia |
| Z1 | BALF |  | Pseudomonas_aeruginosa | 66.7% |  | Pseudomonas_aeruginosa |
| Z5 | BALF | Pseudomonas_aeruginosa | Pseudomonas_aeruginosa | 95.6% | Pseudomonas_aeruginosa | Stenotrophomonas_maltophilia |
| Z7 | BALF |  | Prevotella_jejuni | 42.7% | Staphylococcus_aureus;Pseudomonas_aeruginosa | Streptococcus_pneumoniae |
| Z7 | BALF |  | Rothia_mucilaginosa | 19.2% | Staphylococcus_aureus;Pseudomonas_aeruginosa | Streptococcus_pneumoniae |
| Z7 | BALF |  | Staphylococcus_aureus | 6.1% | Staphylococcus_aureus;Pseudomonas_aeruginosa | Streptococcus_pneumoniae |
| AB3 | BALF | Klebsiella_pneumoniae | Bacteroides_fragilis | 48.2% |  | Klebsiella_pneumoniae;Burkholderia_cepacia;Acinetobacter_baumannii |
| AB3 | BALF | Klebsiella_pneumoniae | Parvimonas_micra | 12.6% |  | Klebsiella_pneumoniae;Burkholderia_cepacia;Acinetobacter_baumannii |
| AB8 | Sputum |  | Filifactor_alocis | 4.3% | Streptococcus_pneumoniae;Haemophilus_influenzae;Pseudomonas_aeruginosa | Enterococcus_faecalis |
| AB8 | Sputum |  | Porphyromonas_gingivalis | 51.9% | Streptococcus_pneumoniae;Haemophilus_influenzae;Pseudomonas_aeruginosa | Enterococcus_faecalis |
| AB8 | Sputum |  | Tannerella_forsythia | 32.2% | Streptococcus_pneumoniae;Haemophilus_influenzae;Pseudomonas_aeruginosa | Enterococcus_faecalis |
| AC1 | BALF |  | Pseudomonas_aeruginosa | 84.0% | Pseudomonas_aeruginosa | Stenotrophomonas_maltophilia;Achromobacter_xylosoxidans;Enterococcus_faecium;Corynebacterium_striatum |
| AC11 | Sputum | Pseudomonas_aeruginosa;Stenotrophomonas_maltophilia | Corynebacterium_striatum | 20.8% | Stenotrophomonas_maltophilia;Corynebacterium_striatum;Achromobacter_xylosoxidans;Klebsiella_pneumoniae;Burkholderia_cepacia | Enterococcus_faecium;Escherichia_coli |
| AC11 | Sputum | Pseudomonas_aeruginosa;Stenotrophomonas_maltophilia | Pseudomonas_aeruginosa | 52.3% | Stenotrophomonas_maltophilia;Corynebacterium_striatum;Achromobacter_xylosoxidans;Klebsiella_pneumoniae;Burkholderia_cepacia | Enterococcus_faecium;Escherichia_coli |
| AC11 | Sputum | Pseudomonas_aeruginosa;Stenotrophomonas_maltophilia | Stenotrophomonas_maltophilia | 14.7% | Stenotrophomonas_maltophilia;Corynebacterium_striatum;Achromobacter_xylosoxidans;Klebsiella_pneumoniae;Burkholderia_cepacia | Enterococcus_faecium;Escherichia_coli |
| AD6 | Sputum |  | Haemophilus_influenzae | 64.4% | Haemophilus_influenzae | Stenotrophomonas_maltophilia;Corynebacterium_striatum;Pseudomonas_aeruginosa;Enterococcus_faecium |
| AD6 | Sputum |  | Pseudomonas_aeruginosa | 16.4% | Haemophilus_influenzae | Stenotrophomonas_maltophilia;Corynebacterium_striatum;Pseudomonas_aeruginosa;Enterococcus_faecium |
| AD7 | Sputum | Pseudomonas_aeruginosa | Pseudomonas_aeruginosa | 99.1% |  | Streptococcus_pneumoniae;Corynebacterium_striatum;Klebsiella_pneumoniae;Achromobacter_xylosoxidans;Enterococcus_faecalis;Stenotrophomonas_maltophilia;Burkholderia_cepacia |
| AD8 | Sputum |  | Rothia_mucilaginosa | 16.9% | Streptococcus_pneumoniae | Haemophilus_influenzae;Pseudomonas_aeruginosa;Corynebacterium_striatum;Enterococcus_faecium;Klebsiella_pneumoniae;Escherichia_coli;Stenotrophomonas_maltophilia;Staphylococcus_aureus;Acinetobacter_baumannii;Enterococcus_faecalis |
| AD8 | Sputum |  | Streptococcus_pneumoniae | 6.0% | Streptococcus_pneumoniae | Haemophilus_influenzae;Pseudomonas_aeruginosa;Corynebacterium_striatum;Enterococcus_faecium;Klebsiella_pneumoniae;Escherichia_coli;Stenotrophomonas_maltophilia;Staphylococcus_aureus;Acinetobacter_baumannii;Enterococcus_faecalis |
| AD8 | Sputum |  | Streptococcus_pseudopneumoniae | 12.1% | Streptococcus_pneumoniae | Haemophilus_influenzae;Pseudomonas_aeruginosa;Corynebacterium_striatum;Enterococcus_faecium;Klebsiella_pneumoniae;Escherichia_coli;Stenotrophomonas_maltophilia;Staphylococcus_aureus;Acinetobacter_baumannii;Enterococcus_faecalis |
| AE2 | BALF |  | Streptococcus_oralis | 43.0% | Enterococcus_faecium | Streptococcus_pneumoniae;Enterococcus_faecalis |
| AE6 | BALF |  | Pseudomonas_aeruginosa | 11.1% | Pseudomonas_aeruginosa | Streptococcus_pneumoniae;Haemophilus_influenzae; |
| AF8 | BALF | Pseudomonas_aeruginosa | Pseudomonas_aeruginosa | 88.3% | Pseudomonas_aeruginosa | Stenotrophomonas_maltophilia |
| AF17 | Sputum | Pseudomonas_aeruginosa | Pseudomonas_aeruginosa | 79.6% | Pseudomonas_aeruginosa |  |
| AG2 | BALF | Streptococcus_pneumoniae | Streptococcus_pneumoniae | 98.7% | Streptococcus_pneumoniae;Pseudomonas_aeruginosa | Stenotrophomonas_maltophilia;Enterococcus_faecalis;Enterococcus_faecium |
| AG6 | Sputum | Acinetobacter_baumannii | Prevotella_melaninogenica | 9.1% | Pseudomonas_aeruginosa | Streptococcus_pneumoniae;Acinetobacter_baumannii;Stenotrophomonas_maltophilia |
| AG6 | Sputum | Acinetobacter_baumannii | Rothia_mucilaginosa | 24.7% | Pseudomonas_aeruginosa | Streptococcus_pneumoniae;Acinetobacter_baumannii;Stenotrophomonas_maltophilia |
| AG6 | Sputum | Acinetobacter_baumannii | Veillonella_parvula | 19.6% | Pseudomonas_aeruginosa | Streptococcus_pneumoniae;Acinetobacter_baumannii;Stenotrophomonas_maltophilia |
| AI5 | BALF |  | Corynebacterium_striatum | 92.8% | Corynebacterium_striatum;Pseudomonas_aeruginosa | Enterococcus_faecalis |
| AI11 | Sputum |  | Streptococcus_mitis | 20.7% | Streptococcus_pneumoniae;Pseudomonas_aeruginosa | Enterococcus_faecium;Corynebacterium_striatum |
| AH9 | Sputum | Acinetobacter_pittii | Acinetobacter_pittii | 80.4% | Acinetobacter_pittii;Pseudomonas_aeruginosa | Acinetobacter_baumannii |
| AI14 | Sputum | Acinetobacter_baumannii | Pseudomonas_aeruginosa | 5.8% | Streptococcus_pneumoniae;Pseudomonas_aeruginosa |  |
| AI14 | Sputum | Acinetobacter_baumannii | Rothia_mucilaginosa | 65.6% | Streptococcus_pneumoniae;Pseudomonas_aeruginosa |  |
| AK2 | BALF |  | Pseudomonas_aeruginosa | 34.1% | Pseudomonas_aeruginosa | Acinetobacter_baumannii;Acinetobacter_pittii |
| AJ10 | Sputum | Acinetobacter_baumannii;Streptococcus_pneumoniae | Acinetobacter_baumannii | 100.0% | Enterococcus_faecium;Pseudomonas_aeruginosa;Acinetobacter_baumannii | Klebsiella_pneumoniae;Acinetobacter_pittii;Escherichia_coli;Enterobacter_aerogenes |
| AJ13 | Sputum | Acinetobacter_nosocomialis | Acinetobacter_baumannii | 38.5% | Staphylococcus_aureus;Corynebacterium_striatum;Pseudomonas_aeruginosa | Acinetobacter_pittii;Stenotrophomonas_maltophilia;Acinetobacter_baumannii |
| AJ13 | Sputum | Acinetobacter_nosocomialis | Staphylococcus_aureus | 42.9% | Staphylococcus_aureus;Corynebacterium_striatum;Pseudomonas_aeruginosa | Acinetobacter_pittii;Stenotrophomonas_maltophilia;Acinetobacter_baumannii |
| AK6 | Sputum |  | Pseudomonas_aeruginosa | 100.0% | Pseudomonas_aeruginosa |  |
| AJ12 | Sputum | Klebsiella_pneumoniae | Klebsiella_pneumoniae | 19.1% | Klebsiella_pneumoniae;Pseudomonas_aeruginosa | Streptococcus_pneumoniae |
| AJ12 | Sputum | Klebsiella_pneumoniae | Prevotella_melaninogenica | 16.9% | Klebsiella_pneumoniae;Pseudomonas_aeruginosa | Streptococcus_pneumoniae |
| AJ12 | Sputum | Klebsiella_pneumoniae | Veillonella_atypica | 18.0% | Klebsiella_pneumoniae;Pseudomonas_aeruginosa | Streptococcus_pneumoniae |
| AJ12 | Sputum | Klebsiella_pneumoniae | Veillonella_parvula | 15.7% | Klebsiella_pneumoniae;Pseudomonas_aeruginosa | Streptococcus_pneumoniae |
| AM5 | BALF | Pseudomonas_aeruginosa | Corynebacterium_striatum | 1.7% | Corynebacterium_striatum | Burkholderia_cepacia |
| AM5 | BALF | Pseudomonas_aeruginosa | Pseudomonas_aeruginosa | 95.3% | Corynebacterium_striatum | Burkholderia_cepacia |
| AL12 | Sputum | Burkholderia_cepacia | Burkholderia_cepacia | 99.5% |  |  |
| AN10 | Sputum |  | Filifactor_alocis | 7.9% | Haemophilus_influenzae;Streptococcus_pneumoniae;Pseudomonas_aeruginosa | Acinetobacter_baumannii;Enterococcus_faecalis;Enterococcus_faecium;Staphylococcus_aureus |
| AN10 | Sputum |  | Parvimonas_micra | 11.4% | Haemophilus_influenzae;Streptococcus_pneumoniae;Pseudomonas_aeruginosa | Acinetobacter_baumannii;Enterococcus_faecalis;Enterococcus_faecium;Staphylococcus_aureus |
| AN10 | Sputum |  | Streptococcus_constellatus | 12.8% | Haemophilus_influenzae;Streptococcus_pneumoniae;Pseudomonas_aeruginosa | Acinetobacter_baumannii;Enterococcus_faecalis;Enterococcus_faecium;Staphylococcus_aureus |
| AO3 | BALF | Pseudomonas_aeruginosa;Acinetobacter_baumannii | Acinetobacter_baumannii | 21.3% | Corynebacterium_striatum;Enterococcus_faecium;Burkholderia_cepacia | Acinetobacter_pittii;Enterococcus_faecalis;Escherichia_coli;Stenotrophomonas_maltophilia |
| AO3 | BALF | Pseudomonas_aeruginosa;Acinetobacter_baumannii | Corynebacterium_striatum | 18.8% | Corynebacterium_striatum;Enterococcus_faecium;Burkholderia_cepacia | Acinetobacter_pittii;Enterococcus_faecalis;Escherichia_coli;Stenotrophomonas_maltophilia |
| AO3 | BALF | Pseudomonas_aeruginosa;Acinetobacter_baumannii | Pseudomonas_aeruginosa | 58.9% | Corynebacterium_striatum;Enterococcus_faecium;Burkholderia_cepacia | Acinetobacter_pittii;Enterococcus_faecalis;Escherichia_coli;Stenotrophomonas_maltophilia |
| AO9 | Sputum | Burkholderia_cepacia | Burkholderia_cepacia | 99.2% | Pseudomonas_aeruginosa;Burkholderia_cepacia | Streptococcus_pneumoniae;Stenotrophomonas_maltophilia;Burkholderia_multivorans |
| AN11 | Sputum |  | Streptococcus_gordonii | 14.3% | Streptococcus_pneumoniae | Haemophilus_influenzae;Enterococcus_faecalis;Pseudomonas_aeruginosa;Corynebacterium_striatum;Enterococcus_faecium;Staphylococcus_aureus |
| AN11 | Sputum |  | Streptococcus_parasanguinis | 9.3% | Streptococcus_pneumoniae | Haemophilus_influenzae;Enterococcus_faecalis;Pseudomonas_aeruginosa;Corynebacterium_striatum;Enterococcus_faecium;Staphylococcus_aureus |
| AO2 | BALF |  | Pseudomonas_aeruginosa | 90.0% | Pseudomonas_aeruginosa | Haemophilus_influenzae;Burkholderia_cepacia |
| AN2 | BALF |  | Corynebacterium_striatum | 56.1% | Corynebacterium_striatum;Enterococcus_faecium;Pseudomonas_aeruginosa | Acinetobacter_baumannii;Stenotrophomonas_maltophilia |
| AN2 | BALF |  | Pseudomonas_aeruginosa | 29.3% | Corynebacterium_striatum;Enterococcus_faecium;Pseudomonas_aeruginosa | Acinetobacter_baumannii;Stenotrophomonas_maltophilia |
| AM8 | Sputum |  | Moraxella_catarrhalis | 87.9% | Moraxella_catarrhalis;Pseudomonas_aeruginosa | Acinetobacter_baumannii;Stenotrophomonas_maltophilia |
| AM8 | Sputum |  | Rothia_mucilaginosa | 9.4% | Moraxella_catarrhalis;Pseudomonas_aeruginosa | Acinetobacter_baumannii;Stenotrophomonas_maltophilia |
| AR7 | Sputum | Pseudomonas_aeruginosa | Pseudomonas_aeruginosa | 53.5% | Streptococcus_pneumoniae | Enterococcus_faecalis;Staphylococcus_aureus |
| AR7 | Sputum | Pseudomonas_aeruginosa | Streptococcus_mitis | 19.0% | Streptococcus_pneumoniae | Enterococcus_faecalis;Staphylococcus_aureus |
| AR7 | Sputum | Pseudomonas_aeruginosa | Streptococcus_parasanguinis | 6.4% | Streptococcus_pneumoniae | Enterococcus_faecalis;Staphylococcus_aureus |
| AR7 | Sputum | Pseudomonas_aeruginosa | Streptococcus_pneumoniae | 5.2% | Streptococcus_pneumoniae | Enterococcus_faecalis;Staphylococcus_aureus |
| AR8 | Sputum |  | Rothia_mucilaginosa | 45.6% |  | Streptococcus_pneumoniae;Pseudomonas_aeruginosa |
| AR8 | Sputum |  | Streptococcus_mitis | 8.1% |  | Streptococcus_pneumoniae;Pseudomonas_aeruginosa |
| AR12 | Sputum | Klebsiella_pneumoniae | Acinetobacter_baumannii | 76.8% | Klebsiella_pneumoniae;Enterococcus_faecium;Pseudomonas_aeruginosa;Acinetobacter_baumannii |  |
| AP3 | BALF |  | Acinetobacter_baumannii | 20.9% | Pseudomonas_aeruginosa | Acinetobacter_baumannii;Corynebacterium_striatum;Stenotrophomonas_maltophilia;Acinetobacter_baumannii |
| AP3 | BALF |  | Corynebacterium_striatum | 48.6% | Pseudomonas_aeruginosa | Acinetobacter_baumannii;Corynebacterium_striatum;Stenotrophomonas_maltophilia;Acinetobacter_baumannii |
| AP3 | BALF |  | Pseudomonas_aeruginosa | 11.5% | Pseudomonas_aeruginosa | Acinetobacter_baumannii;Corynebacterium_striatum;Stenotrophomonas_maltophilia;Acinetobacter_baumannii |
| AP3 | BALF |  | Stenotrophomonas_maltophilia | 14.2% | Pseudomonas_aeruginosa | Acinetobacter_baumannii;Corynebacterium_striatum;Stenotrophomonas_maltophilia;Acinetobacter_baumannii |
| AP4 | BALF | Klebsiella_pneumoniae | Klebsiella_pneumoniae | 99.0% | Klebsiella_pneumoniae;Pseudomonas_aeruginosa | Acinetobacter_baumannii;Corynebacterium_striatum |
| AR2 | BALF | Acinetobacter_baumannii;Pseudomonas_aeruginosa | Acinetobacter_baumannii | 18.1% | Corynebacterium_striatum;Stenotrophomonas_maltophilia | Klebsiella_pneumoniae;Achromobacter_xylosoxidans;Escherichia_coli;Burkholderia_cepacia;Enterobacter_aerogenes;Acinetobacter_baumannii;Acinetobacter_pittii |
| AR2 | BALF | Acinetobacter_baumannii;Pseudomonas_aeruginosa | Corynebacterium_striatum | 55.1% | Corynebacterium_striatum;Stenotrophomonas_maltophilia | Klebsiella_pneumoniae;Achromobacter_xylosoxidans;Escherichia_coli;Burkholderia_cepacia;Enterobacter_aerogenes;Acinetobacter_baumannii;Acinetobacter_pittii |
| AR2 | BALF | Acinetobacter_baumannii;Pseudomonas_aeruginosa | Pseudomonas_aeruginosa | 14.3% | Corynebacterium_striatum;Stenotrophomonas_maltophilia | Klebsiella_pneumoniae;Achromobacter_xylosoxidans;Escherichia_coli;Burkholderia_cepacia;Enterobacter_aerogenes;Acinetobacter_baumannii;Acinetobacter_pittii |
| AR2 | BALF | Acinetobacter_baumannii;Pseudomonas_aeruginosa | Stenotrophomonas_maltophilia | 10.6% | Corynebacterium_striatum;Stenotrophomonas_maltophilia | Klebsiella_pneumoniae;Achromobacter_xylosoxidans;Escherichia_coli;Burkholderia_cepacia;Enterobacter_aerogenes;Acinetobacter_baumannii;Acinetobacter_pittii |
| AR15 | Sputum | Staphylococcus_aureus | Staphylococcus_aureus | 99.5% | Staphylococcus_aureus | Stenotrophomonas_maltophilia;Pseudomonas_aeruginosa |
| AS1 | BALF | Pseudomonas_aeruginosa | Pseudomonas_aeruginosa | 99.8% | Pseudomonas_aeruginosa | Staphylococcus_aureus |
| AS3 | BALF | Moraxella_catarrhalis | Moraxella_catarrhalis | 97.4% | Moraxella_catarrhalis;Pseudomonas_aeruginosa | Staphylococcus_aureus |
| AS7 | BALF | Pseudomonas_aeruginosa | Haemophilus_influenzae | 4.5% | Haemophilus_influenzae;Streptococcus_pneumoniae | Corynebacterium_striatum;Enterococcus_faecalis;Klebsiella_pneumoniae;Stenotrophomonas_maltophilia;Staphylococcus_aureus |
| AS7 | BALF | Pseudomonas_aeruginosa | Pseudomonas_aeruginosa | 85.7% | Haemophilus_influenzae;Streptococcus_pneumoniae | Corynebacterium_striatum;Enterococcus_faecalis;Klebsiella_pneumoniae;Stenotrophomonas_maltophilia;Staphylococcus_aureus |
| AS7 | BALF | Pseudomonas_aeruginosa | Streptococcus_anginosus | 2.8% | Haemophilus_influenzae;Streptococcus_pneumoniae | Corynebacterium_striatum;Enterococcus_faecalis;Klebsiella_pneumoniae;Stenotrophomonas_maltophilia;Staphylococcus_aureus |
| AV2 | BALF | Streptococcus_pneumoniae | Streptococcus_pneumoniae | 96.8% | Streptococcus_pneumoniae | Klebsiella_pneumoniae |
| AV6 | BALF | Staphylococcus_aureus | Staphylococcus_aureus | 91.2% | Staphylococcus_aureus;Pseudomonas_aeruginosa | Klebsiella_pneumoniae;Acinetobacter_baumannii |
| AS8 | BALF | Enterobacter_cloacae | Capnocytophaga_leadbetteri | 5.1% | Streptococcus_pneumoniae;Pseudomonas_aeruginosa | Enterococcus_faecalis;Enterococcus_faecium;Klebsiella_pneumoniae;Acinetobacter_baumannii |
| AS8 | BALF | Enterobacter_cloacae | Enterobacter_cloacae | 3.2% | Streptococcus_pneumoniae;Pseudomonas_aeruginosa | Enterococcus_faecalis;Enterococcus_faecium;Klebsiella_pneumoniae;Acinetobacter_baumannii |
| AS8 | BALF | Enterobacter_cloacae | Enterococcus_faecalis | 25.0% | Streptococcus_pneumoniae;Pseudomonas_aeruginosa | Enterococcus_faecalis;Enterococcus_faecium;Klebsiella_pneumoniae;Acinetobacter_baumannii |
| AU2 | BALF | Acinetobacter_baumannii;Pseudomonas_aeruginosa | Acinetobacter_baumannii | 98.3% |  | Acinetobacter_pittii;Klebsiella_pneumoniae;Escherichia_coli;Stenotrophomonas_maltophilia;Acinetobacter_baumannii |
| AV8 | Sputum | Pseudomonas_aeruginosa | Pseudomonas_aeruginosa | 28.6% | Achromobacter_xylosoxidans;Corynebacterium_striatum | Enterococcus_faecalis;Klebsiella_pneumoniae;Escherichia_coli;Streptococcus_pneumoniae |
| AV8 | Sputum | Pseudomonas_aeruginosa | Serratia_marcescens | 32.1% | Achromobacter_xylosoxidans;Corynebacterium_striatum | Enterococcus_faecalis;Klebsiella_pneumoniae;Escherichia_coli;Streptococcus_pneumoniae |
| AV8 | Sputum | Pseudomonas_aeruginosa | Stenotrophomonas_maltophilia | 4.6% | Achromobacter_xylosoxidans;Corynebacterium_striatum | Enterococcus_faecalis;Klebsiella_pneumoniae;Escherichia_coli;Streptococcus_pneumoniae |
| AV8 | Sputum | Pseudomonas_aeruginosa | Streptococcus_oralis | 11.8% | Achromobacter_xylosoxidans;Corynebacterium_striatum | Enterococcus_faecalis;Klebsiella_pneumoniae;Escherichia_coli;Streptococcus_pneumoniae |
| AU5 | Sputum |  | Corynebacterium_striatum | 93.6% | Corynebacterium_striatum | Pseudomonas_aeruginosa;Stenotrophomonas_maltophilia |
| AU6 | Sputum |  | Prevotella_melaninogenica | 33.3% | Pseudomonas_aeruginosa | Corynebacterium_striatum |
| AV5 | BALF |  | Parvimonas_micra | 84.6% |  | Pseudomonas_aeruginosa |
| AW9 | Sputum |  | Actinomyces_pacaensis | 5.2% |  |  |
| AW9 | Sputum |  | Rothia_mucilaginosa | 64.6% |  |  |
| AX8 | BALF | Pseudomonas_aeruginosa | Pseudomonas_mendocina | 97.3% |  |  |
| AX12 | Sputum |  | Lactobacillus_crispatus | 11.0% |  |  |
| AX12 | Sputum |  | Parascardovia_denticolens | 20.7% |  |  |
| AX12 | Sputum |  | Veillonella_parvula | 8.7% |  |  |
| AY4 | BALF |  | Enterococcus_faecium | 29.0% | Enterococcus_faecium;Pseudomonas_aeruginosa |  |
| AY4 | BALF |  | Mycoplasma_pneumoniae | 65.2% | Enterococcus_faecium;Pseudomonas_aeruginosa |  |
| AY9 | Sputum |  | Bacteroides_heparinolyticus | 31.0% |  | Pseudomonas_aeruginosa |
| AY9 | Sputum |  | Parvimonas_micra | 48.3% |  | Pseudomonas_aeruginosa |
| AZ4 | BALF | Enterobacter_cloacae | Pseudomonas_aeruginosa | 15.2% |  | Streptococcus_pneumoniae;Pseudomonas_aeruginosa;Acinetobacter_baumannii |
| AZ4 | BALF | Enterobacter_cloacae | Rothia_mucilaginosa | 47.8% |  | Streptococcus_pneumoniae;Pseudomonas_aeruginosa;Acinetobacter_baumannii |
| AY7 | Sputum | Corynebacterium_striatum | Corynebacterium_striatum | 21.5% | Enterococcus_faecium;Corynebacterium_striatum | Pseudomonas_aeruginosa;Streptococcus_pneumoniae;Enterococcus_faecalis;Acinetobacter_baumannii |
| AY7 | Sputum | Corynebacterium_striatum | Rothia_mucilaginosa | 61.1% | Enterococcus_faecium;Corynebacterium_striatum | Pseudomonas_aeruginosa;Streptococcus_pneumoniae;Enterococcus_faecalis;Acinetobacter_baumannii |
| AX105 | Sputum |  | Streptococcus_pneumoniae | 64.3% | Streptococcus_pneumoniae;Haemophilus_influenzae;Pseudomonas_aeruginosa | Acinetobacter_baumannii |
| BA1 | BALF |  | Pseudomonas_aeruginosa | 88.9% | Pseudomonas_aeruginosa |  |
| BA6 | BALF |  | Haemophilus_influenzae | 98.1% | Haemophilus_influenzae | Acinetobacter_baumannii |
| BA10 | BALF | Acinetobacter_baumannii | Acinetobacter_baumannii | 96.8% | Enterococcus_faecium;Acinetobacter_baumannii | Klebsiella_pneumoniae;Acinetobacter_pittii;Pseudomonas_aeruginosa;Corynebacterium_striatum;Escherichia_coli;Streptococcus_pneumoniae;Staphylococcus_aureus |
| BA10 | BALF | Acinetobacter_baumannii | Lactobacillus_mucosae | 1.3% | Enterococcus_faecium;Acinetobacter_baumannii | Klebsiella_pneumoniae;Acinetobacter_pittii;Pseudomonas_aeruginosa;Corynebacterium_striatum;Escherichia_coli;Streptococcus_pneumoniae;Staphylococcus_aureus |
| BA11 | Sputum |  | Mycobacterium_tuberculosis | 91.4% |  | Streptococcus_pneumoniae;Pseudomonas_aeruginosa;Acinetobacter_baumannii |
| BA15 | Sputum |  | Corynebacterium_simulans | 84.7% |  | Corynebacterium_striatum;Acinetobacter_baumannii;Pseudomonas_aeruginosa;Staphylococcus_aureus;Streptococcus_pneumoniae |
| BA15 | Sputum |  | Rothia_mucilaginosa | 7.8% |  | Corynebacterium_striatum;Acinetobacter_baumannii;Pseudomonas_aeruginosa;Staphylococcus_aureus;Streptococcus_pneumoniae |
| BB11 | Sputum | Klebsiella_pneumoniae;Corynebacterium_striatum | Acinetobacter_baumannii | 30.3% | Staphylococcus_aureus;Corynebacterium_striatum;Streptococcus_pneumoniae;Pseudomonas_aeruginosa;Acinetobacter_baumannii |  |
| BB11 | Sputum | Klebsiella_pneumoniae;Corynebacterium_striatum | Corynebacterium_striatum | 54.8% | Staphylococcus_aureus;Corynebacterium_striatum;Streptococcus_pneumoniae;Pseudomonas_aeruginosa;Acinetobacter_baumannii |  |
| BB11 | Sputum | Klebsiella_pneumoniae;Corynebacterium_striatum | Lactobacillus_gasseri | 7.2% | Staphylococcus_aureus;Corynebacterium_striatum;Streptococcus_pneumoniae;Pseudomonas_aeruginosa;Acinetobacter_baumannii |  |
| BB12 | Sputum | Streptococcus_pneumoniae | Lactobacillus_rhamnosus | 24.5% | Streptococcus_pneumoniae |  |
| BB12 | Sputum | Streptococcus_pneumoniae | Rothia_dentocariosa | 10.6% | Streptococcus_pneumoniae |  |
| BB12 | Sputum | Streptococcus_pneumoniae | Streptococcus_parasanguinis | 11.0% | Streptococcus_pneumoniae |  |
| BB12 | Sputum | Streptococcus_pneumoniae | Streptococcus_pneumoniae | 12.3% | Streptococcus_pneumoniae |  |
| BB15 | Sputum |  | Rothia_mucilaginosa | 19.2% |  | Streptococcus_pneumoniae;Stenotrophomonas_maltophilia;Enterococcus_faecalis;Pseudomonas_aeruginosa;Corynebacterium_striatum |
| BB15 | Sputum |  | Streptococcus_parasanguinis | 16.8% |  | Streptococcus_pneumoniae;Stenotrophomonas_maltophilia;Enterococcus_faecalis;Pseudomonas_aeruginosa;Corynebacterium_striatum |
| BB15 | Sputum |  | Veillonella_parvula | 20.9% |  | Streptococcus_pneumoniae;Stenotrophomonas_maltophilia;Enterococcus_faecalis;Pseudomonas_aeruginosa;Corynebacterium_striatum |
| BB16 | Sputum | Moraxella_catarrhalis | Moraxella_catarrhalis | 18.4% | Streptococcus_pneumoniae;Moraxella_catarrhalis | Enterococcus_faecalis;Corynebacterium_striatum;Haemophilus_influenzae;Staphylococcus_aureus |
| BB16 | Sputum | Moraxella_catarrhalis | Streptococcus_parasanguinis | 20.3% | Streptococcus_pneumoniae;Moraxella_catarrhalis | Enterococcus_faecalis;Corynebacterium_striatum;Haemophilus_influenzae;Staphylococcus_aureus |
| BB16 | Sputum | Moraxella_catarrhalis | Veillonella_atypica | 13.0% | Streptococcus_pneumoniae;Moraxella_catarrhalis | Enterococcus_faecalis;Corynebacterium_striatum;Haemophilus_influenzae;Staphylococcus_aureus |
| BC8 | Sputum |  | Enterococcus_faecalis | 4.7% | Enterococcus_faecium;Enterococcus_faecalis;Pseudomonas_aeruginosa | Streptococcus_pneumoniae;Staphylococcus_aureus |
| BC8 | Sputum |  | Streptococcus_oralis | 45.0% | Enterococcus_faecium;Enterococcus_faecalis;Pseudomonas_aeruginosa | Streptococcus_pneumoniae;Staphylococcus_aureus |
| BD5 | BALF | Corynebacterium_striatum | Corynebacterium_striatum | 60.0% |  | Corynebacterium_striatum;Pseudomonas_aeruginosa |
| BE4 | BALF | Acinetobacter_baumannii | Acinetobacter_baumannii | 91.0% | Pseudomonas_aeruginosa | Achromobacter_xylosoxidans;Stenotrophomonas_maltophilia;Acinetobacter_baumannii |
| BE5 | Sputum | Pseudomonas_aeruginosa;Moraxella_catarrhalis | Moraxella_catarrhalis | 27.3% | Moraxella_catarrhalis | Achromobacter_xylosoxidans;Streptococcus_pneumoniae |
| BE5 | Sputum | Pseudomonas_aeruginosa;Moraxella_catarrhalis | Pseudomonas_aeruginosa | 69.1% | Moraxella_catarrhalis | Achromobacter_xylosoxidans;Streptococcus_pneumoniae |
| BE6 | Sputum | Pseudomonas_aeruginosa | Achromobacter_xylosoxidans | 85.3% | Achromobacter_xylosoxidans;Corynebacterium_striatum | Klebsiella_pneumoniae;Escherichia_coli;Streptococcus_pneumoniae;Moraxella_catarrhalis;Stenotrophomonas_maltophilia;Acinetobacter_baumannii;Burkholderia_cepacia |
| BE6 | Sputum | Pseudomonas_aeruginosa | Pseudomonas_aeruginosa | 12.3% | Achromobacter_xylosoxidans;Corynebacterium_striatum | Klebsiella_pneumoniae;Escherichia_coli;Streptococcus_pneumoniae;Moraxella_catarrhalis;Stenotrophomonas_maltophilia;Acinetobacter_baumannii;Burkholderia_cepacia |
| BE7 | Sputum | Enterobacter_cloacae | Staphylococcus_epidermidis | 8.2% | Stenotrophomonas_maltophilia;Staphylococcus_aureus | Achromobacter_xylosoxidans;Corynebacterium_striatum;Enterococcus_faecium |
| BE7 | Sputum | Enterobacter_cloacae | Stenotrophomonas_maltophilia | 47.8% | Stenotrophomonas_maltophilia;Staphylococcus_aureus | Achromobacter_xylosoxidans;Corynebacterium_striatum;Enterococcus_faecium |
| BF5 | Sputum |  | Corynebacterium_striatum | 48.9% | Haemophilus_influenzae | Achromobacter_xylosoxidans;Corynebacterium_striatum;Enterococcus_faecium;Stenotrophomonas_maltophilia |
| BF5 | Sputum |  | Haemophilus_influenzae | 20.1% | Haemophilus_influenzae | Achromobacter_xylosoxidans;Corynebacterium_striatum;Enterococcus_faecium;Stenotrophomonas_maltophilia |
| BF5 | Sputum |  | Parvimonas_micra | 15.6% | Haemophilus_influenzae | Achromobacter_xylosoxidans;Corynebacterium_striatum;Enterococcus_faecium;Stenotrophomonas_maltophilia |
| BF6 | Sputum | Pseudomonas_aeruginosa | Pseudomonas_aeruginosa | 95.2% |  | Enterococcus_faecium;Stenotrophomonas_maltophilia;Achromobacter_xylosoxidans;Corynebacterium_striatum |
| BF9 | Sputum | Stenotrophomonas_maltophilia | Enterococcus_faecium | 82.2% | Enterococcus_faecium;Pseudomonas_aeruginosa | Corynebacterium_striatum;Enterococcus_faecalis;Achromobacter_xylosoxidans;Staphylococcus_aureus |
| BF9 | Sputum | Stenotrophomonas_maltophilia | Stenotrophomonas_maltophilia | 16.9% | Enterococcus_faecium;Pseudomonas_aeruginosa | Corynebacterium_striatum;Enterococcus_faecalis;Achromobacter_xylosoxidans;Staphylococcus_aureus |
| BG2 | BALF | Pseudomonas_aeruginosa | Pseudomonas_aeruginosa | 75.3% | Pseudomonas_aeruginosa | Stenotrophomonas_maltophilia |
| BG2 | BALF | Pseudomonas_aeruginosa | Veillonella_parvula | 2.7% | Pseudomonas_aeruginosa | Stenotrophomonas_maltophilia |
| BG5 | BALF | Corynebacterium_striatum | Corynebacterium_striatum | 83.7% | Corynebacterium_striatum;Pseudomonas_aeruginosa | Stenotrophomonas_maltophilia |
| BG5 | BALF | Corynebacterium_striatum | Pseudomonas_aeruginosa | 12.2% | Corynebacterium_striatum;Pseudomonas_aeruginosa | Stenotrophomonas_maltophilia |
| BH2 | BALF | Pseudomonas_aeruginosa | Pseudomonas_aeruginosa | 22.9% |  | Streptococcus_pneumoniae;Corynebacterium_striatum |
| BH4 | BALF | Burkholderia_cepacia | Burkholderia_cepacia | 11.4% | Pseudomonas_aeruginosa;Burkholderia_cepacia | Corynebacterium_striatum |
| BH10 | Sputum | Stenotrophomonas_maltophilia;Corynebacterium_striatum | Corynebacterium_striatum | 86.8% | Stenotrophomonas_maltophilia;Corynebacterium_striatum | Streptococcus_pneumoniae;Pseudomonas_aeruginosa;Klebsiella_pneumoniae;Escherichia_coli |
| BH10 | Sputum | Stenotrophomonas_maltophilia;Corynebacterium_striatum | Stenotrophomonas_maltophilia | 2.9% | Stenotrophomonas_maltophilia;Corynebacterium_striatum | Streptococcus_pneumoniae;Pseudomonas_aeruginosa;Klebsiella_pneumoniae;Escherichia_coli |
| BH11 | Sputum |  | Rothia_mucilaginosa | 30.9% | Streptococcus_pneumoniae;Pseudomonas_aeruginosa | Enterococcus_faecalis;Corynebacterium_striatum;Enterococcus_faecium;Staphylococcus_aureus |
| BH11 | Sputum |  | Streptococcus_mitis | 6.9% | Streptococcus_pneumoniae;Pseudomonas_aeruginosa | Enterococcus_faecalis;Corynebacterium_striatum;Enterococcus_faecium;Staphylococcus_aureus |
| BH13 | Sputum | Moraxella_catarrhalis | Moraxella_catarrhalis | 92.4% | Moraxella_catarrhalis;Streptococcus_pneumoniae;Pseudomonas_aeruginosa | Enterococcus_faecium;Achromobacter_xylosoxidans;Haemophilus_influenzae;Stenotrophomonas_maltophilia |
| BH13 | Sputum | Moraxella_catarrhalis | Rothia_mucilaginosa | 1.7% | Moraxella_catarrhalis;Streptococcus_pneumoniae;Pseudomonas_aeruginosa | Enterococcus_faecium;Achromobacter_xylosoxidans;Haemophilus_influenzae;Stenotrophomonas_maltophilia |
| CB4 | Sputum |  | Porphyromonas_gingivalis | 26.5% |  |  |
| CB4 | Sputum |  | Rothia_mucilaginosa | 15.9% |  |  |
| CB4 | Sputum |  | Streptococcus_parasanguinis | 15.4% |  |  |
| CB4 | Sputum |  | Streptococcus_salivarius | 14.5% |  |  |
| CC2 | Sputum |  | Enterococcus_faecium | 65.2% | Enterococcus_faecium | Pseudomonas_aeruginosa |
| CC5 | Sputum |  | Pseudomonas_aeruginosa | 1.3% | Streptococcus_pneumoniae |  |
| CC5 | Sputum |  | Streptococcus_mitis | 55.5% | Streptococcus_pneumoniae |  |
| CC5 | Sputum |  | Streptococcus_pneumoniae | 10.5% | Streptococcus_pneumoniae |  |
| CC6 | Sputum | Streptococcus_pneumoniae | Rothia_mucilaginosa | 15.4% | Streptococcus_pneumoniae |  |
| CC6 | Sputum | Streptococcus_pneumoniae | Streptococcus_parasanguinis | 18.8% | Streptococcus_pneumoniae |  |
| CC6 | Sputum | Streptococcus_pneumoniae | Streptococcus_pneumoniae | 6.7% | Streptococcus_pneumoniae |  |
| CC6 | Sputum | Streptococcus_pneumoniae | Streptococcus_salivarius | 18.3% | Streptococcus_pneumoniae |  |
| CF2 | BALF |  | Acinetobacter_baumannii | 44.7% | Acinetobacter_baumannii;Pseudomonas_aeruginosa |  |
| CF2 | BALF |  | Pseudomonas_aeruginosa | 15.8% | Acinetobacter_baumannii;Pseudomonas_aeruginosa |  |
| CH5 | BALF | Pseudomonas_aeruginosa;Burkholderia_cepacia | Acinetobacter_baumannii | 5.3% | Burkholderia_cepacia;Acinetobacter_baumannii;Pseudomonas_aeruginosa |  |
| CH5 | BALF | Pseudomonas_aeruginosa;Burkholderia_cepacia | Pseudomonas_aeruginosa | 92.5% | Burkholderia_cepacia;Acinetobacter_baumannii;Pseudomonas_aeruginosa |  |
| CI9 | Sputum | Pseudomonas_aeruginosa;Burkholderia_cepacia | Burkholderia_cepacia | 95.5% | Burkholderia_cepacia;Stenotrophomonas_maltophilia |  |
| CI9 | Sputum | Pseudomonas_aeruginosa;Burkholderia_cepacia | Stenotrophomonas_maltophilia | 3.3% | Burkholderia_cepacia;Stenotrophomonas_maltophilia |  |
| CI11 | Sputum | Staphylococcus_aureus | Staphylococcus_aureus | 31.2% | Burkholderia_cepacia;Staphylococcus_aureus | Stenotrophomonas_maltophilia;Streptococcus_pneumoniae |
| CI11 | Sputum | Staphylococcus_aureus | Streptococcus_constellatus | 8.1% | Burkholderia_cepacia;Staphylococcus_aureus | Stenotrophomonas_maltophilia;Streptococcus_pneumoniae |
| CI12 | Sputum | Klebsiella_pneumoniae | Corynebacterium_striatum | 34.2% | Corynebacterium_striatum | Klebsiella_pneumoniae |
| CI12 | Sputum | Klebsiella_pneumoniae | Klebsiella_pneumoniae | 3.1% | Corynebacterium_striatum | Klebsiella_pneumoniae |
| CI12 | Sputum | Klebsiella_pneumoniae | Staphylococcus_epidermidis | 44.2% | Corynebacterium_striatum | Klebsiella_pneumoniae |
| CI12 | Sputum | Klebsiella_pneumoniae | Streptococcus_oralis | 6.9% | Corynebacterium_striatum | Klebsiella_pneumoniae |
| CJ1 | BALF | Acinetobacter_baumannii | Acinetobacter_baumannii | 2.4% | Corynebacterium_striatum;Enterococcus_faecium;Acinetobacter_baumannii | Pseudomonas_aeruginosa |
| CJ1 | BALF | Acinetobacter_baumannii | Corynebacterium_striatum | 89.2% | Corynebacterium_striatum;Enterococcus_faecium;Acinetobacter_baumannii | Pseudomonas_aeruginosa |
| CJ8 | Sputum | Pseudomonas_aeruginosa | Pseudomonas_aeruginosa | 96.5% | Pseudomonas_aeruginosa | Stenotrophomonas_maltophilia |
| CJ8 | Sputum | Pseudomonas_aeruginosa | Stenotrophomonas_maltophilia | 1.8% | Pseudomonas_aeruginosa | Stenotrophomonas_maltophilia |
| CH3 | BALF |  | Tannerella_forsythia | 23.6% | Pseudomonas_aeruginosa |  |
| CH3 | BALF |  | Treponema_denticola | 30.0% | Pseudomonas_aeruginosa |  |
| CH9 | Sputum | Pseudomonas_aeruginosa | Pseudomonas_aeruginosa | 81.2% | Pseudomonas_aeruginosa |  |
| CH9 | Sputum | Pseudomonas_aeruginosa | Streptococcus_oralis | 5.6% | Pseudomonas_aeruginosa |  |
| CI8 | BALF | Corynebacterium_striatum | Corynebacterium_striatum | 6.7% | Corynebacterium_striatum;Streptococcus_pneumoniae;Pseudomonas_aeruginosa |  |
| CI8 | BALF | Corynebacterium_striatum | Streptococcus_pneumoniae | 78.7% | Corynebacterium_striatum;Streptococcus_pneumoniae;Pseudomonas_aeruginosa |  |
| CM2 | BALF | Pseudomonas_aeruginosa;Acinetobacter_baumannii | Acinetobacter_baumannii | 95.6% | Pseudomonas_aeruginosa;Acinetobacter_baumannii |  |
| CM2 | BALF | Pseudomonas_aeruginosa;Acinetobacter_baumannii | Pseudomonas_aeruginosa | 4.4% | Pseudomonas_aeruginosa;Acinetobacter_baumannii |  |
| CM3 | BALF |  | Pseudomonas_aeruginosa | 35.3% | Pseudomonas_aeruginosa | Acinetobacter_baumannii |
| CN8 | BALF |  | Pseudomonas_aeruginosa | 31.3% | Pseudomonas_aeruginosa | Acinetobacter_baumannii |
| CN12_XX | BALF | Pseudomonas_aeruginosa;Acinetobacter_baumannii | Pseudomonas_aeruginosa | 43.9% | Acinetobacter_baumannii;Pseudomonas_aeruginosa | Burkholderia_cepacia |
| CO2 | BALF | Pseudomonas_aeruginosa;Acinetobacter_baumannii | Acinetobacter_baumannii | 94.3% | Staphylococcus_aureus;Acinetobacter_baumannii |  |
| CO2 | BALF | Pseudomonas_aeruginosa;Acinetobacter_baumannii | Parvimonas_micra | 1.6% | Staphylococcus_aureus;Acinetobacter_baumannii |  |
| CR9 | Sputum | Acinetobacter_baumannii | Bifidobacterium_breve | 27.0% |  |  |
| CR9 | Sputum | Acinetobacter_baumannii | Lactobacillus_paracasei | 10.6% |  |  |
| CR9 | Sputum | Acinetobacter_baumannii | Streptococcus_parasanguinis | 11.2% |  |  |
| CR1 | BALF |  | Enterococcus_faecium | 85.0% | Enterococcus_faecium | Pseudomonas_aeruginosa |
| CR1 | BALF |  | Pseudomonas_aeruginosa | 5.6% | Enterococcus_faecium | Pseudomonas_aeruginosa |
| CR1 | BALF |  | Staphylococcus_haemolyticus | 4.7% | Enterococcus_faecium | Pseudomonas_aeruginosa |
| CR4 | BALF | Klebsiella_pneumoniae | Corynebacterium_striatum | 59.8% | Corynebacterium_striatum;Klebsiella_pneumoniae |  |
| CR4 | BALF | Klebsiella_pneumoniae | Klebsiella_pneumoniae | 29.5% | Corynebacterium_striatum;Klebsiella_pneumoniae |  |
| CR4 | BALF | Klebsiella_pneumoniae | Veillonella_parvula | 5.9% | Corynebacterium_striatum;Klebsiella_pneumoniae |  |
| CS2 | BALF | Pseudomonas_aeruginosa | Pseudomonas_aeruginosa | 97.8% | Pseudomonas_aeruginosa |  |
| CS8 | BALF | Klebsiella_pneumoniae | Klebsiella_pneumoniae | 75.2% |  | Pseudomonas_aeruginosa |
| CS13 | Sputum |  | Haemophilus_influenzae | 20.8% | Haemophilus_influenzae | Pseudomonas_aeruginosa |
| CS14 | Sputum |  | Prevotella_denticola | 53.8% |  | Pseudomonas_aeruginosa |
| CT6 | BALF |  | Actinomyces_pacaensis | 12.2% |  |  |
| CT6 | BALF |  | Lactobacillus_reuteri | 14.6% |  |  |
| CT6 | BALF |  | Staphylococcus_epidermidis | 19.5% |  |  |
| CT6 | BALF |  | Staphylococcus_haemolyticus | 14.6% |  |  |
| CU11_lxx | Sputum | Pseudomonas_aeruginosa | Pseudomonas_aeruginosa | 99.3% | Pseudomonas_aeruginosa |  |
| CV12_lxx | Sputum | Acinetobacter_baumannii | Acinetobacter_baumannii | 59.0% | Corynebacterium_striatum;Acinetobacter_baumannii |  |
| CV12_lxx | Sputum | Acinetobacter_baumannii | Corynebacterium_striatum | 31.7% | Corynebacterium_striatum;Acinetobacter_baumannii |  |
| CV12_lxx | Sputum | Acinetobacter_baumannii | Streptococcus_oralis | 4.3% | Corynebacterium_striatum;Acinetobacter_baumannii |  |
| CU5 | BALF |  | Mycoplasma_hominis | 29.4% | Pseudomonas_aeruginosa |  |
| CU5 | BALF |  | Pseudomonas_aeruginosa | 58.8% | Pseudomonas_aeruginosa |  |
| CU6 | BALF |  | Pseudomonas_aeruginosa | 61.9% | Pseudomonas_aeruginosa |  |
| CV3 | BALF | Haemophilus_influenzae | Haemophilus_influenzae | 93.2% | Pseudomonas_aeruginosa;Haemophilus_influenzae |  |
| CV10 | BALF |  | Pseudomonas_aeruginosa | 9.5% | Pseudomonas_aeruginosa | Corynebacterium_striatum;Acinetobacter_baumannii |
| CV14 | Sputum |  | Corynebacterium_striatum | 87.7% | Corynebacterium_striatum | Acinetobacter_baumannii |
| CW2 | BALF | Acinetobacter_baumannii;Escherichia_coli | Acinetobacter_baumannii | 97.2% | Acinetobacter_baumannii | Escherichia_coli |
| CW2 | BALF | Acinetobacter_baumannii;Escherichia_coli | Escherichia_coli | 2.8% | Acinetobacter_baumannii | Escherichia_coli |
| CY4 | BALF | Stenotrophomonas_maltophilia;Acinetobacter_baumannii | Stenotrophomonas_maltophilia | 93.6% | Stenotrophomonas_maltophilia |  |
| CZ4 | BALF |  | Pseudomonas_aeruginosa | 30.0% | Pseudomonas_aeruginosa |  |
| DA3 | BALF | Stenotrophomonas_maltophilia;Pseudomonas_aeruginosa;Achromobacter_xylosoxidans | Achromobacter_xylosoxidans | 29.6% | Pseudomonas_aeruginosa;Stenotrophomonas_maltophilia;Achromobacter_xylosoxidans |  |
| DA3 | BALF | Stenotrophomonas_maltophilia;Pseudomonas_aeruginosa;Achromobacter_xylosoxidans | Pseudomonas_aeruginosa | 61.4% | Pseudomonas_aeruginosa;Stenotrophomonas_maltophilia;Achromobacter_xylosoxidans |  |
| DC11 | Sputum | Pseudomonas_aeruginosa | Pseudomonas_aeruginosa | 95.9% | Pseudomonas_aeruginosa |  |
| DC7 | Sputum |  | Nocardia_brasiliensis | 22.4% |  | Pseudomonas_aeruginosa |
| DC7 | Sputum |  | Nocardia_farcinica | 9.7% |  | Pseudomonas_aeruginosa |
| DC7 | Sputum |  | Veillonella_atypica | 9.7% |  | Pseudomonas_aeruginosa |
| DC7 | Sputum |  | Veillonella_parvula | 17.2% |  | Pseudomonas_aeruginosa |
| DC8 | Sputum | Klebsiella_pneumoniae;Pseudomonas_aeruginosa | Achromobacter_xylosoxidans | 20.8% | Achromobacter_xylosoxidans | Pseudomonas_aeruginosa;Stenotrophomonas_maltophilia |
| DC8 | Sputum | Klebsiella_pneumoniae;Pseudomonas_aeruginosa | Elizabethkingia_anophelis | 31.2% | Achromobacter_xylosoxidans | Pseudomonas_aeruginosa;Stenotrophomonas_maltophilia |
| DA10 | BALF | Pseudomonas_aeruginosa;Acinetobacter_baumannii | Acinetobacter_baumannii | 91.5% | Pseudomonas_aeruginosa;Acinetobacter_baumannii;Burkholderia_cepacia |  |
| DE3 | BALF |  | Prevotella_melaninogenica | 10.3% |  | Pseudomonas_aeruginosa |
| DE3 | BALF |  | Rothia_mucilaginosa | 8.6% |  | Pseudomonas_aeruginosa |
| DE5 | Sputum | Pseudomonas_aeruginosa | Pseudomonas_aeruginosa | 84.6% | Pseudomonas_aeruginosa |  |
| DE5 | Sputum | Pseudomonas_aeruginosa | Streptococcus_anginosus | 5.0% | Pseudomonas_aeruginosa |  |
| DE6 | Sputum | Acinetobacter_baumannii;Pseudomonas_aeruginosa | Pseudomonas_aeruginosa | 96.4% | Pseudomonas_aeruginosa | Acinetobacter_baumannii |
| DD2 | BALF | Pseudomonas_aeruginosa | Pseudomonas_aeruginosa | 68.5% | Pseudomonas_aeruginosa | Klebsiella_pneumoniae |
| DD10 | Sputum | Klebsiella_pneumoniae | Bifidobacterium_longum | 19.6% |  | Klebsiella_pneumoniae;Streptococcus_pneumoniae |
| DD10 | Sputum | Klebsiella_pneumoniae | Streptococcus_parasanguinis | 20.8% |  | Klebsiella_pneumoniae;Streptococcus_pneumoniae |
| DD12 | Sputum |  | Enterococcus_faecium | 6.4% | Enterococcus_faecium |  |
| DD12 | Sputum |  | Rothia_mucilaginosa | 70.8% | Enterococcus_faecium |  |
| DD12 | Sputum |  | Staphylococcus_epidermidis | 21.3% | Enterococcus_faecium |  |
| DF9 | BALF | Klebsiella_pneumoniae | Klebsiella_pneumoniae | 99.2% | Klebsiella_pneumoniae |  |
| DJ14 | Sputum |  | Haemophilus_parainfluenzae | 8.1% |  |  |
| DJ14 | Sputum |  | Neisseria_meningitidis | 29.7% |  |  |
| DJ14 | Sputum |  | Parvimonas_micra | 28.8% |  |  |
| DK4 | BALF | Achromobacter_xylosoxidans | Achromobacter_xylosoxidans | 52.9% | Achromobacter_xylosoxidans | Pseudomonas_aeruginosa |
| DK6 | BALF |  | Streptococcus_pneumoniae | 80.0% | Pseudomonas_aeruginosa;Streptococcus_pneumoniae |  |
| DK15 | BALF |  | Corynebacterium_striatum | 3.8% |  | Corynebacterium_striatum |
| DK15 | BALF |  | Staphylococcus_epidermidis | 58.7% |  | Corynebacterium_striatum |
| DK16 | BALF |  | Pseudomonas_aeruginosa | 71.4% |  | Pseudomonas_aeruginosa |
| DI8 | Sputum |  | Achromobacter_xylosoxidans | 17.1% | Pseudomonas_aeruginosa;Corynebacterium_striatum;Stenotrophomonas_maltophilia;Achromobacter_xylosoxidans |  |
| DI8 | Sputum |  | Corynebacterium_striatum | 49.6% | Pseudomonas_aeruginosa;Corynebacterium_striatum;Stenotrophomonas_maltophilia;Achromobacter_xylosoxidans |  |
| DI8 | Sputum |  | Pseudomonas_aeruginosa | 20.0% | Pseudomonas_aeruginosa;Corynebacterium_striatum;Stenotrophomonas_maltophilia;Achromobacter_xylosoxidans |  |
| DJ8 | Sputum |  | Prevotella_melaninogenica | 19.3% |  |  |
| DJ8 | Sputum |  | Streptococcus_parasanguinis | 38.0% |  |  |
| DJ8 | Sputum |  | Veillonella_parvula | 7.6% |  |  |
| DG6 | Sputum | Pseudomonas_aeruginosa | Pseudomonas_aeruginosa | 99.0% | Pseudomonas_aeruginosa |  |
| DH8 | Sputum | Acinetobacter_baumannii;Enterobacter_cloacae | Acinetobacter_baumannii | 7.1% |  | Acinetobacter_baumannii;Achromobacter_xylosoxidans |
| DH8 | Sputum | Acinetobacter_baumannii;Enterobacter_cloacae | Pseudopropionibacterium_propionicum | 18.6% |  | Acinetobacter_baumannii;Achromobacter_xylosoxidans |
| DI4 | BALF |  | Moraxella_catarrhalis | 24.0% | Pseudomonas_aeruginosa;Moraxella_catarrhalis |  |
| DI4 | BALF |  | Rothia_mucilaginosa | 8.4% | Pseudomonas_aeruginosa;Moraxella_catarrhalis |  |
| DI4 | BALF |  | Veillonella_parvula | 10.2% | Pseudomonas_aeruginosa;Moraxella_catarrhalis |  |
| DK111 | Sputum | Streptococcus_pneumoniae | Corynebacterium_argentoratense | 24.2% | Streptococcus_pneumoniae |  |
| DK111 | Sputum | Streptococcus_pneumoniae | Streptococcus_mitis | 20.0% | Streptococcus_pneumoniae |  |
| DK111 | Sputum | Streptococcus_pneumoniae | Streptococcus_pneumoniae | 12.3% | Streptococcus_pneumoniae |  |
| DL13 | Sputum |  | Corynebacterium_striatum | 12.4% | Corynebacterium_striatum;Enterococcus_faecalis | Acinetobacter_baumannii |
| DL13 | Sputum |  | Enterococcus_faecalis | 10.0% | Corynebacterium_striatum;Enterococcus_faecalis | Acinetobacter_baumannii |
| DL13 | Sputum |  | Rothia_mucilaginosa | 71.9% | Corynebacterium_striatum;Enterococcus_faecalis | Acinetobacter_baumannii |
| DL16 | Sputum |  | Prevotella_intermedia | 19.2% |  | Streptococcus_pneumoniae;Acinetobacter_baumannii;Pseudomonas_aeruginosa |
| DL16 | Sputum |  | Pseudomonas_aeruginosa | 2.3% |  | Streptococcus_pneumoniae;Acinetobacter_baumannii;Pseudomonas_aeruginosa |
| DL16 | Sputum |  | Rothia_mucilaginosa | 13.1% |  | Streptococcus_pneumoniae;Acinetobacter_baumannii;Pseudomonas_aeruginosa |
| DM1 | BALF | Klebsiella_pneumoniae;Pseudomonas_aeruginosa;Acinetobacter_baumannii | Acinetobacter_baumannii | 63.7% | Corynebacterium_striatum;Klebsiella_pneumoniae;Stenotrophomonas_maltophilia;Acinetobacter_baumannii;Pseudomonas_aeruginosa |  |
| DM1 | BALF | Klebsiella_pneumoniae;Pseudomonas_aeruginosa;Acinetobacter_baumannii | Pseudomonas_aeruginosa | 23.0% | Corynebacterium_striatum;Klebsiella_pneumoniae;Stenotrophomonas_maltophilia;Acinetobacter_baumannii;Pseudomonas_aeruginosa |  |
| DM5 | Sputum | Klebsiella_pneumoniae | Haemophilus_parainfluenzae | 4.4% | Klebsiella_pneumoniae;Pseudomonas_aeruginosa | Acinetobacter_baumannii |
| DM5 | Sputum | Klebsiella_pneumoniae | Pseudomonas_aeruginosa | 83.0% | Klebsiella_pneumoniae;Pseudomonas_aeruginosa | Acinetobacter_baumannii |
| DM9 | Sputum |  | Haemophilus_influenzae | 37.5% | Haemophilus_influenzae | Acinetobacter_baumannii |
| DM9 | Sputum |  | Streptococcus_oralis | 12.5% | Haemophilus_influenzae | Acinetobacter_baumannii |
| DN7 | BALF |  | Pseudomonas_aeruginosa | 44.0% |  | Corynebacterium_striatum;Staphylococcus_aureus;Pseudomonas_aeruginosa |
| DN10 | Sputum | Klebsiella_pneumoniae | Moraxella_catarrhalis | 88.8% | Moraxella_catarrhalis |  |
| DN10 | Sputum | Klebsiella_pneumoniae | Rothia_mucilaginosa | 1.2% | Moraxella_catarrhalis |  |
| DN11 | Sputum | Staphylococcus_aureus;Acinetobacter_baumannii | Corynebacterium_striatum | 46.9% | Corynebacterium_striatum;Staphylococcus_aureus |  |
| DN11 | Sputum | Staphylococcus_aureus;Acinetobacter_baumannii | Prevotella_melaninogenica | 2.3% | Corynebacterium_striatum;Staphylococcus_aureus |  |
| DN11 | Sputum | Staphylococcus_aureus;Acinetobacter_baumannii | Staphylococcus_aureus | 43.7% | Corynebacterium_striatum;Staphylococcus_aureus |  |
| DO9 | Sputum | Acinetobacter_baumannii | Acinetobacter_baumannii | 99.0% | Acinetobacter_baumannii |  |
| DO10 | Sputum |  | Bacteroides_zoogleoformans | 8.9% |  |  |
| DO10 | Sputum |  | Parvimonas_micra | 6.2% |  |  |
| DO10 | Sputum |  | Prevotella_intermedia | 69.3% |  |  |
| DT2 | BALF | Corynebacterium_striatum | Corynebacterium_striatum | 79.8% | Corynebacterium_striatum | Acinetobacter_baumannii;Pseudomonas_aeruginosa |
| DU10 | Sputum |  | Prevotella_melaninogenica | 24.0% |  | Corynebacterium_striatum;Haemophilus_influenzae;Streptococcus_pneumoniae |
| DU10 | Sputum |  | Streptococcus_mitis | 10.6% |  | Corynebacterium_striatum;Haemophilus_influenzae;Streptococcus_pneumoniae |
| DU10 | Sputum |  | Veillonella_parvula | 13.8% |  | Corynebacterium_striatum;Haemophilus_influenzae;Streptococcus_pneumoniae |
| DV1 | BALF | Haemophilus_influenzae | Haemophilus_influenzae | 88.4% |  |  |
| DV1 | BALF | Haemophilus_influenzae | Mycobacterium_intracellulare | 8.1% |  |  |
| DV2 | BALF |  | Olsenella_uli | 8.0% |  | Corynebacterium_striatum;Pseudomonas_aeruginosa |
| DV2 | BALF |  | Parvimonas_micra | 62.1% |  | Corynebacterium_striatum;Pseudomonas_aeruginosa |
| DV2 | BALF |  | Prevotella_intermedia | 10.3% |  | Corynebacterium_striatum;Pseudomonas_aeruginosa |
| DW9 | BALF | Escherichia_coli | Escherichia_coli | 100.0% |  |  |
| DX6 | BALF | Acinetobacter_baumannii | Acinetobacter_baumannii | 97.9% |  | Pseudomonas_aeruginosa |
| DX11 | BALF | Pseudomonas_aeruginosa | Pseudomonas_aeruginosa | 96.3% |  |  |
| DX11 | BALF | Pseudomonas_aeruginosa | Streptococcus_oralis | 1.6% |  |  |
| DX12 | BALF | Acinetobacter_baumannii;Burkholderia_cepacia | Acinetobacter_baumannii | 4.8% |  |  |
| DX12 | BALF | Acinetobacter_baumannii;Burkholderia_cepacia | Burkholderia_cepacia | 93.9% |  |  |
| EA4 | BALF |  | Pseudomonas_aeruginosa | 50.0% |  | Corynebacterium_striatum;Klebsiella_pneumoniae;Pseudomonas_aeruginosa |
| EB2 | BALF |  | Moraxella_catarrhalis | 97.0% | Moraxella_catarrhalis | Klebsiella_pneumoniae;Pseudomonas_aeruginosa |
| EB10 | Sputum | Corynebacterium_striatum | Corynebacterium_striatum | 86.8% |  |  |
| EB10 | Sputum | Corynebacterium_striatum | Streptococcus_oralis | 6.3% |  |  |
| EC5 | BALF |  | Haemophilus_influenzae | 80.6% | Haemophilus_influenzae;Pseudomonas_aeruginosa |  |
| EE1 | BALF | Burkholderia_cepacia | Burkholderia_cepacia | 92.5% |  | Pseudomonas_aeruginosa |
| EE3 | BALF |  | Prevotella_melaninogenica | 10.4% |  | Pseudomonas_aeruginosa |
| EE3 | BALF |  | Rothia_mucilaginosa | 26.4% |  | Pseudomonas_aeruginosa |
| EE3 | BALF |  | Streptococcus_parasanguinis | 11.8% |  | Pseudomonas_aeruginosa |
| EE4 | BALF |  | Parvimonas_micra | 20.3% |  | Acinetobacter_baumannii |
| EE4 | BALF |  | Tannerella_forsythia | 50.8% |  | Acinetobacter_baumannii |
| EH6 | BALF | Acinetobacter_baumannii | Acinetobacter_baumannii | 98.6% |  |  |
| EK1 | BALF | Acinetobacter_baumannii;Pseudomonas_aeruginosa | Acinetobacter_baumannii | 99.3% | Acinetobacter_baumannii;Pseudomonas_aeruginosa |  |
| EK8 | Sputum | Pseudomonas_aeruginosa | Prevotella_melaninogenica | 9.7% |  |  |
| EK8 | Sputum | Pseudomonas_aeruginosa | Pseudomonas_aeruginosa | 23.2% |  |  |
| EK2 | BALF | Klebsiella_pneumoniae | Klebsiella_pneumoniae | 37.9% | Acinetobacter_baumannii | Pseudomonas_aeruginosa;Stenotrophomonas_maltophilia |
| EL6 | BALF | Pseudomonas_aeruginosa;Acinetobacter_baumannii | Acinetobacter_baumannii | 98.1% |  |  |
| EL6 | BALF | Pseudomonas_aeruginosa;Acinetobacter_baumannii | Pseudomonas_aeruginosa | 1.9% |  |  |
| EL18 | Sputum |  | Corynebacterium_argentoratense | 15.5% |  | Acinetobacter_baumannii;Streptococcus_pneumoniae |
| EL18 | Sputum |  | Rothia_mucilaginosa | 28.0% |  | Acinetobacter_baumannii;Streptococcus_pneumoniae |
| EL18 | Sputum |  | Streptococcus_parasanguinis | 12.5% |  | Acinetobacter_baumannii;Streptococcus_pneumoniae |
| EM5 | BALF |  | Streptococcus_pneumoniae | 58.3% | Streptococcus_pneumoniae | Pseudomonas_aeruginosa;Acinetobacter_baumannii |
| EO3 | Sputum |  | Corynebacterium_resistens | 18.3% |  | Stenotrophomonas_maltophilia;Enterococcus_faecium |
| EO3 | Sputum |  | Enterococcus_faecium | 21.3% |  | Stenotrophomonas_maltophilia;Enterococcus_faecium |
| EO3 | Sputum |  | Staphylococcus_haemolyticus | 23.0% |  | Stenotrophomonas_maltophilia;Enterococcus_faecium |
| EO3 | Sputum |  | Stenotrophomonas_maltophilia | 25.3% |  | Stenotrophomonas_maltophilia;Enterococcus_faecium |
| ET3 | BALF |  | Fusobacterium_periodonticum | 6.0% |  |  |
| ET3 | BALF |  | Nocardia_brasiliensis | 23.8% |  |  |
| ET4 | BALF | Klebsiella_oxytoca;Leclercia_adecarboxylata | Klebsiella_oxytoca | 24.3% |  | Pseudomonas_aeruginosa |
| ET4 | BALF | Klebsiella_oxytoca;Leclercia_adecarboxylata | Staphylococcus_epidermidis | 13.6% |  | Pseudomonas_aeruginosa |
| ET4 | BALF | Klebsiella_oxytoca;Leclercia_adecarboxylata | Staphylococcus_haemolyticus | 49.6% |  | Pseudomonas_aeruginosa |
| EU7 | Sputum | Acinetobacter_baumannii | Acinetobacter_baumannii | 65.8% |  | Corynebacterium_striatum |
| EU7 | Sputum | Acinetobacter_baumannii | Corynebacterium_striatum | 28.1% |  | Corynebacterium_striatum |
| EU7 | Sputum | Acinetobacter_baumannii | Streptococcus_constellatus | 2.9% |  | Corynebacterium_striatum |
| EV1 | BALF | Pseudomonas_aeruginosa;Enterobacter_aerogenes | Enterobacter_aerogenes | 56.1% | Enterobacter_aerogenes;Enterococcus_faecalis | Pseudomonas_aeruginosa |
| EV1 | BALF | Pseudomonas_aeruginosa;Enterobacter_aerogenes | Enterococcus_faecalis | 37.6% | Enterobacter_aerogenes;Enterococcus_faecalis | Pseudomonas_aeruginosa |
| EV1 | BALF | Pseudomonas_aeruginosa;Enterobacter_aerogenes | Weissella_cibaria | 1.7% | Enterobacter_aerogenes;Enterococcus_faecalis | Pseudomonas_aeruginosa |
| EX1 | BALF | Klebsiella_pneumoniae | Enterococcus_faecium | 63.3% |  | Pseudomonas_aeruginosa;Enterococcus_faecium |
| EX1 | BALF | Klebsiella_pneumoniae | Klebsiella_pneumoniae | 2.0% |  | Pseudomonas_aeruginosa;Enterococcus_faecium |
| EX1 | BALF | Klebsiella_pneumoniae | Pseudomonas_aeruginosa | 2.0% |  | Pseudomonas_aeruginosa;Enterococcus_faecium |
| EX1 | BALF | Klebsiella_pneumoniae | Streptococcus_oralis | 14.0% |  | Pseudomonas_aeruginosa;Enterococcus_faecium |
| EX6 | Sputum | Pseudomonas_aeruginosa | Prevotella_jejuni | 10.1% |  |  |
| EX6 | Sputum | Pseudomonas_aeruginosa | Prevotella_melaninogenica | 13.0% |  |  |
| EX6 | Sputum | Pseudomonas_aeruginosa | Pseudomonas_aeruginosa | 24.6% |  |  |
| EX6 | Sputum | Pseudomonas_aeruginosa | Veillonella_atypica | 17.4% |  |  |
| EY2 | BALF | Burkholderia_cepacia | Burkholderia_cepacia | 93.8% |  |  |
| FB01 | BALF |  | Porphyromonas_gingivalis | 16.5% |  |  |
| FB01 | BALF |  | Tannerella_forsythia | 76.3% |  |  |
| FB01 | BALF |  | Treponema_denticola | 4.0% |  |  |
| FD2 | BALF |  | Prevotella_melaninogenica | 12.8% |  | Pseudomonas_aeruginosa;Stenotrophomonas_maltophilia |
| FD2 | BALF |  | Pseudomonas_aeruginosa | 14.9% |  | Pseudomonas_aeruginosa;Stenotrophomonas_maltophilia |
| FE3 | BALF | Pseudomonas_aeruginosa | Elizabethkingia_anophelis | 96.4% |  | Pseudomonas_aeruginosa |
| FE3 | BALF | Pseudomonas_aeruginosa | Pseudomonas_aeruginosa | 1.2% |  | Pseudomonas_aeruginosa |
| FF2 | BALF |  | Corynebacterium_striatum | 11.1% |  | Pseudomonas_aeruginosa;Stenotrophomonas_maltophilia;Corynebacterium_striatum |
| FF03 | BALF | Pseudomonas_aeruginosa;Klebsiella_pneumoniae;Escherichia_coli | Escherichia_coli | 3.1% |  |  |
| FF03 | BALF | Pseudomonas_aeruginosa;Klebsiella_pneumoniae;Escherichia_coli | Pseudomonas_aeruginosa | 94.4% |  |  |
| FF04 | BALF | Acinetobacter_baumannii;Burkholderia_multivorans | Acinetobacter_baumannii | 9.4% |  |  |
| FF04 | BALF | Acinetobacter_baumannii;Burkholderia_multivorans | Burkholderia_multivorans | 87.7% |  |  |
| FF8 | BALF |  | Moraxella_catarrhalis | 12.5% | Moraxella_catarrhalis | Pseudomonas_aeruginosa;Stenotrophomonas_maltophilia;Burkholderia_multivorans |
