# Supplementary material for: Prospective Evaluation of a Rapid Clinical Metagenomics Test for Bacterial Pneumonia
Source: Front Cell Infect Microbiol. 2021 Oct 19;11:684965. doi: 10.3389/fcimb.2021.684965 (PMC8560692; doi:10.3389/fcimb.2021.684965)
Supplement: Supplementary file 4 [file DataSheet_4.docx]

| species reported in this study | average abundance | median abundance | standard deviation of abundance | frequency |
| --- | --- | --- | --- | --- |
| Pseudomonas_aeruginosa | 49.7% | 50.0% | 34.1% | 81 |
| Corynebacterium_striatum | 46.4% | 48.6% | 30.8% | 35 |
| Acinetobacter_baumannii | 65.0% | 80.9% | 36.8% | 32 |
| Rothia_mucilaginosa | 29.1% | 21.5% | 22.6% | 29 |
| Streptococcus_pneumoniae | 34.9% | 11.8% | 37.0% | 16 |
| Veillonella_parvula | 13.1% | 13.8% | 6.6% | 15 |
| Stenotrophomonas_maltophilia | 20.1% | 13.6% | 25.2% | 13 |
| Streptococcus_parasanguinis | 17.2% | 15.4% | 9.0% | 13 |
| Haemophilus_influenzae | 56.8% | 60.2% | 33.3% | 12 |
| Klebsiella_pneumoniae | 39.1% | 24.3% | 37.8% | 12 |
| Parvimonas_micra | 28.2% | 18.1% | 24.9% | 12 |
| Streptococcus_mitis | 22.2% | 19.5% | 17.1% | 12 |
| Enterococcus_faecium | 41.3% | 29.0% | 31.9% | 11 |
| Prevotella_melaninogenica | 14.6% | 12.8% | 8.4% | 11 |
| Streptococcus_oralis | 15.1% | 11.8% | 14.9% | 11 |
| Moraxella_catarrhalis | 60.6% | 87.9% | 38.4% | 9 |
| Burkholderia_cepacia | 75.1% | 93.9% | 38.3% | 8 |
| Staphylococcus_aureus | 43.1% | 37.0% | 35.4% | 8 |
| Staphylococcus_epidermidis | 27.6% | 20.4% | 19.6% | 6 |
| Veillonella_atypica | 15.2% | 15.2% | 6.0% | 6 |
| Achromobacter_xylosoxidans | 41.1% | 29.6% | 28.4% | 5 |
| Enterococcus_faecalis | 19.3% | 17.5% | 14.9% | 4 |
| Porphyromonas_gingivalis | 38.1% | 39.2% | 19.8% | 4 |
| Prevotella_intermedia | 25.6% | 14.8% | 29.8% | 4 |
| Staphylococcus_haemolyticus | 23.0% | 18.8% | 19.2% | 4 |
| Tannerella_forsythia | 45.7% | 41.5% | 23.3% | 4 |
| Escherichia_coli | 35.3% | 3.1% | 56.0% | 3 |
| Prevotella_jejuni | 21.3% | 10.9% | 18.6% | 3 |
| Streptococcus_constellatus | 7.9% | 8.1% | 5.0% | 3 |
| Streptococcus_salivarius | 14.4% | 14.5% | 3.9% | 3 |
| Actinomyces_pacaensis | 8.7% | 8.7% | 4.9% | 2 |
| Bacteroides_heparinolyticus | 29.8% | 29.8% | 1.7% | 2 |
| Corynebacterium_argentoratense | 19.8% | 19.8% | 6.2% | 2 |
| Elizabethkingia_anophelis | 63.8% | 63.8% | 46.1% | 2 |
| Filifactor_alocis | 6.1% | 6.1% | 2.5% | 2 |
| Haemophilus_parainfluenzae | 6.3% | 6.3% | 2.6% | 2 |
| Lactobacillus_paracasei | 15.3% | 15.3% | 6.6% | 2 |
| Lactobacillus_rhamnosus | 19.8% | 19.8% | 6.7% | 2 |
| Nocardia_brasiliensis | 23.1% | 23.1% | 1.0% | 2 |
| Rhodococcus_qingshengii | 10.7% | 10.7% | 0.9% | 2 |
| Streptococcus_anginosus | 3.9% | 3.9% | 1.6% | 2 |
| Treponema_denticola | 17.0% | 17.0% | 18.4% | 2 |
| Acinetobacter_pittii | 80.4% | 80.4% |  | 1 |
| Bacteroides_cellulosilyticus | 5.9% | 5.9% |  | 1 |
| Bacteroides_fragilis | 48.2% | 48.2% |  | 1 |
| Bacteroides_zoogleoformans | 8.9% | 8.9% |  | 1 |
| Bifidobacterium_breve | 27.0% | 27.0% |  | 1 |
| Bifidobacterium_longum | 19.6% | 19.6% |  | 1 |
| Burkholderia_multivorans | 87.7% | 87.7% |  | 1 |
| Capnocytophaga_leadbetteri | 5.1% | 5.1% |  | 1 |
| Chlamydia_psittaci | 94.8% | 94.8% |  | 1 |
| Corynebacterium_resistens | 18.3% | 18.3% |  | 1 |
| Corynebacterium_simulans | 84.7% | 84.7% |  | 1 |
| Enterobacter_aerogenes | 56.1% | 56.1% |  | 1 |
| Enterobacter_cloacae | 3.2% | 3.2% |  | 1 |
| Fusobacterium_necrophorum | 13.2% | 13.2% |  | 1 |
| Fusobacterium_nucleatum | 26.3% | 26.3% |  | 1 |
| Fusobacterium_periodonticum | 6.0% | 6.0% |  | 1 |
| Klebsiella_oxytoca | 24.3% | 24.3% |  | 1 |
| Lactobacillus_crispatus | 11.0% | 11.0% |  | 1 |
| Lactobacillus_gasseri | 7.2% | 7.2% |  | 1 |
| Lactobacillus_mucosae | 1.3% | 1.3% |  | 1 |
| Lactobacillus_pentosus | 14.2% | 14.2% |  | 1 |
| Lactobacillus_reuteri | 14.6% | 14.6% |  | 1 |
| Mycobacterium_intracellulare | 8.1% | 8.1% |  | 1 |
| Mycobacterium_tuberculosis | 91.4% | 91.4% |  | 1 |
| Mycoplasma_hominis | 29.4% | 29.4% |  | 1 |
| Mycoplasma_pneumoniae | 65.2% | 65.2% |  | 1 |
| Neisseria_meningitidis | 29.7% | 29.7% |  | 1 |
| Nocardia_farcinica | 9.7% | 9.7% |  | 1 |
| Olsenella_uli | 8.0% | 8.0% |  | 1 |
| Parascardovia_denticolens | 20.7% | 20.7% |  | 1 |
| Prevotella_denticola | 53.8% | 53.8% |  | 1 |
| Pseudomonas_mendocina | 97.3% | 97.3% |  | 1 |
| Pseudopropionibacterium_propionicum | 18.6% | 18.6% |  | 1 |
| Rothia_dentocariosa | 10.6% | 10.6% |  | 1 |
| Serratia_marcescens | 32.1% | 32.1% |  | 1 |
| Streptococcus_gordonii | 14.3% | 14.3% |  | 1 |
| Streptococcus_pseudopneumoniae | 12.1% | 12.1% |  | 1 |
| Weissella_cibaria | 1.7% | 1.7% |  | 1 |
